# Visfatin/eNampt induces endothelial dysfunction *in vivo*: a role for Toll-Like Receptor 4 and NLRP3 inflammasome

**DOI:** 10.1038/s41598-020-62190-w

**Published:** 2020-03-25

**Authors:** Tania Romacho, Inés Valencia, Mariella Ramos-González, Susana Vallejo, Miguel López-Esteban, Oscar Lorenzo, Pablo Cannata, Alejandra Romero, Alvaro San Hipólito-Luengo, Jorge F. Gómez-Cerezo, Concepción Peiró, Carlos F. Sánchez-Ferrer

**Affiliations:** 1 Departamento de Farmacología y Terapéutica, Facultad de Medicina, Universidad Autónoma de Madrid, Madrid, Spain; 2 Instituto de Investigación Sanitaria del Hospital Universitario La Paz (IdiPAZ), Madrid, Spain; 3 PhD Programme in Pharmacology and Physiology, Doctoral School, Universidad Autónoma de Madrid, Madrid, Spain; 4 Departamento de Medicina, Facultad de Medicina, Universidad Autónoma de Madrid, Madrid, Spain; 5 Instituto de Investigación Sanitaria Fundación Jiménez Díaz, Madrid, Spain; 6 Servicio de Medicina Interna, Hospital Universitario Infanta Sofía, Madrid, Spain

**Keywords:** Vascular diseases, Inflammasome

## Abstract

Visfatin/extracellular-nicotinamide-phosphoribosyltranferase-(eNampt) is a multifaceted adipokine enhanced in type-2-diabetes and obesity. Visfatin/eNampt cause *in vitro* endothelial dysfunction and vascular inflammation, although whether the same effects are achieved *in vivo* is unknown. Toll-like receptor-4 (TLR4), a main surface pattern recognition receptor of innate immune system is a potential target for visfatin/eNampt. We studied its capacity to generate vascular dysfunction *in vivo*, focusing on TLR4 role and downstream activation of nod-like-receptor-protein-3 (NLRP3)-inflammasome. 4 month-old C57BL/6 mice were exposed to 7 days infusion of visfatin/eNampt, alone or together with FK 866 (Nampt enzymatic inhibitor), CLI 095 (TLR4 blocker), MCC 950 (NLRP3-inflammasome inhibitor), or anakinra (interleukin(IL)-1-receptor antagonist). Endothelial dysfunction was tested in isolated microvessels. In human umbilical endothelial cells (HUVEC), proteins related to the NLRP3-inflammasome phosphorylated p-65, NLRP3, caspase-1, pro-IL-1β, and mature IL-1β were determined by Western blot, while the inflammasome related apoptosis-associated speck-like protein containing a caspase recruitment domain (ASC-specks) was studied by immunofluorescence. Impaired endothelium-dependent relaxations were observed in isolated mesenteric microvessels from visfatin/eNampt-infused mice. This effect was attenuated by co-treatment with FK 866 or CLI 095, supporting a role for Nampt enzymatic activity and TLR4 activation. Moreover, cultured HUVEC exposed to visfatin/eNampt showed higher expression and activation of NLRP3-inflammasome. Again, this effect relied on Nampt enzymatic activity and TLR4 activation, and it was abrogated by the inflammasome assembly blockade with MCC 950. The endothelial dysfunction evoked by visfatin/eNampt infusion *in vivo* was also sensitive to both MCC 950 and anakinra treatments, suggesting that the NLRP3-inflammasome-driven tissular release of IL-1β is the final mediator of endothelial damage. We conclude that Visfatin/eNampt produces *in vivo* vascular dysfunction in mice by a Nampt-dependent TLR4-mediated pathway, involving NLRP3-inflammasome and paracrine IL-1β. Thus, those targets may become therapeutic strategies for attenuating the adipokine-mediated vascular dysfunction associated to obesity and/or type-2-diabetes.

## Introduction

Visfatin was firstly identified as an adipocytokine mainly released by visceral adipose tissue^1^. Visfatin was shortly after found to be identical to pre-B cell colony enhancing factor (PBEF), and to the enzyme nicotinamide phosphoribosyltranferase (Nampt)^2,3^. Nampt transforms nicotinamide into nicotinamide mononucleotide (NMN), which is then converted into NAD^+^ by nicotinamide/nicotinic acid mononucleotide adenylyltransferase (Nmnat)^4,5^. Two isoforms of Nampt are currently acknowledged. The intracellular form of Nampt (iNampt) is the rate limiting-enzyme for NAD^+^ biosynthesis in cells from mammals^1^. The extracellular form of Nampt (eNampt), object of the present study, is considered as a multifunctional cytokine-like molecule that is synthesized and released by adipocytes, but also by other cell types including vascular cells exposed to pro-inflammatory stimuli^6,7^. To date, no specific receptor has been identified for visfatin/eNampt, while some of its actions have been attributed to its intrinsic Nampt enzymatic activity^8–12^. Thus, Toll-like receptor-4 (TLR4) has been recently proposed as mediating some harmful actions of visfatin/eNampt in lung endothelial cells^13^. TLR4 is a main cell membrane pattern recognition receptor of the innate immune system that generates pro-inflammatory signals, including the activation of nod-like receptor protein 3 (NLRP3), as the best characterized sensor protein of the inflammasome pathway^14^.

Cardiovascular diseases are among the leading causes of disease in type 2 diabetic and obese patients. Atherosclerosis, as a chronic pro-inflammatory vascular disease, is usually at the basis of the cardiovascular events present in these patients^15^. Endothelial dysfunction is one of the earliest markers of atherosclerosis and cardiovascular diseases^16^. Such dysfunctional endothelium promotes the disruption of vascular homeostasis and impairs vasomotor responses, which eventually results in defective vasorelaxation rendering the vessels prone to inflammation and atherosclerosis. In type 2 diabetic patients increased circulating visfatin/eNampt levels have been associated with defective flow-mediated dilation^17^ and the marker of endothelial dysfunction homocysteine^18^. Besides being regarded as a circulating biomarker for cardiovascular complications, visfatin/eNampt can exert some direct deleterious actions on vascular cells, including proliferation, inflammation, apoptosis, and premature senescence^12,19–21^ as well as impaired microvascular endothelium-dependent vasorelaxation *ex vivo*^11,22^. However, whether visfatin/eNampt is capable of causing endothelial dysfunction and end organ damage in a more complex *scenario* such as *in vivo*, remains to be determined.

Therefore, in the present study, we assessed whether the *in vivo* infusion of visfatin/eNampt in mice could induce early vascular dysfunction, identifying the possible underlying mechanisms involved, such as the participation of Nampt activity, the activation of the TLR4 receptor, as well as the involvement of NLRP3 inflammasome.

## Results

### Visfatin/eNampt induces endothelial dysfunction in murine mesenteric microvessels *ex vivo* and *in vivo* via Nampt enzymatic activity

**Figure 1:**
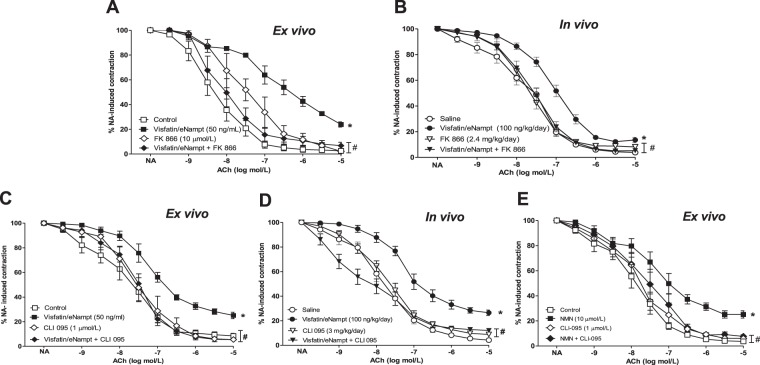
(**A**) Isolated mesenteric microvessels obtained from untreated C57BL/6 mice were pre-contracted with 3 μmol/L noradrenaline (NA) and submitted to cumulative concentrations of the endothelium-dependent vasodilator acetylcholine (ACh, 10 nmol/L to 10 μmol/L) in the absence of any previous treatment (Control) or after receiving 50 ng/mL visfatin/eNampt, 10 µmolL FK 866, or both visfatin/eNampt plus FK 866. (**B**) Isolated mesenteric microvessels were obtained from C57BL/6 mice infused during 7 days with saline solution, visfatin/eNampt (100 ng/kg/day), FK 866 (2.4 mg/kg/day) or both visfatin/eNampt plus FK 866, through the subcutaneous implantation of osmotic mini-pumps. The isolated vessels were also pre-contracted with NA and submitted to cumulative concentrations of ACh. (**C)** Isolated mesenteric microvessels obtained from untreated C57BL/6 mice submitted to NA and ACh in Control conditions or after receiving 50 ng/mL visfatin/eNampt, 1 µmol/L CLI 095, or both visfatin/eNampt plus CLI 095. **(D)** Isolated mesenteric microvessels from C57BL/6 mice infused with saline solution or visfatin/eNampt (100 ng/kg/day) for 7 days, and receiving also the i.p. administration of CLI 095 (3 mg/kg/day) or analogous amounts of saline during the 7 days. **(E)** Isolated mesenteric microvessels obtained from untreated C57BL/6 mice pre-contracted with NA and submitted to ACh in Control conditions or after receiving 10 µmol/L nicotinamide mononucleotide (NMN), 1 µmolL CLI 095, or both NMN plus CLI 095. The curves (mean ± SEM) are expressed as the percentage of the previous NA-evoked contraction, which is indicated in the Tables S1 and S2, as well as the respective pEC_50_ values. For every curve, 4 to 15 segments were used, obtained from 3 to 8 different animals. *p < 0.05 vs respective control or saline. #p < 0.05 vs respective visfatin/eNampt or NMN.

We first analysed whether visfatin/eNampt could impair endothelium-dependent relaxations *ex vivo* using isolated microvessels from untreated mice, as we had previously described for rat and human mesenteric microvessels^11^. When the microvessels were challenged with 50 ng/mL visfatin/eNampt, the endothelium-independent relaxations to cumulative concentrations of SNP were not affected (Table S2), but the endothelium-dependent responses evoked by ACh were significantly impaired (Fig. 1A).

The *in vivo* effects of visfatin/eNampt (100 ng/kg/day) were explored by its infusion to C57BL/6 mice during 7 days with subcutaneous osmotic mini-pumps. In microvessels isolated at the end of the treatment period, a significant reduction in the endothelium-dependent relaxant responses evoked by ACh was observed in mice receiving visfatin/eNampt (Fig. 1B), while the relaxations elicited by SNP were not affected (Table S3). The 7-day infusion with visfatin/eNampt did not alter the weight, plasma glucose or mean arterial pressure of the animals (Table S1).

Interestingly, the Nampt enzymatic activity inhibitor FK 866 attenuated the defective endothelium-dependent relaxations when co-incubated *ex vivo* (10 µmol/L) or co-infused (2.4 mg/kg/day) during 7 days with visfatin/eNampt (Fig. 1A,B).

### TLR4 activation mediates the endothelial dysfunction induced by visfatin/eNampt both *ex vivo* and *in vivo*

We next aimed to explore whether TLR4 receptors might mediate the defective endothelium-dependent relaxations evoked by visfatin/eNampt. Indeed, the endothelial impairment evoked by visfatin/eNampt was antagonized by the specific TLR4 inhibitor CLI 095, either by *ex vivo* co-incubation (1 µmol/L) or after 7 days of i.p. administration (3 mg/kg/day) (Fig. 1C,D).

We have previously reported that the product of Nampt enzymatic activity NMN mediates visfatin/eNampt-triggered endothelial dysfunction in the rat microvasculature^11^. We found that this was also the case in murine mesenteric microvessels, since NMN (10 µmol/L) mimicked the *ex vivo* effects of visfatin/eNampt (Fig. 1E). Interestingly, the action of NMN was equally antagonized by 1 µmol/L CLI 095 (Fig. 1E).

### Visfatin/eNampt activates the NLRP3 inflammasome in cultured endothelial cells

**Figure 2:**
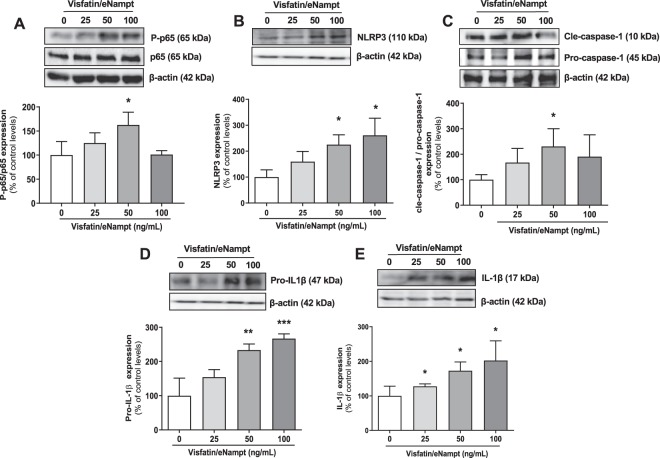
Cultured human umbilical endothelial cells (HUVEC) were exposed to different concentrations of visfatin/eNampt (0, 25, 50, and 100 ng/mL) for 18 h and the following proteins were determined by Western blot. Phosphorylated subunit of p65 (P-p65) and total p65 subunit, taking the ratio of Pp65 to p65 as a measurement of NF-κB activity **(A)**; NLRP3 **(B)**; the ratio of cle-caspase-1 to pro-caspase-1 **(C)**; pro-IL-1β **(D)**; and mature IL-1β **(E)**. Data are expressed as the percentage (mean ± SEM) of the respective protein level in basal conditions. Protein levels were normalized by β-actin levels used as loading control. Every column includes at least 6 different experiments. *p < 0.05 vs respective basal values in the absence of visfatin/eNampt.

**Figure 3:**
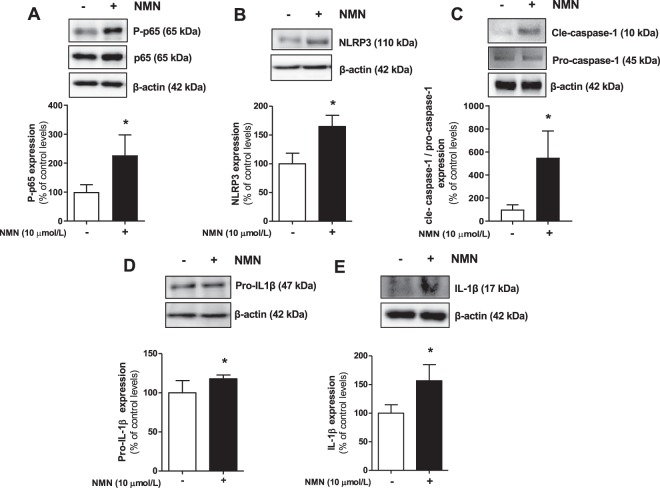
Cultured HUVEC were exposed to different concentrations of 10 μmol/L NMN for 18 h and the following proteins were determined by Western blot: Phosphorylated subunit of p65 (P-p65) and total p65 subunit, taking the ratio Pp65 to p65 as a measurement of NF-κB activity **(A)**; NLRP3 **(B)**; cle-caspase-1 and pro-caspase-1 **(C)**; pro-IL-1β **(D)**; and mature IL-1β **(E)**. Data are expressed as the percentage (mean ± SEM) of the respective protein level in basal conditions. Protein levels were normalized by β-actin levels used as loading control. Every column includes at least 6 different experiments. *p < 0.05 vs respective basal values in the absence of NMN.

We next used a human cell culture model to gain more insight into the cellular mechanisms by which visfatin/eNampt could promote endothelial dysfunction, focusing on pro-inflammatory pathways and NLRP3-inflammasome activation. When cultured HUVEC were exposed to visfatin/eNampt (25 to 100 ng/mL) for 18 h, an enhancement of the ratio between phosphorylated p65 NF-κB subunit (P-p65) and total p65 levels was observed (Fig. 2A), reaching maximum upregulation with 50 ng/ml visfatin/eNampt treatment. Furthermore, the levels of NLRP3 and pro-IL-1β, components of the priming phase of the NLRP3-inflammasome^14,23^, were enhanced in a concentration-dependent manner (Fig. 2B,D). NMN (10 µmol/L) also mimicked the effects of visfatin/eNampt by enhancing P-p65, NLRP3, and pro-IL-1β levels (Fig. 3A,B,D).

**Figure 4:**
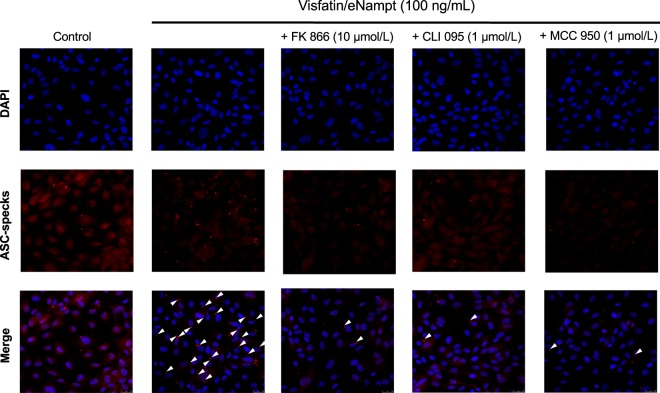
In cultured HUVEC, the presence of the inflammasome related apoptosis-associated speck like protein containing a caspase recruitment domain (ASC-specks) was studied by immunofluorescence, either in control conditions or in the presence of 100 ng/mL visfatin/eNampt and/or 10 µmol/L FK 866, 1 µmol/L CLI 095, and 1 µmol/L MCC 950. Nuclei were counterstained with DAPI (blue) (630×). Arrowheads point ASC-specks aggregates in the different treatments.

**Figure 5:**
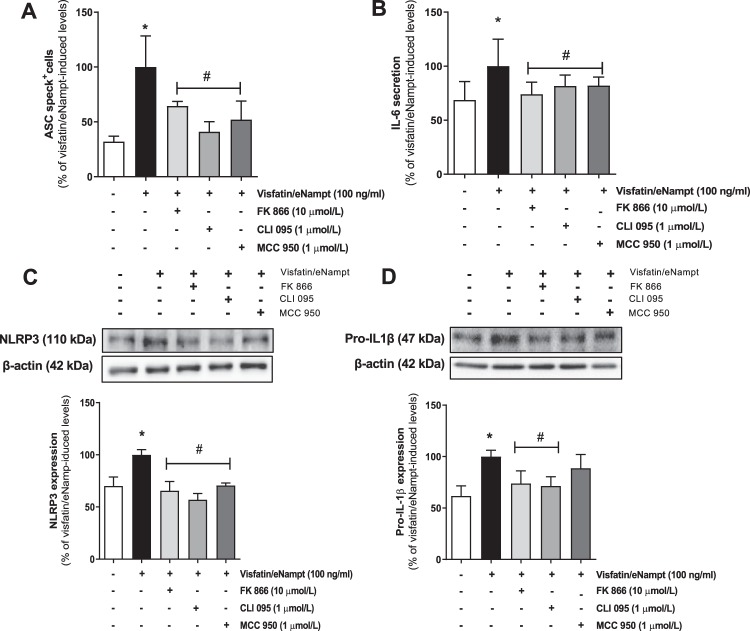
(**A**) ASC-specks present in HUVECs exposed to 100 ng/mL visfatin/eNampt, alone or with 10 µmo/L FK 866, 1 µmol/L CLI 095, or 1 µmol/L MCC 950. **(B)**. ELISA-measured IL-6 levels in the medium from cultured HUVEC submitted to 100 ng/mL visfatin/eNampt, alone or with FK 866, CLI 095 and MCC 950. **(C)** NLRP3 and **(D)** pro-IL-1β levels in cultured HUVEC exposed to 50 ng/mL visfatin/eNampt, alone or with 10 µmol/L FK 866, 1 µmol/L CLI 095, or 1 µmol/L MCC 950. Data (mean ± SEM) are expressed as the percentage of the respective values obtained in the presence of visfatin/eNampt. Protein levels were normalized by β-actin levels used as loading control. Every column includes at least 6 different experiments. *p < 0.05 vs basal; #p < 0.05 vs visfatin/eNampt.

Afterwards, we assessed whether visfatin/eNampt could not only enhance the expression of NLRP3 inflammasome-related proteins but also activate this pro-inflammatory complex. Indeed, both visfatin/eNampt and NMN enhanced the levels of mature IL-1β (Figs. 2E and 3E), as well as significantly increased the expression of the active form of caspase-1, as shown by an increase in the cle-caspase-1 form versus pro-caspase-1 ratio (Figs. 2C and 3C). Moreover, we quantified the formation of apoptosis-associated speck like protein containing a caspase recruitment domain (ASC)-specks, as a readout for inflammasome activation^24^. Visfatin/eNampt (100 ng/mL) enhanced the number of specks in HUVEC cultures (Fig. 4). Importantly, either the blockade of the Nampt activity, the antagonism of TLR4, and the inhibition of the inflammasome assembly using FK 866 (10 µmol/L), CLI 095 (1 µmol/L), or MCC 950 (1 µmol/L), respectively, prevented the increase in ASC-speck number (Figs. 4 and 5A). Furthermore, the measurement of IL-6 in the HUVEC culture medium, as a surrogate marker of IL-1β secretion, showed a significant enhancement in response to visfatin/eNampt, which was also sensitive to FK 866, CLI 095, and MCC 950 (Fig. 5B). These inhibitors also prevented the visfatin/eNampt-induced over-expression of NLRP3 (Fig. 5C), while pro-IL-1β levels were reduced by FK 866 and CLI 095 but not by the inflammasome activation inhibitor MCC 950 (Fig. 5D). Interestingly, CLI 095 (1 µmol/L) was able to prevent the NMN (10 µmol/L)-induced NLRP3 expression in HUVEC as well as completely blunting the enhanced the number of ASC-specks in HUVEC cultures (Fig. S2). In the absence of visfatin/eNampt, there was no change by these drugs in the basal levels of NLRP3 or pro-IL-1β in HUVEC (data not shown).

### Visfatin/eNampt infusion enhances of P-p65 and NLRP3 inflammasome expression in murine aorta and kidney

**Figure 6:**
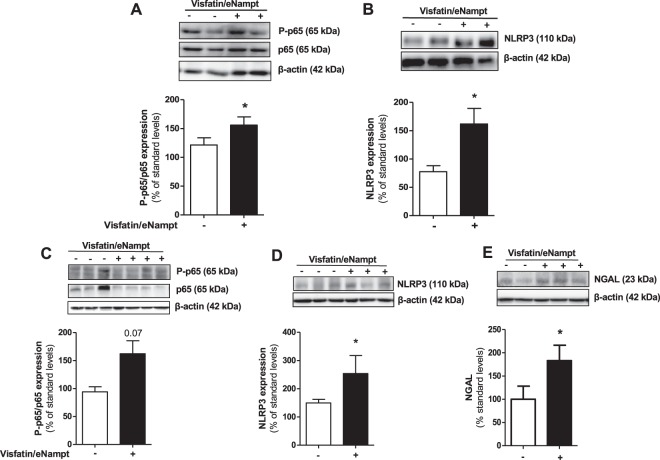
Aortic wall or kidney homogenates from C57BL/6 mice infused for 7 days with saline solution or visfatin/eNampt (100 ng/kg/day) were employed to determine by Western blot the tissular aortic expression of P-p65 and p65 **(A**), NLRP3 **(B)**, as well as the tissular renal levels of P-p65, p65 **(C)**, NLPR3 **(D)**, and neutrophil gelatinase-associated lipocallin (NGAL) **(E)**. Data (mean ± SEM) are expressed as the percentage of the respective protein level obtained for a pool of saline-infused aortic homogenates, termed standard. Protein levels were normalized by β-actin levels used as loading control. Every column includes at least 4 different animals. *p < 0.05 vs standard.

The inflammasome activation were then explored in the aorta from mice infused for 7 days with visfatin/eNampt. The aortic homogenates showed a significant increase of P-p65 levels that was accompanied by enhanced NLRP3 levels suggestive of *in vivo* inflammasome activation in the vascular wall (Fig. 6A,B). Interestingly, p65 subunit phosphorylation and NLRP3 were also stimulated after visfatin/eNampt in kidney extracts (Fig. 6C,D), in a similar fashion than neutrophil gelatinase-associated lipocallin (NGAL), a known marker of renal damage (Fig. 6E). In parallel, visfatin/eNampt induced incipient myocardium and kidney perivascular fibrosis, as a deposition of extracellular matrix around cardiac and renal vessels (Figs. S3 and S4).

### MCC-950 prevents the visfatin/eNampt-induced endothelial dysfunction *in vivo* but not *ex vivo*

**Figure 7:**
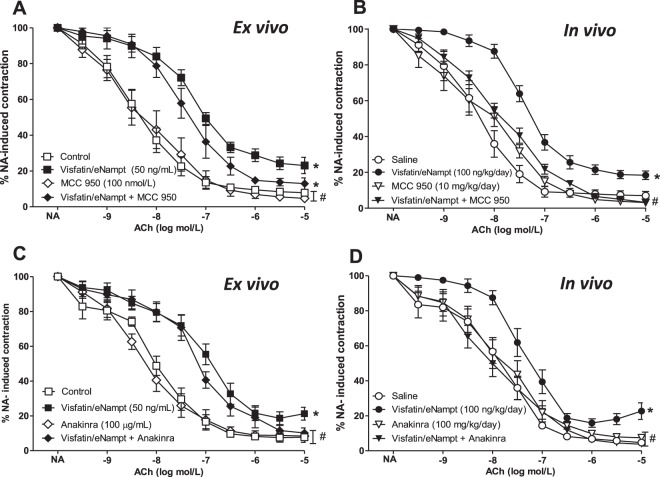
(**A**) Isolated mice mesenteric microvessels obtained from untreated C57BL/6 mice were pre-contracted with 3 μmol/L NA and submitted to cumulative concentrations of the endothelium-dependent vasodilator ACh, (10 nmol/L to 10 μmol/L) in the absence of any previous treatment (Control) or after receiving 50 ng/mL visfatin/eNampt, 100 nmol/L MCC 950, or both visfatin/eNampt plus MCC 950. **(B)** Isolated mice mesenteric microvessels were obtained from C57BL/6 mice infused during 7 days by osmotic mini-pumps with saline solution, visfatin/eNampt (100 ng/kg/day), and receiving also the i.p. administration of MCC 950 (10 mg/kg) or analogous amounts of saline on days 2, 4, and 6. The vessels were pre-contracted with NA and submitted to cumulative concentrations of ACh. **(C)** Isolated mice mesenteric microvessels from untreated C57BL/6 mice submitted to ACh in Control conditions or after receiving 50 ng/mL visfatin/eNampt, 100 µg/mL anakinra, or both visfatin/eNampt plus anakinra. **(D)** Isolated mice mesenteric microvessels obtained from C57BL/6 mice infused with saline solution or visfatin/eNampt for 7 days and receiving also i.p. anakinra (100 mg/kg/day) or analogous amounts of saline during the last 3 days before sacrifice. The curves (mean ± SEM) are expressed as the percentage of the previous NA-evoked contraction, which is indicated in the Tables S1 and S2, as well as the respective pEC_50_ values. For every curve, 5 to 11 segments were used, obtained from 3 to 8 different animals. *p < 0.05 vs respective control or saline. #p < 0.05 vs respective visfatin/eNampt.

After demonstrating that visfatin/eNampt infusion promoted vascular NLPR3 expression *in vivo*, the role for this pro-inflammatory pathway in the development of endothelial dysfunction was investigated. The impairment of endothelium-dependent relaxations acutely evoked *ex vivo* by 50 ng/mL visfatin/eNampt in mesenteric microvessels from untreated mice was not significantly modified by the incubation with the specific NLRP3-inflammasome inhibitor MCC 950 (100 nmol/L; Fig. 7A). However, injection with MCC 950 (i.p., 10 mg/kg) at days 2, 4, and 6 abolished the endothelial dysfunction in mice infused *in vivo* for 7 days with visfatin/eNampt (100 ng/kg/day) (Fig. 7B).

### IL-1 receptor blockade with anakinra antagonizes visfatin/eNampt-induced endothelial dysfunction *in vivo* but not *ex vivo*

Since the NLRP3 inflammasome activation yields IL-1β ^14,23^ we analysed whether the blockade of IL-1 receptors could interfere with visfatin/eNampt-induced endothelial dysfunction. Interestingly, the IL-1R antagonist anakinra displayed differential effects depending on whether visfatin/eNampt-was administered *ex vivo* or *in vivo*. Thus, when isolated mesenteric arteries from untreated mice were incubated *ex vivo* with anakinra (50 ng/mL) there was no changes in the impairment of endothelium-dependent relaxations evoked by visfatin/eNampt (Fig. 7C). On the contrary, the i.p. administration of anakinra (100 µg/mL) at days 4, 5, and 6 to mice infused *in vivo* with visfatin/eNampt prevented the adipokine-induced endothelial dysfunction (Fig. 7D).

To confirm that anakinra was indeed blocking IL-1R, additional experiments were performed using recombinant IL-1β. Both the *ex vivo* incubation of microvascular segments with IL-1β (5 ng/mL) or the infusion of the cytokine (12 µg/kg/day) for 7 days to mice significantly impaired the microvascular endothelium-dependent relaxations to ACh (Fig. S5A,B). These deleterious effects of IL-1β were antagonized by anakinra, when added *ex vivo* to the organ bath or after i.p. injection to the infused mice, respectively (Fig. S5A,B).

## Discussion

There is currently an open discussion about the pathophysiological role of visfatin/eNampt in the development of different cardiometabolic diseases. Indeed, enhanced circulating levels of visfatin/eNampt have been positively associated to type 2 diabetes mellitus (T2DM)^17^ or the metabolic syndrome^25^. Interestingly, an association between enhanced plasma visfatin/eNampt levels and endothelial dysfunction in T2DM patients, measured by forearm plethysmography, was also described^17^. Plasma visfatin/eNampt have also been found elevated in atherosclerotic patients with coronary artery disease^26^ and in type T2DM patients with carotid atherosclerosis^27^.

Indeed, the link between visfatin/eNampt and cardiovascular diseases has been even reinforced by more recent reports^28,29^, both in the presence or in the absence or metabolic alterations^19,30,31^. A common ground of clinical studies linking visfatin/eNampt with cardiovascular diseases highlights the clear relationship between this adipokine and a pro-inflammatory context^32–34^. Moreover, there is a meta-analysis suggesting that visfatin/eNampt may be a promising biomarker not only for predicting obesity, diabetes status, insulin resistance, or metabolic syndrome but also for the development of cardiovascular disease^35^.

Over the last years, different groups, including ours, have proposed that visfatin/eNampt is not only a biomarker but also a direct promotor and mediator of cardiovascular complications^19,20,26^. Regarding this, we have previously demonstrated that visfatin/eNampt induces NF-κB activation, a key driver of pro-inflammatory responses, in cultured vascular smooth muscle cells, this effect being mediated by its enzymatic Nampt activity^9^. Moreover, visfatin/eNampt can also trigger Nampt-dependent endothelial dysfunction when administered *ex vivo* to isolated rat and human mesenteric microvessels or bovine coronary arteries^11,22^. In the present study, we further demonstrated the ability of visfatin/eNampt to promote NF-κB activation and to impair endothelium dependent relaxations, using human endothelial cells or isolated murine mesenteric microvessels, respectively. Similarly to previous reports, a key role for Nampt was acknowledged, since these effects of visfatin/eNampt were blocked by FK 866 and mimicked by NMN, the product of the enzymatic activity, likely involving the release of superoxide anions by NADPH oxidase over-activation^11^.

Although visfatin/eNampt actions were initially suggested to be mediated by the insulin receptor^1^, no specific receptor for this adipokine is currently acknowledged. Our present data indicate that endothelial dysfunction is mediated by TLR4 activation, since the drug CLI 095, which specifically inhibits TLR4-mediated signalling, prevented the defective relaxations induced by visfatin/eNampt. Additionally, TLR4 blockade also prevented NF-κB activation evoked by the adipokine in HUVEC. These findings are consistent with previous reports indicating that TLR4 activation favours NADPH oxidase-mediated production of reactive oxygen species and hence promotes NF-κB activation in HUVEC^36^. A role for TLR4 on the inflammatory responses triggered by visfatin/eNampt has also been suggested in human and mice pulmonary endothelial cells^13^. However, the authors proposed a direct binding between TLR4 and visfatin/eNampt via a protruding region with structural similarity to LPS^13^. In the present study, we observed that exogenously added NMN, the product of Nampt activity, mimicked the effects of visfatin/eNampt in terms of NF-κB activation and both NLRP3 expression and NLRP3 inflammasome activation. Since the action of NMN was also abolished by CLI 095, it cannot be ruled out that NMN itself may be responsible for the activation and subsequent downstream signalling of TLR4 in HUVEC. This hypothesis is also in line with the fact that the inhibitor of Nampt enzymatic activity, FK 866, blunted the actions exerted by visfatin/eNampt.

TLR4 is able to sense both exogenous and endogenous danger signals in order to provide appropriate cellular responses against physiological and environmental signals. In this context, inflammasomes are specialized signal platforms that became evolutionary crucial for the regulation of immune and inflammatory responses^37,38^. Among them, NLRP3 is one of the best characterized sensor proteins of the inflammasomes. Upon activation of certain membrane receptors, including TLR4, an intracellular signalling follows to activate NF-κB and to enhance the transcription of NLRP3 and other proteins, including the pro-form of the inflammatory cytokine IL-1β. In a next step, NLRP3 assemblies with other protein components and conforms an active inflammasome complex capable of activating caspase-1 to catalyze the cleavage of pro-IL-1β into mature IL-1β ^37,38^.

Accordingly with its capacity to interact with TLR4 and to activate NF-κB, both visfatin/eNampt and NMN promoted in HUVEC the expression of NLRP3-inflammasome-related proteins, activating this pro-inflammatory complex and yielding mature pro-inflammatory IL-1β. Therefore, the full canonical NLRP3-inflammasome pathway can be triggered by this adipokine through mechanisms that again rely on its enzymatic Nampt activity. Interestingly, a few reports have proposed visfatin/eNampt as an inflammasome activator in murine endothelial cells, although the mechanisms suggested pointed not at TLR4, but rather towards membrane raft-derived superoxide anions^39^ or receptors for advanced glycosylation end-products^40^.

Based on the data obtained from the functional *ex vivo* studies in isolated microvessels and from the *in vitro* mechanistic findings in HUVEC, we designed an experimental approach to assess the effects of *in vivo* visfatin/eNampt infusion in healthy mice. The aim was to clarify, in the absence of any other pathophysiological condition, whether visfatin/eNampt was capable to induce by itself endothelial dysfunction and vascular inflammation in a more complex environment and after a sustained non-acute infusion. Using this *in vivo* approach, visfatin/eNampt was again proved as an inductor of endothelial dysfunction. As for *ex vivo* or *in vitro* studies, the Nampt activity and the activation of TLR4 receptors arose as key mediators of the deleterious action of visfatin/eNampt *in vivo*.

Besides inducing defective endothelium-dependent relaxations, visfatin/eNampt promoted tissue inflammation both at vascular and renal levels, as indicated by the activation of NF-κB and the overexpression of NLRP3 protein. In renal homogenates from visfatin/eNampt, the enhanced protein levels of NGAL, which is also involved in innate immunity, were suggestive of early renal damage. In fact, NGAL itself has been proposed to activate the NLRP3 inflammasome^41^, perhaps amplifying the inflammatory signal originated by visfatin/eNampt. Although incipiently, renal and cardiac tissue also displayed early histological signs of perivascular fibrosis.

Taken together, these findings pointed at NLRP3 inflammasome activation as a major mechanism mediating the endothelial dysfunction and defective relaxation evoked by visfatin/eNampt infusion *in vivo*. Accordingly, the specific NLRP3 inhibitor MCC 950^42^ was able to prevent such dysfunction. This might be in line with other reports identifying the NLRP3 inflammasome as a main effector of the murine vascular damage evoked by visfatin/eNampt, leading to enhanced *neointima* formation^39^ or inter-endothelial junctions disruption^40^.

Interestingly, the NLRP3 inflammasome inhibitor was ineffective for preventing the defective relaxation acutely evoked by visfatin/eNampt *ex vivo*. It is most possible that a shorter time after stimulation did not allow inflammasome priming and activation. In fact, this observation unveils that the activation of TLR4 by visfatin/eNampt or NMN may trigger differential and time-dependent signals to ultimately provoke endothelial dysfunction. While long-term vascular effects of the adipokine appear to be NLRP3-inflammasome dependent, the short-term defective relaxation relies on NLRP3-indpendent signals. In fact, we have previously demonstrated that visfatin/eNampt acutely generates reactive oxygen species by NADPH oxidase activation and that NADPH oxidase inhibitors may prevent the acute defective relaxation evoked by the adipokine *ex vivo*^11^.

Globally, these findings support a growing evidence suggesting that the activation of the NLRP3 inflammasome actively contributes to the inflammatory response that drives cardiovascular diseases, particularly when associated to metabolic alterations^43,44^. Moreover, our data suggest that endothelial cells can be a very early interface for interaction between adipokines like visfatin/eNampt and the vascular wall inflammation. In this context, selective drug inhibitors of the NLRP3 assembly, like MCC 950, arise as promising therapeutic alternatives for treating cardiovascular diseases^45,46^.

Since NLRP3 inflammasome activation yields mature IL-1β, we hypothesized that the cytokine itself could be the final effector mediating the chronic endothelial dysfunction induced by visfatin/eNampt. Interestingly, the IL-receptor antagonist anakinra prevented the defective relaxation evoked by the *in vivo* infusion, confirming IL-1β as a key mediator of endothelial dysfunction induced by visfatin/eNampt. Moreover, as for the NLRP3 inflammasome inhibition, anakinra had no effect on the acute *ex vivo* effects exerted by visfatin/eNampt, further supporting the existence of both a rapid NLRP3-independent and a long-term NLRP3-dependent mechanism of endothelial dysfunction.

In line with these findings, we have previously demonstrated the capacity of exogenously added *ex vivo* IL-1β to evoke endothelial dysfunction in rats by a mechanism involving IL-1 receptors and NADPH oxidase-derived superoxide anions^47^. Here, we demonstrate a deleterious action of the cytokine on mice microvessels, not only acutely *ex vivo* but also after *in vivo* administration, all these effects being effectively prevented by anakinra. In fact, anakinra has been shown to improve endothelial dysfunction in a rat model of diabetes, in which the circulating levels of pro-inflammatory cytokines, including IL-1β, were not augmented^47^. This supports a paracrine vascular production of IL1β, consistent with a local NLRP3 inflammasome activation, playing a major role in vascular dysfunction. Moreover, the locally released IL-1β may in turn favour the secretion of visfatin/eNampt from endothelial cells^7^ therefore maintaining and amplifying vascular inflammation.

From a therapeutic point of view, the blockade of IL-1 receptors becomes a valuable strategy to antagonize those vascular inflammatory responses elicited by many different stimuli, including vistatin/eNampt, that ultimately converge in vascular NLRP3-inflammasome activation and IL-1β production. Indeed, this might explain why IL-receptor antagonists produce important benefits in human and experimental models of cardiometabolic diseases by reducing the systemic, and perhaps the local, pro-inflammatory environment^11,48–50^. Analogously, this may explain the results of the recent clinical trial CANTOS using canakinumab, a monoclonal antibody against IL-1β^51^. This study enrolled 10,061 patients with previous myocardial infarction and elevated levels of high-sensitivity C-reactive protein (hs-CRP)^52^. In this seminal trial, four years of canakinumab treatment significantly decreased cardiovascular events in this high risk population, independently of plasmatic lipid reduction^52^ but in correlation with a reduction in plasma hs-CRP levels^53^. In fact, hs-CRP has been proposed as a surrogate circulating marker of the upstream biological tissue activity of IL-1β. Interestingly, although those patients with diabetes mellitus or chronic kidney disease benefited from canakinumab treatment from a cardiovascular point of view, the evolution of their respective metabolic or kidney disease was not modified^54,55^, thus proving a quite selective effect of this anti-inflammatory drug at vascular level rather than at metabolic or renal alterations.

## Conclusion

Visfatin/eNampt produces *in vivo* endothelial dysfunction and tissue inflammation. This supports a role for this pleiotropic adipokine as a mediator of vascular damage in those clinical conditions in which its levels are elevated, such as diabetes mellitus and obesity. The deleterious effects of visfatin/eNampt implicate not only Nampt enzymatic activity and TLR4 activation, but the activation of intracellular inflammatory signals ultimately leading to NLRP3 stimulation and the secretion of mature IL-1β. In this clinical context, the pharmacological blockade of TLR4 or the NLRP3/IL-1β/IL-1 receptor axis may provide a therapeutic strategy for preventing and/or treating the vascular alterations associated to metabolic diseases.

## Methods

### Materials

Visfatin/eNampt and IL-1β were obtained from PeproTech (PeproTech GmbH, Hamburg, Germany) while anakinra was obtained from Biovitrum (Swedish Orphan Biovitrum AB, Stockholm, Sweden). FK866, NA, KCl, ACh, SNP, and, unless otherwise stated, all other reagents were purchased from Sigma Chemical Co. (St. Louis, MO, USA). CLI095 was from Invitrogen (Carlsbad, CA, USA). Culture plastic ware was from TPP (Trasadingen, Switzerland). M199 medium, foetal calf serum (FCS) and trypsin-EDTA were from Biological Industries (Beit-Hamek, Israel). The composition of KHS (mmol/L) was NaCl 115, CaCl_2_ 2.5, KCl 4.6, KH_2_PO_4_ 1.2, MgSO_4_.7H_2_O 1.2, NaHCO_3_ 25, glucose 11.1 and Na_2_EDTA 0.03. Noradrenaline was prepared in saline solution (0.9% NaCl)-ascorbic acid (0.01% w/v). All other drug solutions were made in distilled water.

### Animals and experimental groups

4 month-old male C57BL/6 mice were maintained under standardized conditions with an artificial 12 h-12 h dark-light cycle, with *ad libitum* access to food and water. All animal studies were performed in accordance with National and European guidelines and regulations (RD 53/2013; Directive 2010/63/EU) and were approved by the institutional animal care (CEI-59-1052-A062; PROEX 026/15).

Age-matched male mice were randomly allocated in the experimental groups, as indicated in Fig. S1. Briefly, osmotic mini-pumps (Alzet, model 1007D, DURECT Corporation, Cupertino, CA, USA) were implanted in the animals for 7 days, infusing either saline vehicle (NaCl 0.9%), visfatin/eNampt (100 ng/kg/day), FK 866 (2.4 mg/kg/day), both visfatin/eNampt plus FK 866, or interleukin-(IL)-1β (12 µg/kg/day). The mini-pumps contained a volume of 100 µL and a constant infusion speed of 0,5 µL/hour was established. Then, the minipumps were implanted subcutaneously under the scapule in animals previously anesthetized with i.p. injection of 50 µL Imalgene^@^ (ketamine, 50 mg/mL) and 10 µL de xilacine (Xilagesic 2%) solved in 0.5 mL NaCl (0.9%). Some of the mice also received i.p the inhibitors CLI 095 (3 mg/kg/day), MCC 950 (10 mg/kg on days 2, 4, and 6) or the IL-1 antagonist anakinra (100 mg/kg on days 4, 5, and 6).

Additionally, a group of control untreated animals were used for analysing the *ex vivo* effects of the drugs. To sacrifice, the animals were briefly exposed to a chamber filled with carbon dioxide until they fell unconscious and then immediately killed by cervical dislocation. On day 0 and 6, weight, plasma glucose, and mean arterial pressure were measured, leading to minor changes: a small weight reduction was observed in CLI 095-treated mice while significant lower plasma glucose levels were obtained in IL-1β-treated animals (Table S1).

### Biochemical data

Blood plasma samples were collected by venipuncture in a BD Vacutainer® with heparin (BD, Franklin Lakes, NJ, USA) from cava vein and stored at **−**80 °C until assay was performed. Serum was prepared according to the manufacturer’s recommendations of inversion and centrifugation and aliquoted and stored at −80 °C until use in experiments.

### Drug effects on vascular tone of mesenteric microvessels

For reactivity experiments, the mesentery was removed, placed in a Petri dish containing Krebs-Henseleit solution (KHS) at 4 °C. The third branch mesenteric arteries were dissected (mean internal diameter ranged between 150–400 µm, with non-significant differences observed among the different groups of mice). The arteries were dissected cleaned free of fat and connective tissue under a light microscope and mounted as ring preparations on a small vessel myograph^11^ capable of measuring isometric tension. Arteries were bathed in KHS at 37 °C continuously bubbled with a 95% O_2_-5% CO_2_ mixture, which gives a pH of 7.4 and their passive tension and internal circumference were determined. The arteries were subjected to optimal tension (90% of the tension equivalent to a intramural pressure of 100 mm Hg. After 30 min of equilibration, the vessels were exposed to 125 mmol/L K + (KKHS, equimolar substitution of KCl for NaCl in KHS) for 2 min to check their functional integrity. Segments failing to produce a maximum active tension equivalent to a pressure of 100 mmHg on the final contraction were rejected^11^.

The bath was then washed three times with KHS and further 60–120 min washout period was allowed before the arteries were contracted with the concentration of noradrenaline (NA; 3 µmol/L) required to produce approximately 80% of the maximum response to KKHS. Endothelium-dependent relaxations to acetylcholine (ACh) were subsequently assessed by adding cumulative concentrations of the drug at 2 min intervals (final bath concentrations 10 nmol/L to 10 µmol/L). In some experiments, instead of ACh, concentration-dependent curves to sodium nitroprusside (SNP; 1 nmol/L to 100 µmol/L) were performed to determine endothelium-independent relaxations. Moreover, in other experiments, mesenteric microvessels were incubated in the organ chamber (*ex vivo*) with visfatin/eNampt (50 ng/mL), nicotinamide mononucleotide (NMN; 10 µmol/L), and/or FK 866 (10 µmol/L), CLI 095 (1 µmol/L), or MCC 950 (100 nmol/L), 30 min in advance and during the administration of NA, ACh or SNP. In other set of experiments, the microvessels were treated with IL-1β (5 ng/mL) and/or anakinra (100 µg/mL), 120 min in advance and during the administration of NA, ACh, or SNP.

### Cell culture

Human umbilical vein endothelial cells (HUVEC) were isolated from umbilical cords, as previously described^7^. Cells were cultured in M199 medium supplemented with 20% foetal calf serum (FCS), 25 μg/mL endothelial cell growth supplement (ECGS), 100 μg/mL heparin and antibiotics (100 U/ml penicillin, 100 µg/ml streptomycin and 2.5 µg/ml amphotericin B) at 37 °C in a humidified atmosphere with 5% CO2. Cells at passages 1–5 were incubated for the indicated time periods with the different test compounds in M199 medium supplemented with 10% FCS, ECGS and antibiotics. All the procedures were reviewed and approved by the ethics committee of Universidad Autónoma of Madrid and Hospital Universitario La Paz, respectively, and written informed consent was obtained from all cord donors.

### Western blot analysis

At the end of the treatment periods, the levels of selected proteins were detected by Western blot in HUVEC or in aortic and renal homogenates, as previously described^7^. Primary antibodies were used against phospho-p65 (P-p65), total p65, NLRP3, pro-caspase-1 and cleaved(cle)-caspase-1 forms, IL-1β, or pro-IL-1β (Cell Signalling, Adipogen; Novus Biologicals; R&D systems, respectively, at 1/1,000 dilution) and anti β-actin primary antibody (dilution 1/10,000; Sigma-Aldrich) to ensure equal loading, followed by incubation with corresponding specific horseradish peroxidase-conjugated secondary antibodies (Bio-Rad or Bethyl; 1:10,000) Immunoreactive bands were detected using an ECL detection kit (GE Healthcare) and quantified by densitometry using the NIH software Image J.

### Indirect immunofluorescence

The activation of the inflammasome requires ASC proteins to assemble into a large toroidal protein complex, which is termed “speck”^24^. ASC-specks were visualized in HUVEC by indirect immunofluorescence as previously described^7^. A primary polyclonal antibody against ASC (dilution 1:250; Molecular Probes) was used, followed by incubation with an appropriate Alexa 546-conjugated secondary antibody (dilution 1:100; Molecular Probes). Nuclei were counterstained with DAPI (5 µg/mL, Invitrogen) and cells were observed with a confocal microscopy (TCS SPE, Leica, Wetzlar, Germany). The percentage of specks was calculated as the percentage of cells displaying specks versus the total number of cell per field. Specks were first counted manually with a fluorescence microscope (Eclipse TE300; Nikon, Tokyo, Japan). In every preparation, we initially selected a central field and afterwards, following a counterclock radial pattern, eighth radius were traced and two fields were explored in each of them (total measurements, 17 fields per preparation at 100×). Representative images (63×) were obtained from every preparation with a confocal microscope (TCS SPE, Leica, Wetzlar, Germany).

### IL-6 secretion

After the appropriate treatments and incubation times, supernatants were collected, centrifuged at 1.500 rpm for 10 min at 4 °C and frozen at −20 °C until further use. IL-6 was measured with an ELISA immunoassay (Raybiotech, Norcross, GA, USA) according to the manufacturer instructions.

### Histological studies

Serial paraffin sections (4 μm) of half-height sliced myocardium or kidneys were fixed on slides and stained with Haematoxylin/Eosin (H/E). In parallel, Masson trichrome was used to detect extra-cellular matrix (ECM) deposition by sequent addition of Bouin’s, Weigert’s and Biebrich solutions (Bio-Optica, Milan, Italy) on paraffin sections (4 μm) of all myocardia and kidney samples. Interstitial, perivascular and replacement fibrosis were quantified together on five fields of each organ preparation using the Metamorph software. Photographs were taken at 40x magnification under optical microscopy (Eclipse TE300; Nikon, Tokyo, Japan).

### Statistical analysis

Results are expressed as mean ± standard error (SEM). pEC_50_ values for ACh were defined as the negative log of the effective dose required to produce half the maximum effect. Statistical analysis was performed using t student test and one-way ANOVA followed by Bonferroni *post hoc* test, or two-way ANOVA, as appropriate. A *p* value ≤ 0.05 was considered statistically significant.

### Ethics approval and consent to participate

All animal studies followed National and European guidelines (RD 53/2013; Directive 2010/63/EU) and were approved by the institutional animal care (CEI-59-1052-A062; PROEX 026/15). In the HUVEC studies, the procedures were reviewed and approved by the ethics committee of Universidad Autónoma of Madrid and Hospital Universitario La Paz, respectively, and written informed consent was obtained from all cord donors.

## Supplementary information

Supplementary information.

## Data Availability

The datasets used and/or analysed during the current study are available from the corresponding author on reasonable request.
