# Supplementary material for: Visfatin/eNampt induces endothelial dysfunction in vivo: a role for Toll-Like Receptor 4 and NLRP3 inflammasome
Source: Sci Rep. 2020 Mar 25;10:5386. doi: 10.1038/s41598-020-62190-w (PMC7096459; doi:10.1038/s41598-020-62190-w)
Supplement: Supplementary file 1 — Supplementary information. [file 41598_2020_62190_MOESM1_ESM.pdf]

Visfatin/eNampt induces endothelial dysfunction *in vivo*: a role for Toll-Like Receptor 4 and NLRP3 inflammasome

Tania Romacho[1,2]*, Inés Valencia[1,2,3]*, Mariella Ramos-González[1,2,3]*, Susana Vallejo[1,2], Miguel López-Esteban[1], Oscar Lorenzo[4,5], Pablo Cannata[4,5], Alejandra Romero[1,2,3], Alvaro San-Hipólito[1,2,3], Jorge F. Gomez-Cerezo[6,2], Concepción Peiró[1,2]#, Carlos F. Sánchez-Ferrer[1,2]#

*These authors contributed equally to this work

#Both authors jointly directed this work

[1]Departamento de Farmacología y Terapéutica, Facultad de Medicina, Universidad Autónoma de Madrid, Spain

[2]Instituto de Investigación Sanitaria del Hospital Universitario La Paz (IdiPAZ), Madrid, Spain.

[3]PhD Programme in Pharmacology and Physiology, Doctoral School, Universidad Autónoma de Madrid.

[4]Departamento de Medicina, Facultad de Medicina, Universidad Autónoma de Madrid, Spain

[5]Instituto de Investigación Sanitaria Fundación Jiménez Díaz, Madrid, Spain.

[6]Servicio de Medicina Interna, Hospital Universitario Infanta Sofía, Madrid, Spain

Running headline (máx 50 characters): NLRP3 inflammasome on vascular damage by visfatin/eNampt

Correspondence: Carlos F. Sánchez-Ferrer (carlosf.sanchezferrer@uam.es), or Concepción Peiró (concha.peiro@uam.es), Departamento de Farmacología, Facultad de Medicina, Universidad Autónoma de Madrid, Calle Arzobispo Morcillo 4, 28029-Madrid, Spain. Phone: +34914975470, Fax: +349149753580. Full list of author information is available at the end of the article.

SUPPLEMENTARY INFORMATION

Supplementary methods

Materials

Visfatin/eNampt and IL-1β were obtained from PeproTech (PeproTech GmbH, Hamburg, Germany) while anakinra was obtained from Biovitrum (Swedish Orphan Biovitrum AB, Stockholm, Sweden). FK866, NA, KCl, ACh, SNP, and, unless otherwise stated, all other reagents were purchased from Sigma Chemical Co. (St. Louis, MO, USA). CLI095 was from Invitrogen (Carlsbad, CA, USA). Culture plastic ware was from TPP (Trasadingen, Switzerland). M199 medium, foetal calf serum (FCS) and trypsin-EDTA were from Biological Industries (Beit-Hamek, Israel). The composition of KHS (mmol/L) was NaCl 115, $CaCl_2$ 2.5, KCl 4.6, KH_2PO_4 1.2, $MgSO_4 \cdot 7H_2O$ 1.2, $NaHCO_3$ 25, glucose 11.1 and Na_2EDTA 0.03. Noradrenaline was prepared in saline solution (0.9% NaCl)-ascorbic acid (0.01% w/v). All other drug solutions were made in distilled water.

Animals and experimental groups

4 month-old male C57BL/6 mice were maintained under standardized conditions with an artificial 12 h-12 h dark-light cycle, with *ad libitum* access to food and water. All animal studies were performed in accordance with National and European guidelines and regulations (RD 53/2013; Directive 2010/63/EU) and were approved by the institutional animal care (CEI-59-1052-A062; PROEX 026/15).

Age-matched male mice were randomly allocated in the experimental groups, as indicated in Figure S1. Briefly, osmotic mini-pumps (Alzet, model 1007D, DURECT Corporation, Cupertino, CA, USA) were implanted in the animals for 7 days, infusing either saline vehicle (NaCl 0.9%), visfatin/eNampt (100 ng/kg/day), FK 866 (2.4 mg/kg/day), both visfatin/eNampt plus FK 866, or interleukin-(IL)-1β (12 µg/kg/day). The mini-pumps contained a volume of 100 µL and a constant infusion speed of 0,5 µL/hour was established. Then, the minipumps were implanted subcutaneously under the scapule in animals previously anesthetized with i.p. injection of 50 µL Imalgene@ (ketamine, 50 mg/mL) and 10 µL de xilacine (Xilagesic 2%) solved in 0.5 mL NaCl (0.9%). Some of the mice also received i.p the inhibitors CLI 095 (3 mg/kg/day), MCC 950 (10 mg/kg on days 2, 4, and 6) or the IL-1 antagonist anakinra (100 mg/kg on days 4, 5, and 6).

Additionally, a group of control untreated animals were used for analysing the *ex vivo* effects of the drugs. To sacrifice, the animals were briefly exposed to a chamber filled with carbon dioxide until they fell unconscious and then immediately killed by cervical dislocation. On day 0 and 6, weight, plasma glucose, and mean arterial pressure were measured, leading to minor changes: a small weight reduction was observed in CLI 095-treated mice while significant lower plasma glucose levels were obtained in IL-1β-treated animals (Table S1).

Biochemical data

Blood plasma samples were collected by venipuncture in a BD Vacutainer® with heparin (BD, Franklin Lakes, NJ, USA) from cava vein and stored at − 80°C until assay was performed. Serum was prepared according to the manufacturer's recommendations of inversion and centrifugation and aliquoted and stored at -80°C until use in experiments.

Drug effects on vascular tone of mesenteric microvessels

For reactivity experiments, the mesentery was removed, placed in a Petri dish containing Krebs-Henseleit solution (KHS) at 4°C. The third branch mesenteric arteries were dissected (mean internal diameter ranged between 150-400 µm, with non-significant differences observed among the different groups of mice). The arteries were dissected cleaned free of fat and connective tissue under a light microscope and mounted as ring preparations on a small vessel myograph [11] capable of measuring isometric tension. Arteries were bathed in KHS at 37°C continuously bubbled with a 95% O_2-5% CO_2 mixture, which gives a pH of 7.4 and their passive tension and internal circumference were determined. The arteries were subjected to optimal tension (90% of the tension equivalent to a intramural pressure of 100 mm Hg. After 30 min of equilibration, the vessels were exposed to 125 mmol/L K^+ (KKHS, equimolar substitution of KCl for NaCl in KHS) for 2 min to check their functional integrity. Segments failing to produce a maximum active tension equivalent to a pressure of 100 mmHg on the final contraction were rejected [11].

The bath was then washed three times with KHS and further 60-120 min washout period was allowed before the arteries were contracted with the concentration of noradrenaline (NA; 3 µmol/L) required to produce approximately 80% of the maximum response to KKHS. Endothelium-dependent relaxations to acetylcholine (ACh) were subsequently assessed by

adding cumulative concentrations of the drug at 2 min intervals (final bath concentrations 10 nmol/L to 10 µmol/L). In some experiments, instead of ACh, concentration-dependent curves to sodium nitroprusside (SNP; 1 nmol/L to 100 µmol/L) were performed to determine endothelium-independent relaxations. Moreover, in other experiments, mesenteric microvessels were incubated in the organ chamber (*ex vivo)* with visfatin/eNampt (50 ng/mL), nicotinamide mononucleotide (NMN; 10 µmol/L), and/or FK 866 (10 µmol/L), CLI 095 (1 µmol/L), or MCC 950 (100 nmol/L), 30 min in advance and during the administration of NA, ACh or SNP. In other set of experiments, the microvessels were treated with IL-1β (5 ng/mL) and/or anakinra (100 µg/mL), 120 min in advance and during the administration of NA, ACh, or SNP.

Cell Culture

Human umbilical vein endothelial cells (HUVEC) were isolated from umbilical cords, as previously described [7]. Cells were cultured in M199 medium supplemented with 20% foetal calf serum (FCS), 25 µg/mL endothelial cell growth supplement (ECGS), 100 µg/mL heparin and antibiotics (100 U/ml penicillin, 100 µg/ml streptomycin and 2.5 µg/ml amphotericin B) at 37°C in a humidified atmosphere with 5% CO2. Cells at passages 1-5 were incubated for the indicated time periods with the different test compounds in M199 medium supplemented with 10% FCS, ECGS and antibiotics. All the procedures were reviewed and approved by the ethics committee of Universidad Autónoma of Madrid and Hospital Universitario La Paz, respectively, and written informed consent was obtained from all cord donors.

Western blot analysis

At the end of the treatment periods, the levels of selected proteins were detected by Western blot in HUVEC or in aortic and renal homogenates, as previously described [7]. Primary antibodies were used against phospho-p65 (P-p65), total p65, NLRP3, pro-caspase-1 and cleaved(cle)-caspase-1 forms, IL-1β, or pro-IL-1β (Cell Signalling, Adipogen; Novus Biologicals; R&D systems, respectively, at 1/1,000 dilution) and anti β-actin primary antibody (dilution 1/10,000; Sigma-Aldrich) to ensure equal loading, followed by incubation with corresponding specific horseradish peroxidase-conjugated secondary antibodies (Bio-Rad or Bethyl; 1:10,000) Immunoreactive bands were detected using an ECL detection kit (GE Healthcare) and quantified by densitometry

using the NIH software Image J.

Indirect immunofluorescence

The activation of the inflammasome requires ASC proteins to assemble into a large toroidal protein complex, which is termed "speck" [23]. ASC-specks were visualized in HUVEC by indirect immunofluorescence as previously described [7]. A primary polyclonal antibody against ASC (dilution 1:250; Molecular Probes) was used, followed by incubation with an appropriate Alexa 546-conjugated secondary antibody (dilution 1:100; Molecular Probes). Nuclei were counterstained with DAPI (5 µg/mL, Invitrogen) and cells were observed with a confocal microscopy (TCS SPE, Leica, Wetzlar, Germany). The percentage of specks was calculated as the percentage of cells displaying specks versus the total number of cell per field. Specks were first counted manually with a fluorescence microscope (Eclipse TE300; Nikon, Tokyo, Japan). In every preparation, we initially selected a central field and afterwards, following a counterclock radial pattern, eighth radius were traced and two fields were explored in each of them (total measurements, 17 fields per preparation at 100X). Representative images (63X) were obtained from every preparation with a confocal microscope (TCS SPE, Leica, Wetzlar, Germany).

Histological studies

Serial paraffin sections (4 µm) of half-height sliced myocardium or kidneys were fixed on slides and stained with Haematoxylin/Eosin (H/E). In parallel, Masson trichrome was used to detect extra-cellular matrix (ECM) deposition by sequent addition of Bouin's, Weigert's and Biebrich solutions (Bio-Optica, Milan, Italy) on paraffin sections (4 µm) of all myocardia and kidney samples. Interstitial, perivascular and replacement fibrosis were quantified together on five fields of each organ preparation using the Metamorph software. Photographs were taken at 40x magnification under optical microscopy (Eclipse TE300; Nikon, Tokyo, Japan).

Supplementary legends

Additional file 1: Table S1. Vascular tone achieved with noradrenaline prior to relaxation curves and pEC_{50} values for ACh or SNP in isolated mesenteric microvessels from C57BL/6 mice infused for 7 days with visfatin/eNampt or IL-1β.

Additional file 2: Table S2. Weight, plasmatic glucose, and mean arterial pressure in C57BL/6 mice infused for 7 days by osmotic mini-pumps.

Additional file 3: Table S3. Vascular tone achieved with noradrenaline prior to relaxation curves and pEC_{50} values for ACh or SNP in isolated mesenteric microvessels from untreated C57BL/6 mice.

Additional file 4: Figure S1. Schematic representation of the designed experimental groups of C57BL/6 mice infused with visfatin/eNampt or IL-1β through subcutaneously implanted osmotic minipumps and the specified durgs.

Additional file 5: Figure S2. In cultured HUVEC, both the NLRP3 expression and the presence of the inflammasome related apoptosis-associated speck like protein containing a caspase recruitment domain (ASC-specks) were studied by Western blot and immunofluorescence, respectively, either in control conditions or in the presence of 10 µmol/L NMN and/or 1 µmol/L CLI 095 and 1 µmol/L MCC 950. **(A)** NLRP3 expression quantification and representative gels. NLRP3 levels were normalized by β-actin as loading control. **(B)** Quantitative ASC-speck analysis and **(C)** representative pictures with nuclei counterstained with DAPI (blue) (630x). Arrowheads point ASC-speck aggregates in the different treatments. Data (mean±SEM) are expressed as the percentage of the respective values obtained in the presence of NMN. Every column includes at least 3 different experiments. *$p<0.05$ vs basal; #$p<0.05$ vs NMN.

Additional file 6: Figure S3. Profibrotic effects in hearts from C57BL/6 mice infused for 7 days with visfatin/eNampt. Arrows indicate, by Masson staining, pictures and quantitative analysis of interstitial and perivascular ECM protein deposition in saline- and visfatin/eNampt-infused mice. (400x).

Additional file 6: Figure S4. Profibrotic effects in kidneys from C57BL/6 mice infused for 7 days with visfatin/eNampt. Arrows indicate, by Masson staining, interstitial and perivascular ECM protein deposition in saline- and visfatin/eNampt infused mice. (400x).

Additional file 7: Figure S5. (A) Isolated mice mesenteric microvessels obtained from untreated C57BL/6 mice were pre-contracted with 3 µmol/L noradrenaline (NA) and submitted to cumulative concentrations of ACh (10 nmol/L to 10 µmol/L) in the absence of any previous treatment (Control)

or after receiving 5 ng/mL IL-1β, 100 µg/mL anakinra, or both IL-1β plus anakinra. **(B)** Isolated mice mesenteric microvessels were obtained from animals infused during 7 days, through subcutaneous osmotic mini-pumps, with saline solution or IL-1β (12 µg/kg/day), and receiving also intraperitoneal administration of anakinra (100 mg/kg/day) or analogous amounts of saline during the last 3 days before sacrifice. The isolated vessels were pre-contracted with NA and submitted to cumulative concentrations of ACh. The curves (mean±SEM) are expressed as the percentage of the previous NA-evoked contraction, which is indicated in the Tables S2 and S3, as well as the respective pEC_{50} values. For every curve, 7 to 11 segments were used, obtained from 4 to 8 different animals. *$p<0.05$ vs respective control. #$p<0.05$ vs respective IL-1β.

Table S1. Weight, plasmatic glucose, and mean arterial pressure in C57BL/6 mice infused for 7 days by osmotic mini-pumps

Treatment	Weight (g)		Plasmatic glucose (mmol/L)		Mean arterial pressure (mm Hg)	
	Day 0	Day 6	Day 0	Day 6	Day 0	Day 6
Saline solution (12 µL/day)	28.97±0.49	27.70±1.82	8.22±0.45	6.81±0.40	102.67±5.32	104.52±2.24
Visfatin/eNampt (100 ng/kg/day)	29.31±0.65	28.97±0.49	7.94±1.17	7.72±0.53	98.80±2.12	101.13±2.19
FK 866 (2.4 mg/kg/day)	28.45±1.41	29.90±1.21	7.62±0.45	8.20±0.54	102.53±3.10	93.52±5.31
CLI 095 (3 mg/kg[a])	28.70±0.67	26.10±0.72*	5.98±0.16	6.09±0.12	97.84±3.71	102.72±2,18
MCC 950 (10 mg/kg[a])	24.75±0.84	24.38±0.86	6.63±0.51	6.99±0.60	100.96±1.58	100.96±1.58
Anakinra (100 mg/kg[a])	29.56±0.66	30.65±0.91	7.76±0.98	6.64±0.17	99.49±2.97	101.08±5.86
Visfatin/eNampt (100 ng/kg/day) + FK 866 (2.4 mg/kg/day)	29.40±0.77	30.02±0.62	7.72±0.59	7.22±0.59	100.64±11.57	93.45±3.62
Visfatin/eNampt (100 ng/kg/day) + CLI 095 (3 mg/kg[a])	29.68±0.23	27.90±0.65+	7.35±1.46	7.60±1.09	105.30±5.86	103.09±3.61
Visfatin/eNampt (100 ng/kg/day) + MCC 950 (10 mg/kg[a])	27.03±0.65	26.68±0.50	6.48±0.47	7.20±0.49	111.20±5.57	103.22±2.70
Visfatin/eNampt (100 ng/kg/day) + Anakinra (100 mg/kg[a])	31.00±0.40	30.80±0.20	7.55±0.27	8.74±0.86	114.90±0.93	101.35±5.41
IL-1β (12 µg/kg/day)	29.40±0.72	27.70±8.00	6.66±0.28	4.56±0.42*	97.52±2.73	102.18±2.42
IL-1β (12 µg/kg/day) + Anakinra (100 mg/kg[a])	30.67±7.67	31.48±0.56	7.06±0.71	6.76±0.80	108.25±6.23	95.51±6.23

Animals were infused with either saline solution, visfatin/eNampt, FK 866, visfatin/eNampt plus FK 866, or IL-1β. [a]These animals received i.p. CLI 095 (days 1 to 6), MCC 950 (days 2, 4, and 6), or anakinra (days 4, 5, and 6) before sacrifice. Determinations were obtained on days 0 and 6. Results are expressed as mean ± SEM. *p<0.05 vs saline solution; +p<0.5 vs respective visfatin/eNampt.

Table S2. Vascular tone achieved with noradrenaline prior to relaxation curves and pEC_{50} values for ACh or SNP in isolated mesenteric microvessels from untreated C57BL/6 mice

Ex vivo drug administered	Tone prior to relaxation (mN)	pEC_{50} for ACh
None (control)	6.12 ± 0.22	7.91 ± 0.07
Visfatin/eNampt (50 ng/mL)	5.40 ± 0.27	6.92 ± 0.22*
NMN (10 µmol/L)	6.17 ± 0.51	7.16 ± 0.18*
FK 866 (10 µmol/L)	3.48 ± 0.91	7.80 ± 0.27
CLI 095 (1 µmol/L)	5.63 ± 0.65	7.61 ± 0.13
MCC 950 (100 nmol/L)	5.72 ± 0.44	8.04 ± 0.26
Anakinra (100 µg/mL)	6.04 ± 0.68	8.01 ± 0.22
Visfatin/eNampt (50 ng/mL) + FK 866 (10 µmol/L)	3.81 ± 0.96	$8.63 \pm 0.22^{+}$
Visfatin/eNampt (50 ng/mL) + CLI 095 (10 µmol/L)	4.11 ± 0.65	$7.65 \pm 0.15^{+}$
NMN (10 µmol/L) + CLI 095 (10 µmol/L)	5.33 ± 0.83	7.56 ± 0.20
Visfatin/eNampt (50 ng/mL) + MCC 950 (10 µmol/L)	5.56 ± 0.57	7.33 ± 0.22*
Visfatin/eNampt (50 ng/mL) + Anakinra (100 µg/mL)	6.04 ± 0.36	7.07 ± 0.20*
IL-1β (5 ng/mL)	5.01 ± 0.64	7.17 ± 0.41*
IL-1β (5 ng/mL) + Anakinra (100 µg/mL)	5.44 ± 0.63	$7.84 \pm 0.25^{\#}$
**Treatment**	**Tone prior to relaxation (mN)**	**pEC_{50} for SNP**
Control	4.38 ± 0.69	7.11 ± 0.22
IL-1β (5 ng/mL)	4.48 ± 0.43	7.68 ± 0.19
Visfatin/eNampt (50 ng/mL)	5.13 ± 0.72	6.56 ± 0.33

Mesenteric microvessels from untreated C57BL/6 mice were isolated and mounted in a myograph. After contracting the vessels with 3 µmol/L noradrenaline (NA), endothelium-dependent relaxations were studied by adding *ex vivo* cumulative concentrations of acetylcholine (ACh, 10 nmol/L to 10 µmol/L) into the organ bath in the absence of any other treatment or with the indicated drugs also administered *ex vivo*. Using a similar protocol, the endothelium-independent relaxations to sodium nitroprusside (SNP; 1 nmol/L to 10 nmol/L) were assessed. The contractile responses evoked by NA (in mNewtons, mN), as well as the respective pEC_{50} values for ACh or SNP are indicated (mean±SEM). *$p<0.05$ vs control; +$p<0.05$ vs respective visfatin/eNampt or NMN; #$p<0.05$ vs IL-1β.

Table S3. Vascular tone achieved with noradrenaline prior to relaxation curves and pEC_{50} values for ACh or SNP in isolated mesenteric microvessels from C57BL/6 mice infused for 7 days

In vivo administered drugs	Tone prior to relaxation (mN)	pEC_{50} for ACh
Saline solution (12 µL/day)	5.64 ± 0.23	7.79 ± 0.07
Visfatin/eNampt (100 ng/kg/day)	5.74 ± 0.20	6.95 ± 0.09*
FK 866 (2.4 mg/kg/day)	6.00 ± 0.61	7.70 ± 0.11
CLI 095 (3 mg/kg[a])	6.35 ± 0.38	7.49 ± 0.13
MCC 950 (10 mg/kg[a])	4.87 ± 0.36	8.18 ± 0.19
Anakinra (100 mg/kg[a])	6.49 ± 0.58	7.99 ± 0.14
Visfatin/eNampt (100 ng/kg/day) + FK 866 (2.4 mg/kg/day)	5.71 ± 0.39	7.51 ± 0.09[+]
Visfatin/eNampt (100 ng/kg/day) + CLI 095 (3 mg/kg[a])	6.25 ± 0.47	7.91 ± 0.21[+]
Visfatin/eNampt (100 ng/kg/day) + MCC 950 (10 mg/kg[a])	5.72 ± 0.60	7.87 ± 0.12[+]
Visfatin/eNampt (100 ng/kg/day) + Anakinra (100 mg/kg[a])	5.83 ± 0.65	7.94 ± 0.22[+]
IL-1β (12 µg/kg/day)	5.61 ± 0.66	7.14 ± 0.11*
IL-1β (12 µg/kg/day) + Anakinra (100 mg/kg[a])	4.48 ± 0.41	7.63 ± 0.11[#]
**In vivo administered drugs**	**Tone prior to relaxation (mN)**	**pEC_{50} for SNP**
Saline solution (12 µL/day)	4.82 ± 0.43	6.94 ± 0.17
Visfatin/eNampt (100 ng/kg/day)	5.73 ± 0.44	6.95 ± 0.15
FK 866 (2.4 mg/kg/day)	6.98 ± 1.34	6.73 ± 0.09
CLI 095 (3 mg/kg[a])	6.61 ± 1.02	6.60 ± 0.29
MCC 950 (10 mg/kg[a])	5.04 ± 0.56	8.00 ± 0.13
Anakinra (100 mg/kg[a])	5.00 ± 0.53	6.83 ± 0.20
Visfatin/eNampt (100 ng/kg/day) + FK 866 (2.4 mg/kg/day)	4.69 ± 0.58	6.69 ± 0.20
Visfatin/eNampt (100 ng/kg/day) + CLI 095 (3 mg/kg[a])	6.26 ± 0.56	6.71 ± 0.27
Visfatin/eNampt (100 ng/kg/day) + MCC 950 (10 mg/kg[a])	5.85 ± 0.85	7.65 ± 0.17
Visfatin/eNampt (100 ng/kg/day) + Anakinra (100 mg/kg[a])	4.58 ± 0.84	6.62 ± 0.39
IL-1β (12 µg/kg/day)	4.15 ± 0.41	6.95 ± 0.15
IL-1β (12 µg/kg/day) + Anakinra (100 mg/kg[a])	4.32 ± 0.60	6.73 ± 0.31

Visfatin/eNampt, FK 866, visfatin/eNampt plus FK 866, and IL-1β were infused for 7 days in C57BL/6 mice through osmotic minipumps. [a]These animals received i.p. CLI 095 (days 1 to 6), MCC 950 (days 2, 4, and 6), or anakinra (days 4, 5, and 6) before sacrifice. Afterwards, mesenteric microvessels were isolated and mounted in a myograph. The vessels were then pre-contracted with 3 µmol/L noradrenalilne (NA), and the endothelium-dependent relaxations were studied by adding cumulative concentrations of acetylcholine (ACh, 10 nmol/L to 10 µmol/L) into the organ bath. Using a similar protocol, the endothelium-independent relaxations to sodium nitroprusside (SNP; 1 nmol/L to 10 nmol/L) were studied. The contractile responses by NA (in mNewtons, mN), as well as the respective pEC_{50} values for ACh or SNP are indicated (mean±SEM). *$p<0.05$ vs saline solution; +$p<0.05$ vs visfatin/eNampt; #$p<0.05$ vs IL-1β.

Figure S1

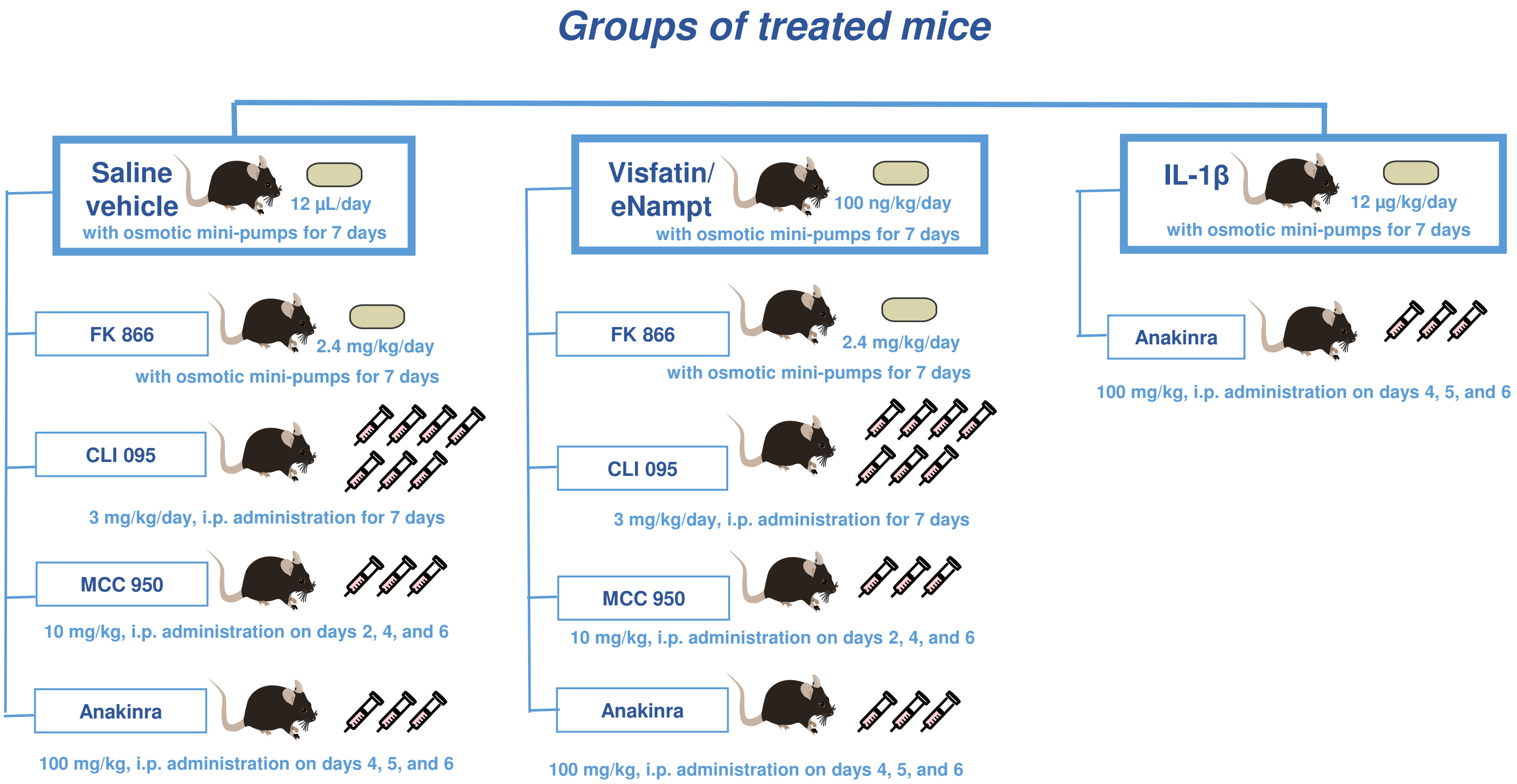

Groups of treated mice
Saline vehicle
12 µL/day
with osmotic mini-pumps for 7 days
Visfatin/ eNampt
100 ng/kg/day
with osmotic mini-pumps for 7 days
IL-1β
12 µg/kg/day
with osmotic mini-pumps for 7 days
FK 866
2.4 mg/kg/day
with osmotic mini-pumps for 7 days
CLI 095
3 mg/kg/day, i.p. administration for 7 days
MCC 950
10 mg/kg, i.p. administration on days 2, 4, and 6
Anakinra
100 mg/kg, i.p. administration on days 4, 5, and 6
FK 866
2.4 mg/kg/day
with osmotic mini-pumps for 7 days
CLI 095
3 mg/kg/day, i.p. administration for 7 days
MCC 950
10 mg/kg, i.p. administration on days 2, 4, and 6
Anakinra
100 mg/kg, i.p. administration on days 4, 5, and 6
Anakinra
100 mg/kg, i.p. administration on days 4, 5, and 6

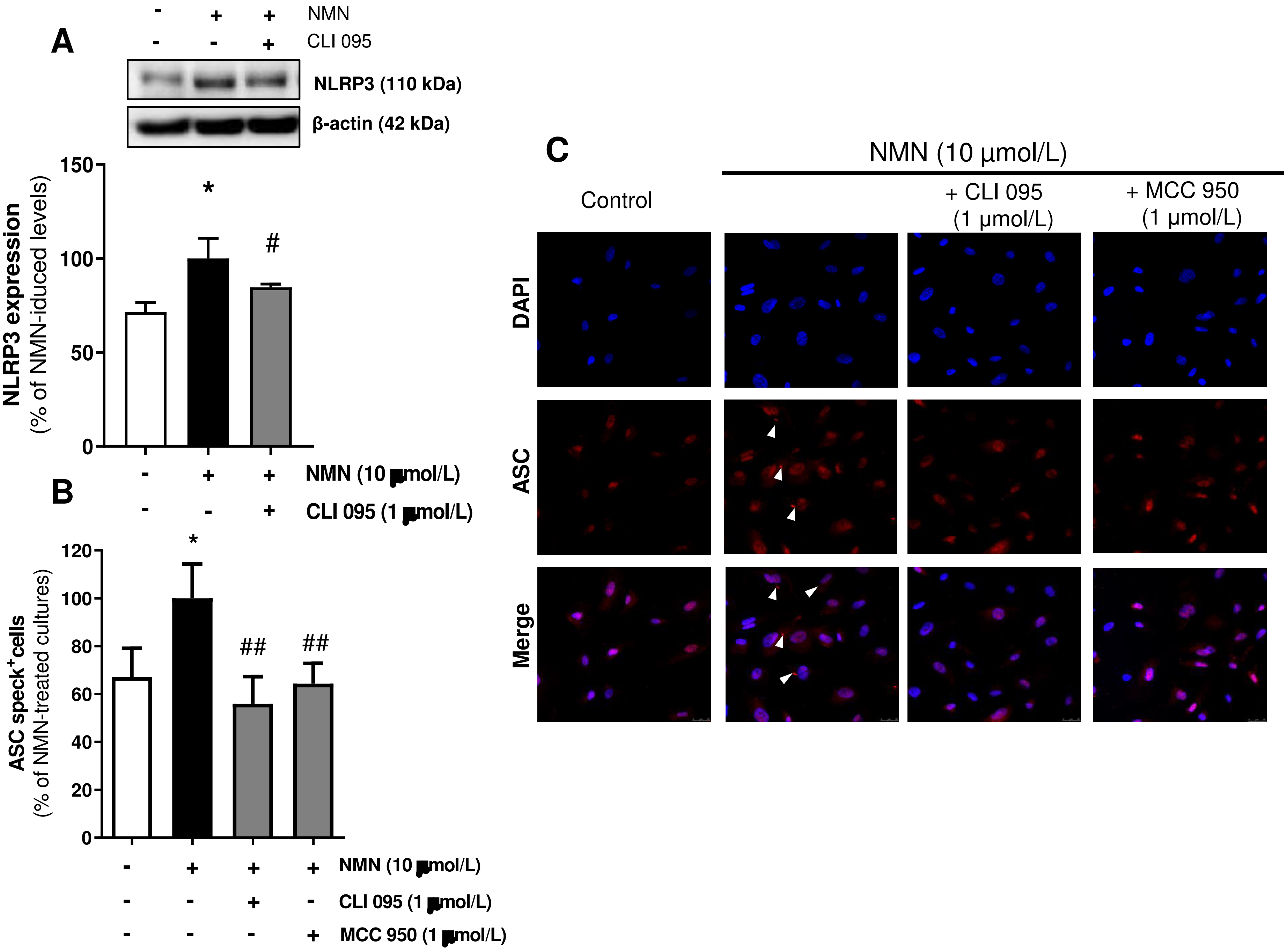
A
- + + NMN
- - + CLI 095
NLRP3 (110 kDa)
β-actin (42 kDa)
NLRP3 expression
(% of NMN-iduced levels)
150
100
50
0
*
#
NMN (10 μmol/L)
CLI 095 (1 μmol/L)
B
ASC speck+cells
(% of NMN-treated cultures)
120
100
80
60
40
20
0
*
##
##
NMN (10 μmol/L)
CLI 095 (1 μmol/L)
MCC 950 (1 μmol/L)
C
NMN (10 μmol/L)
Control
+ CLI 095 (1 μmol/L)
+ MCC 950 (1 μmol/L)
DAPI
ASC
Merge

Figure S3

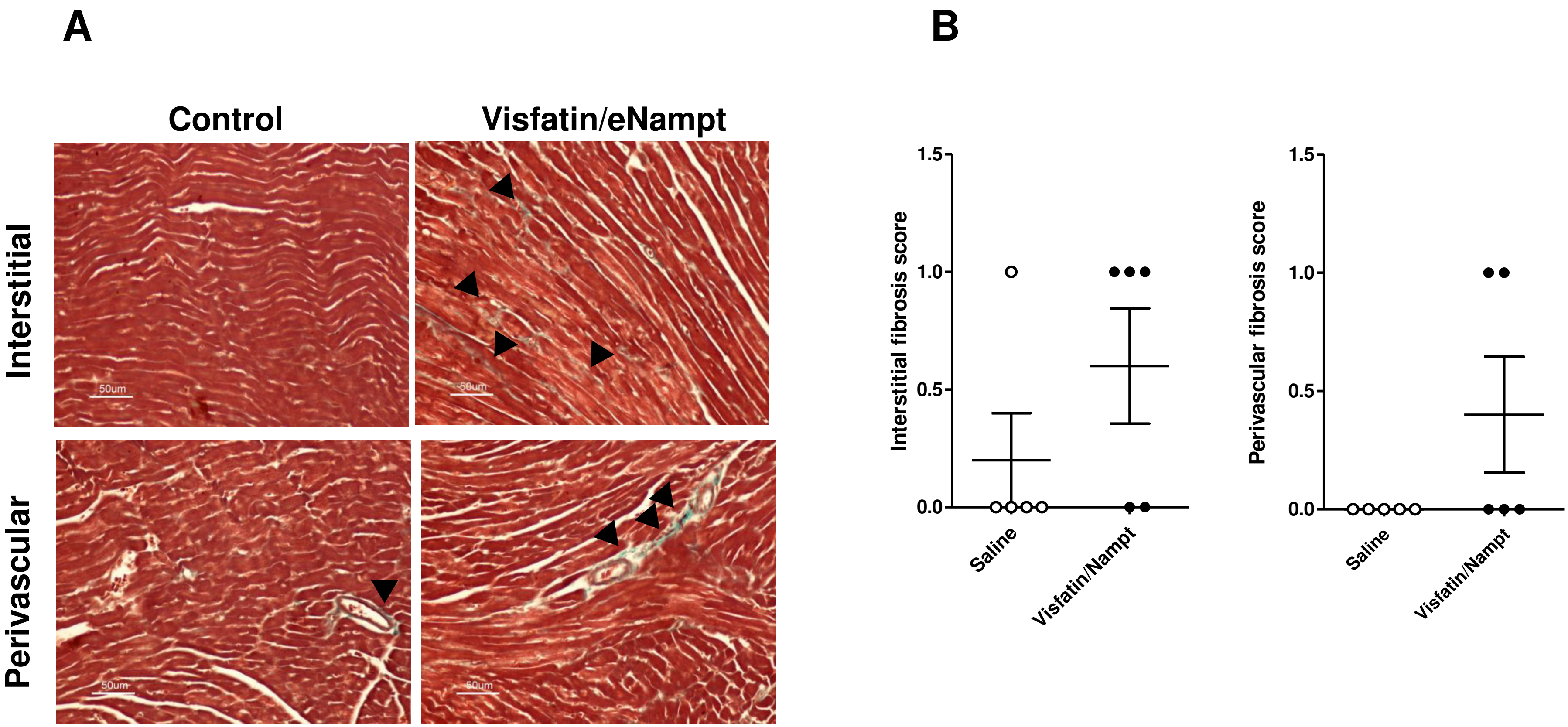
A
Control
Visfatin/eNampt
Interstitial
Perivascular
50um
50um
50um
50um
B
1.5
1.0
0.5
0.0
Interstitial fibrosis score
Saline
Visfatin/Nampt
1.5
1.0
0.5
0.0
Perivascular fibrosis score
Saline
Visfatin/Nampt

Figure S4

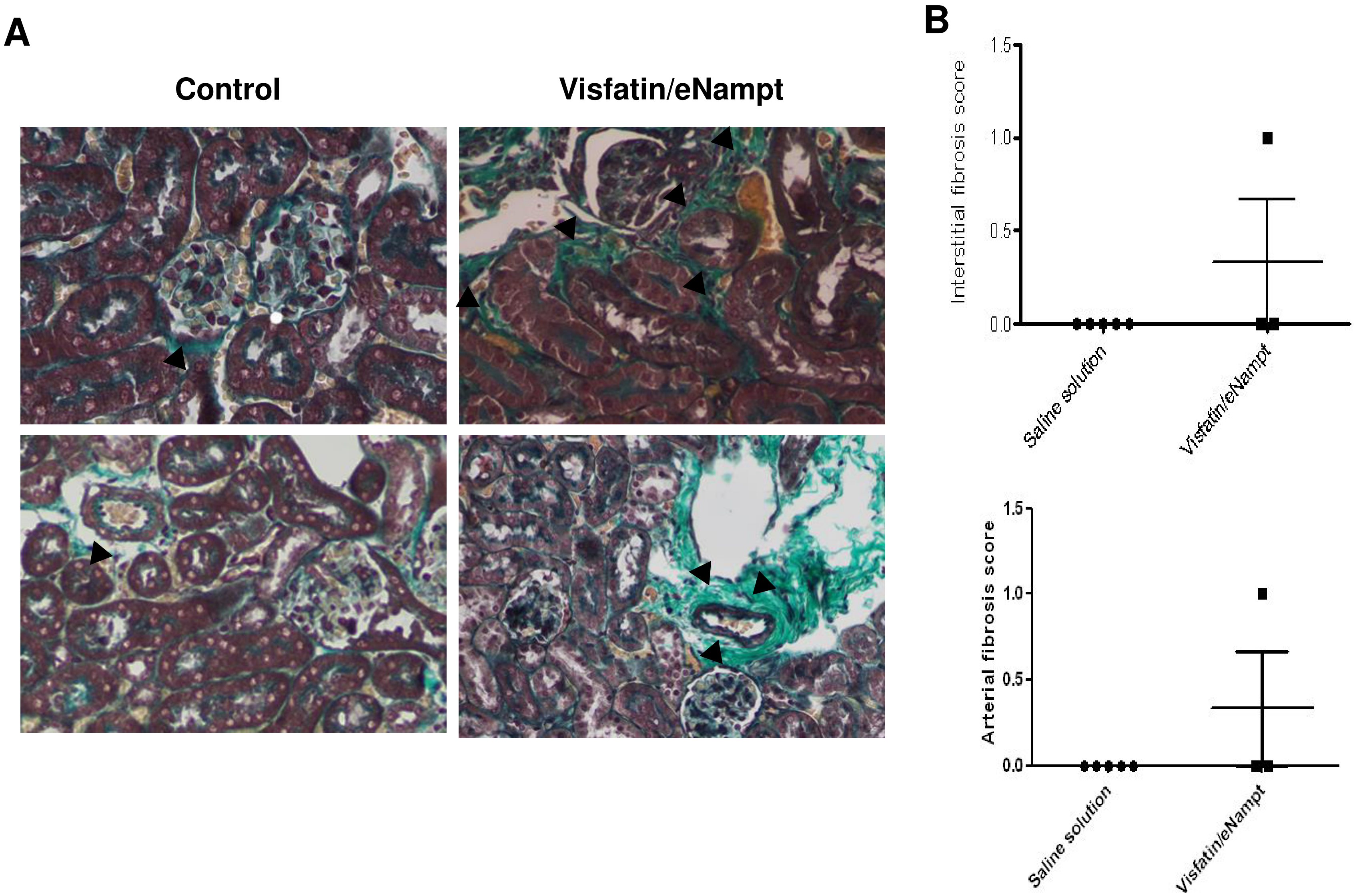
A
Control
Visfatin/eNampt
Interstitial
Perivascular
B
1.5
1.0
0.5
0.0
Interstitial fibrosis score
Saline solution
Visfatin/eNampt
1.5
1.0
0.5
0.0
Arterial fibrosis score
Saline solution
Visfatin/eNampt

Figure S5

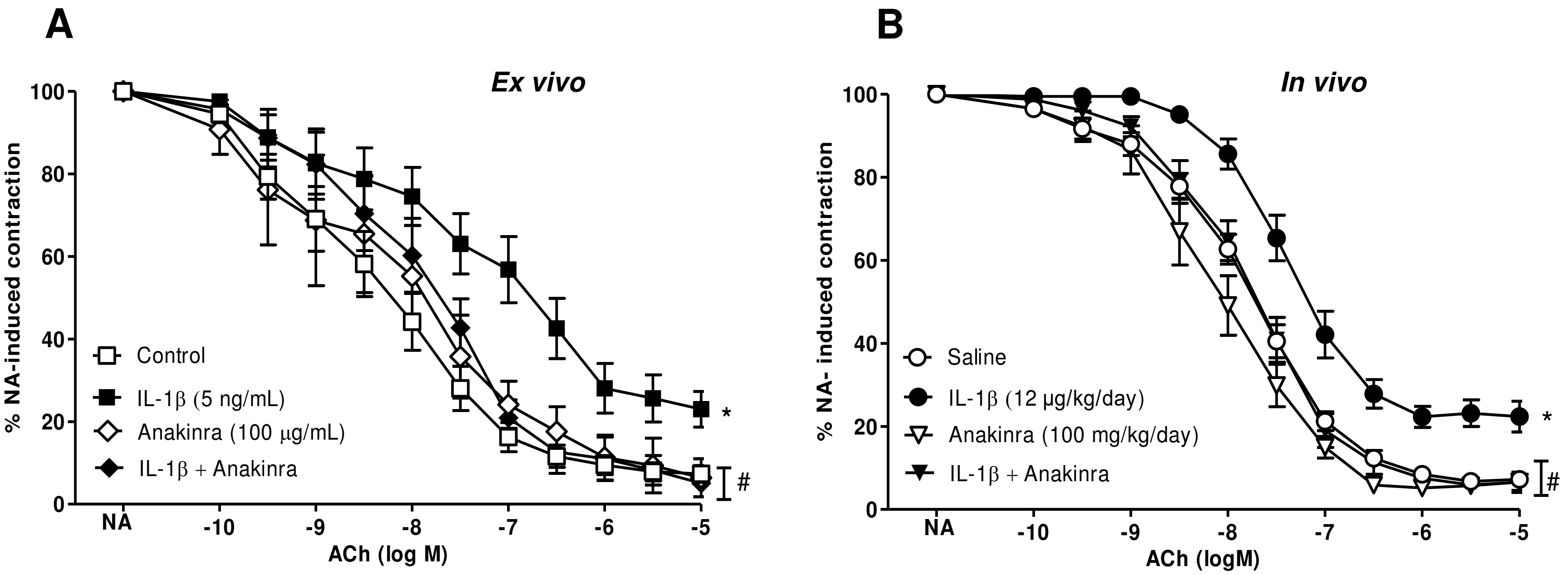
A
Ex vivo
% NA-induced contraction
100
80
60
40
20
0
NA
-10
-9
-8
-7
-6
-5
ACh (log M)
Control
IL-1β (5 ng/mL)
Anakinra (100 μg/mL)
IL-1β + Anakinra
*
#
B
In vivo
% NA- induced contraction
ACh (logM)
Saline
IL-1β (12 μg/kg/day)
Anakinra (100 mg/kg/day)
IL-1β + Anakinra

Uncropped Gels shown as representative blots in Main & Supplementary Figures

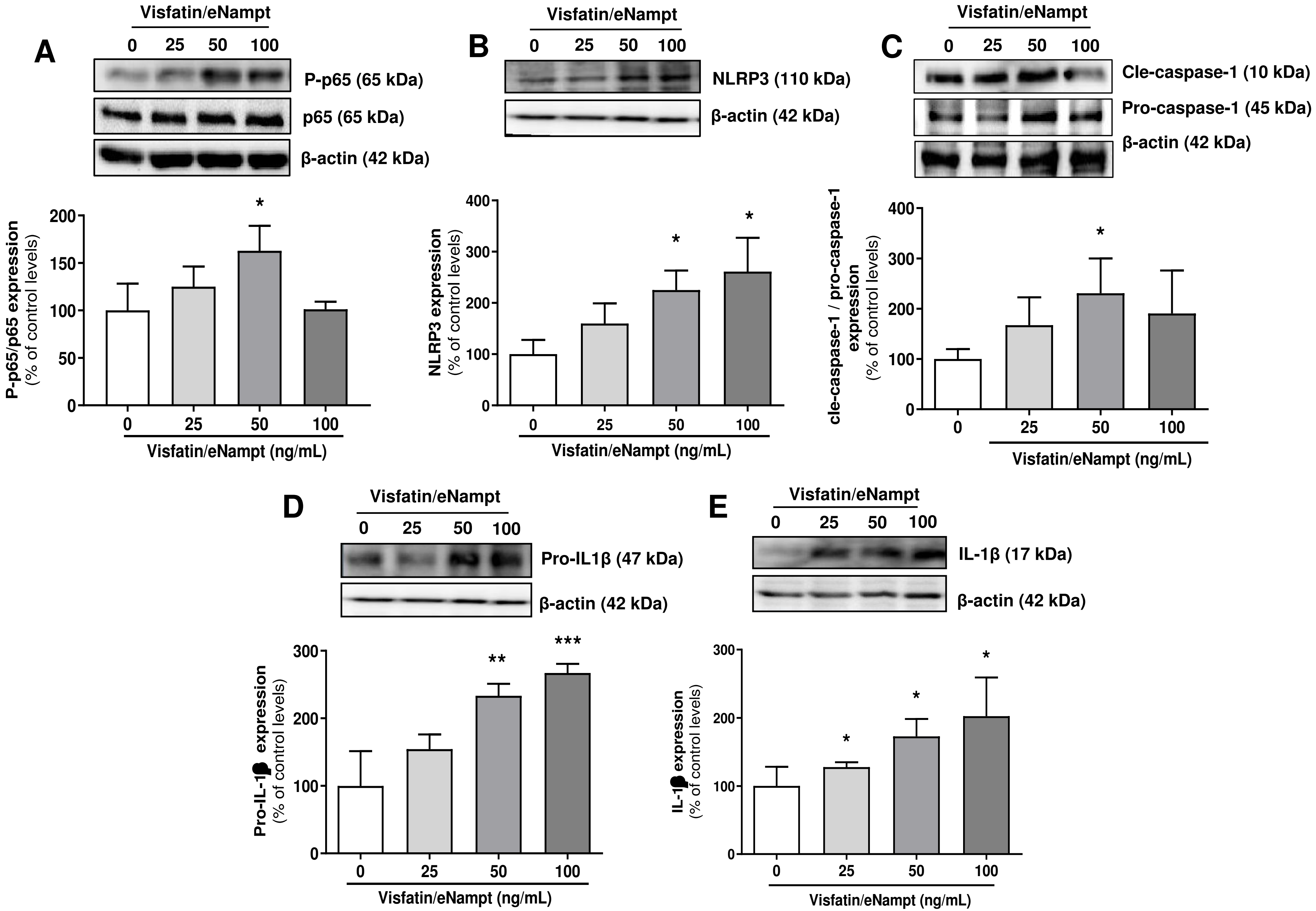
A
Visfatin/eNampt
0 25 50 100
P-p65 (65 kDa)
p65 (65 kDa)
β-actin (42 kDa)
P-p65/p65 expression
(% of control levels)
Visfatin/eNampt (ng/mL)
B
NLRP3 (110 kDa)
NLRP3 expression
C
Cle-caspase-1 (10 kDa)
Pro-caspase-1 (45 kDa)
cle-caspase-1 / pro-caspase-1 expression
D
Pro-IL1β (47 kDa)
Pro-IL-1β expression
E
IL-1β (17 kDa)
IL-1β expression

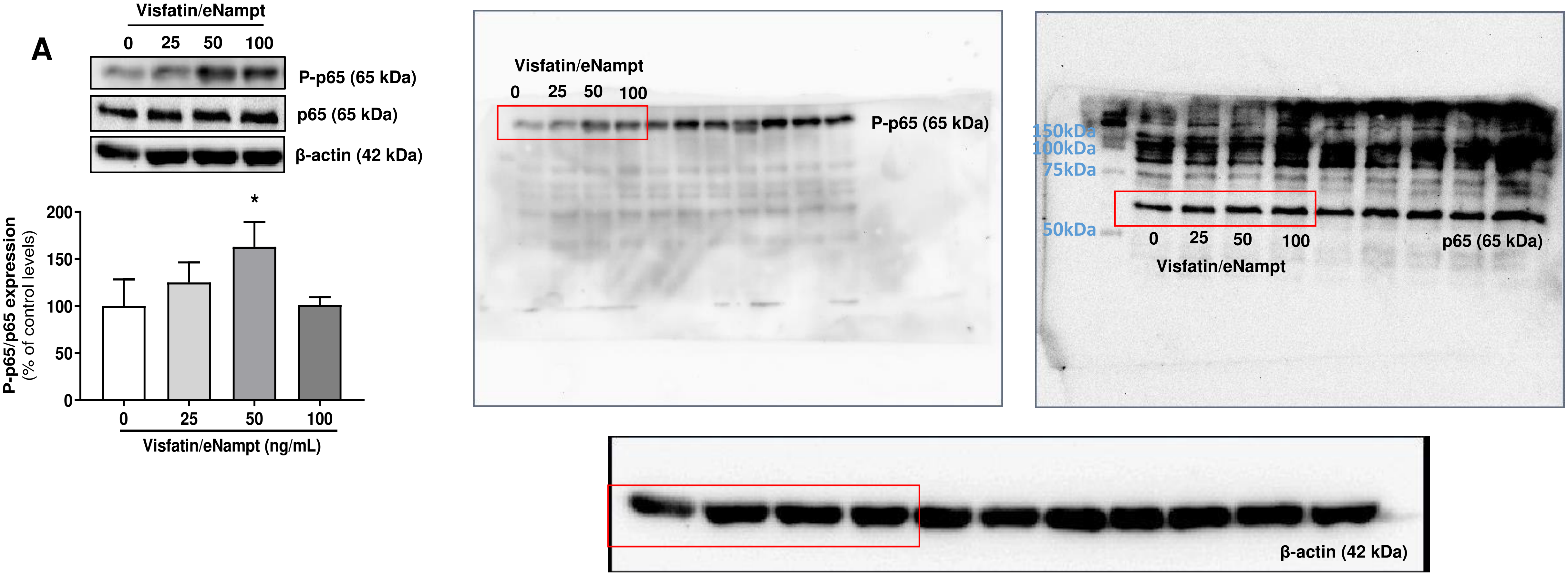
A
Visfatin/eNampt
0
25
50
100
P-p65 (65 kDa)
p65 (65 kDa)
β-actin (42 kDa)
P-p65/p65 expression
(% of control levels)
200
150
100
50
0
*
0
25
50
100
Visfatin/eNampt (ng/mL)
Visfatin/eNampt
0
25
50
100
P-p65 (65 kDa)
150kDa
100kDa
75kDa
50kDa
0
25
50
100
Visfatin/eNampt
p65 (65 kDa)
β-actin (42 kDa)

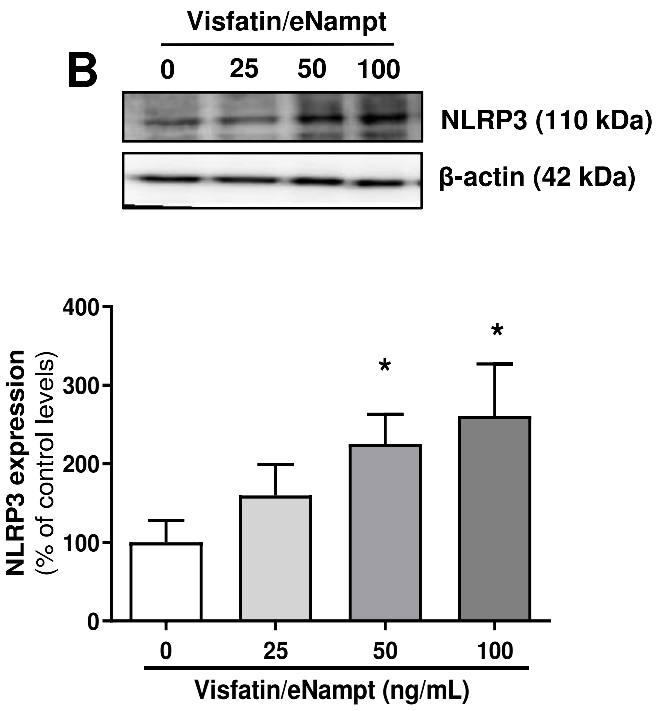
B
Visfatin/eNampt
0
25
50
100
NLRP3 (110 kDa)
β-actin (42 kDa)
NLRP3 expression
(% of control levels)
400
300
200
100
0
*
*
Visfatin/eNampt (ng/mL)

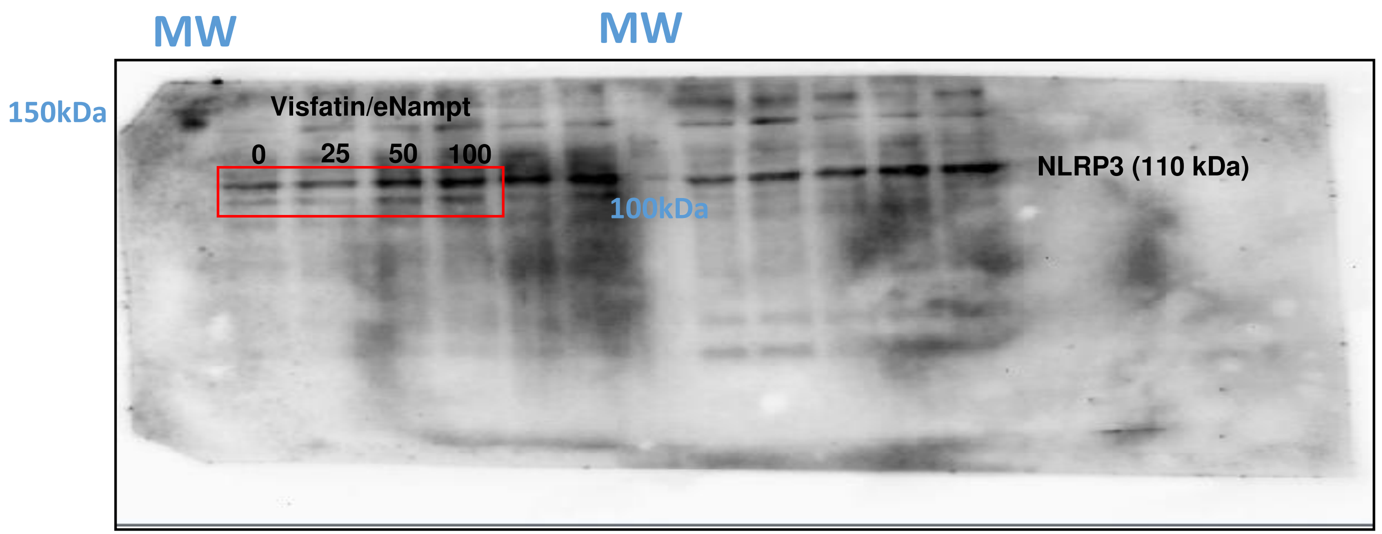
MW
MW
150kDa
Visfatin/eNampt
0
25
50
100
NLRP3 (110 kDa)
100kDa

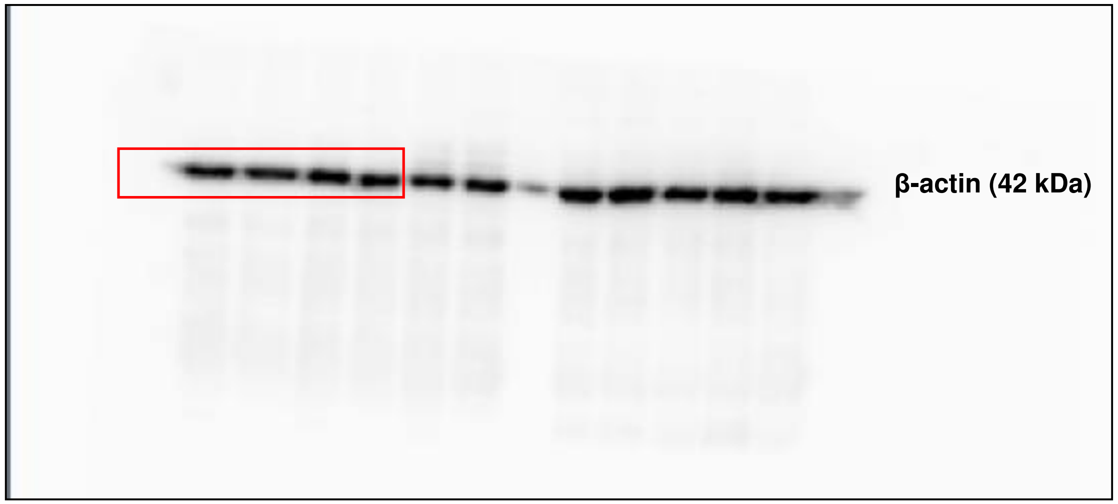
β-actin (42 kDa)

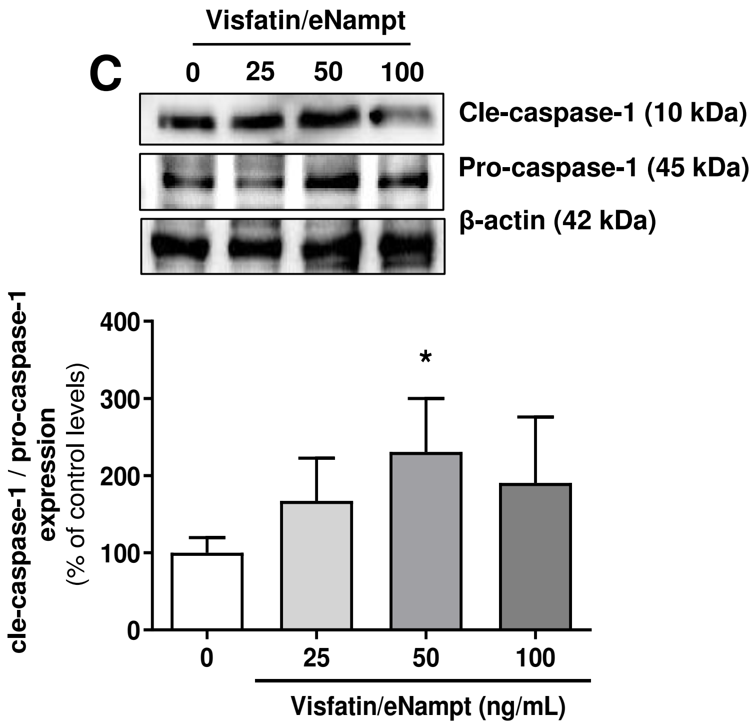
C
Visfatin/eNampt
0
25
50
100
Cle-caspase-1 (10 kDa)
Pro-caspase-1 (45 kDa)
β-actin (42 kDa)
cle-caspase-1 / pro-caspase-1 expression
(% of control levels)
400
300
200
100
0
*
0
25
50
100
Visfatin/eNampt (ng/mL)

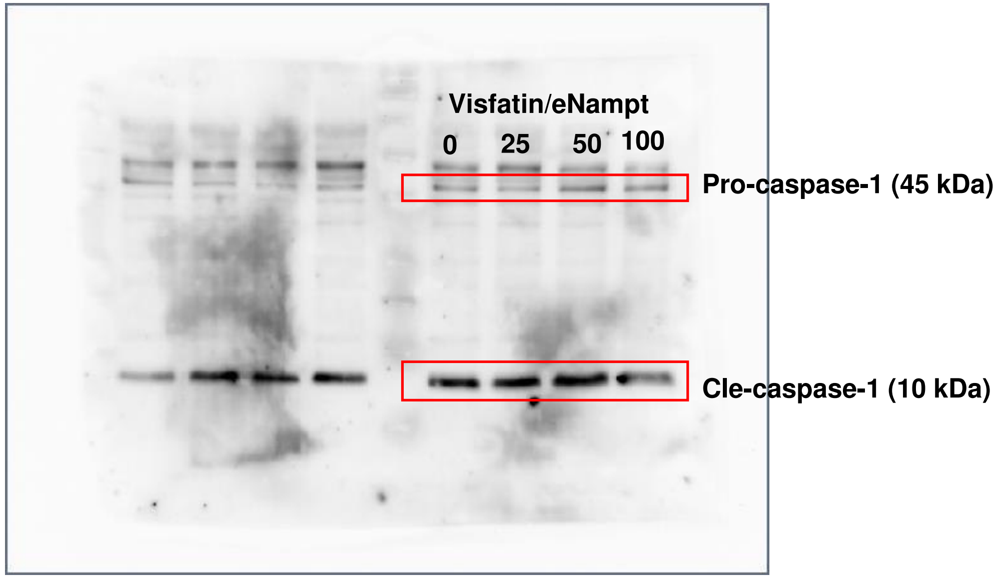
Visfatin/eNampt
0
25
50
100
Pro-caspase-1 (45 kDa)
Cle-caspase-1 (10 kDa)

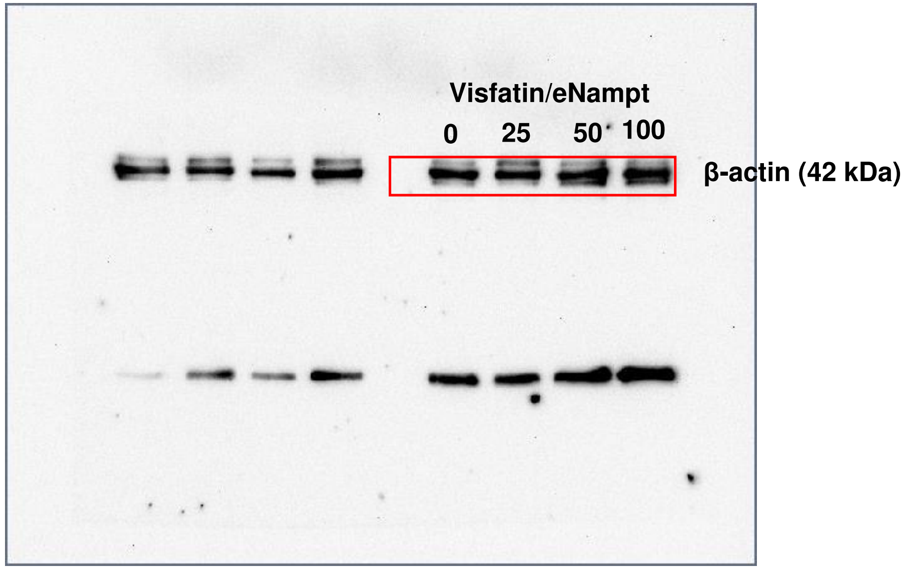
Visfatin/eNampt
0
25
50
100
β-actin (42 kDa)

D

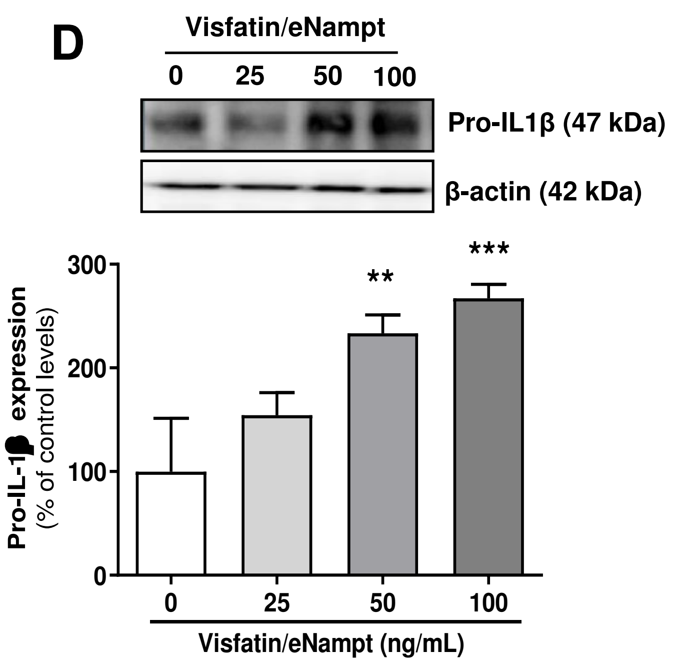
Visfatin/eNampt
0
25
50
100
Pro-IL1β (47 kDa)
β-actin (42 kDa)
Pro-IL-1β expression
(% of control levels)
300
200
100
0
**
***
0
25
50
100
Visfatin/eNampt (ng/mL)

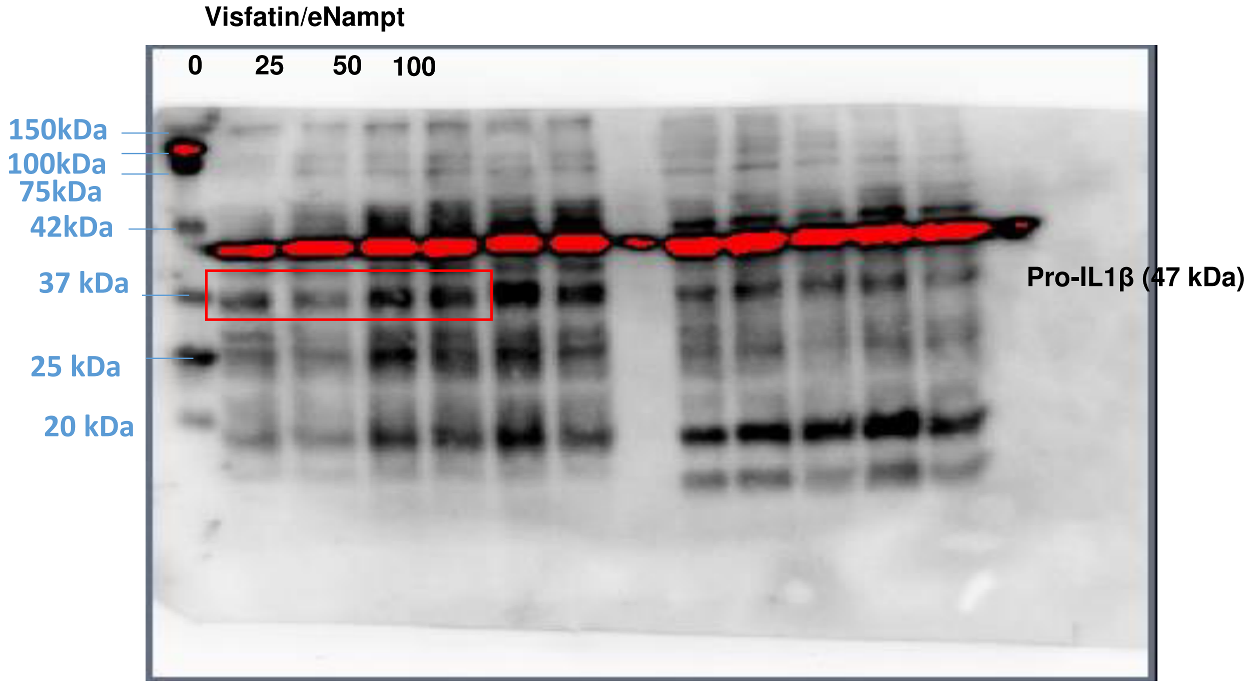
Visfatin/eNampt
0
25
50
100
150kDa
100kDa
75kDa
42kDa
37 kDa
25 kDa
20 kDa
Pro-IL1β (47 kDa)

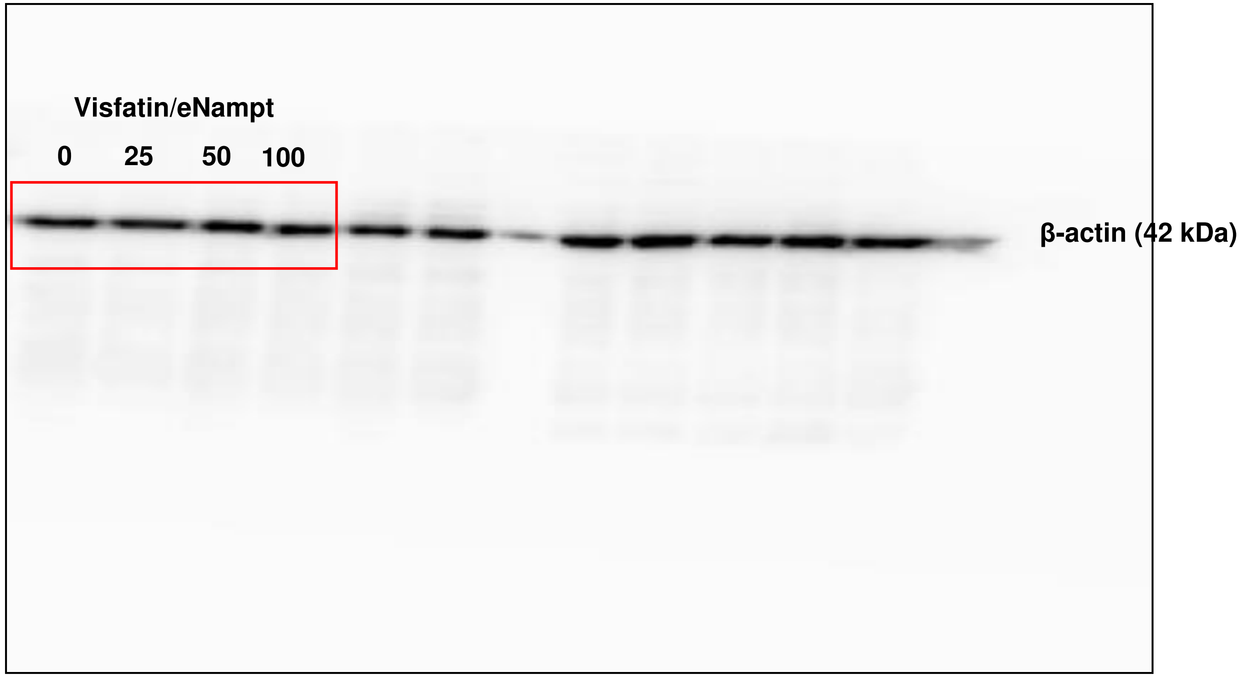
Visfatin/eNampt
0
25
50
100
β-actin (42 kDa)

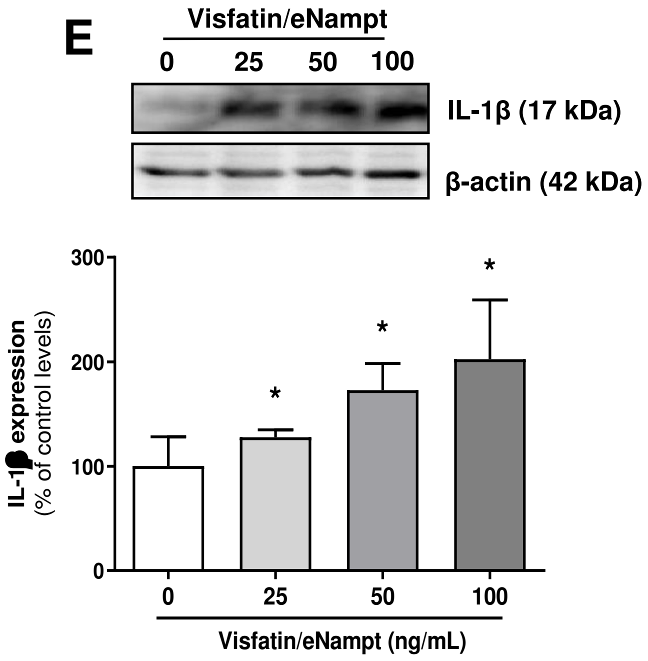
E
Visfatin/eNampt
0
25
50
100
IL-1β (17 kDa)
β-actin (42 kDa)
300
200
100
0
IL-1β expression
(% of control levels)
*
*
*
0
25
50
100
Visfatin/eNampt (ng/mL)

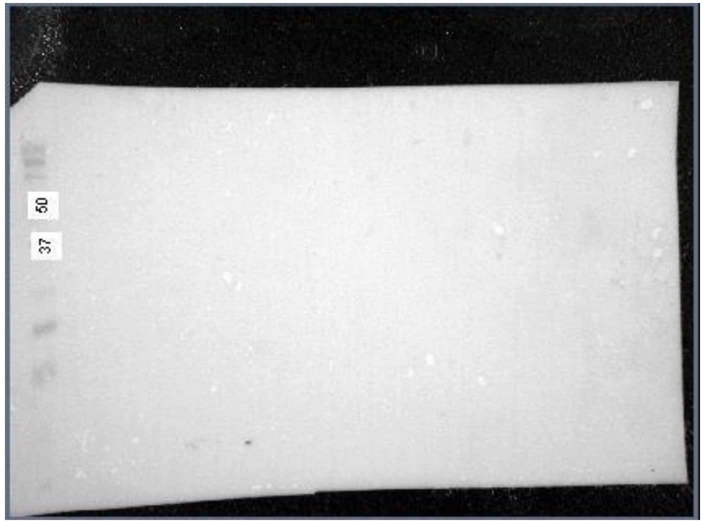
50
37

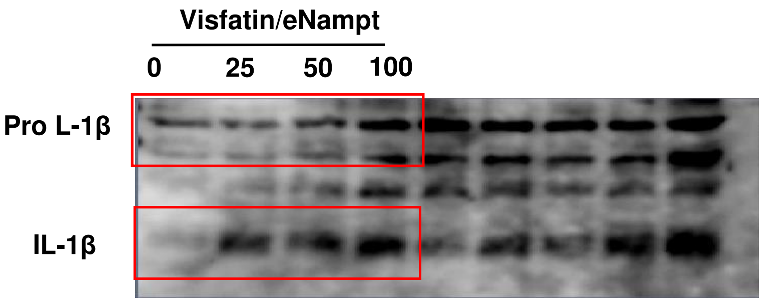
Visfatin/eNampt
0
25
50
100
Pro L-1β
IL-1β

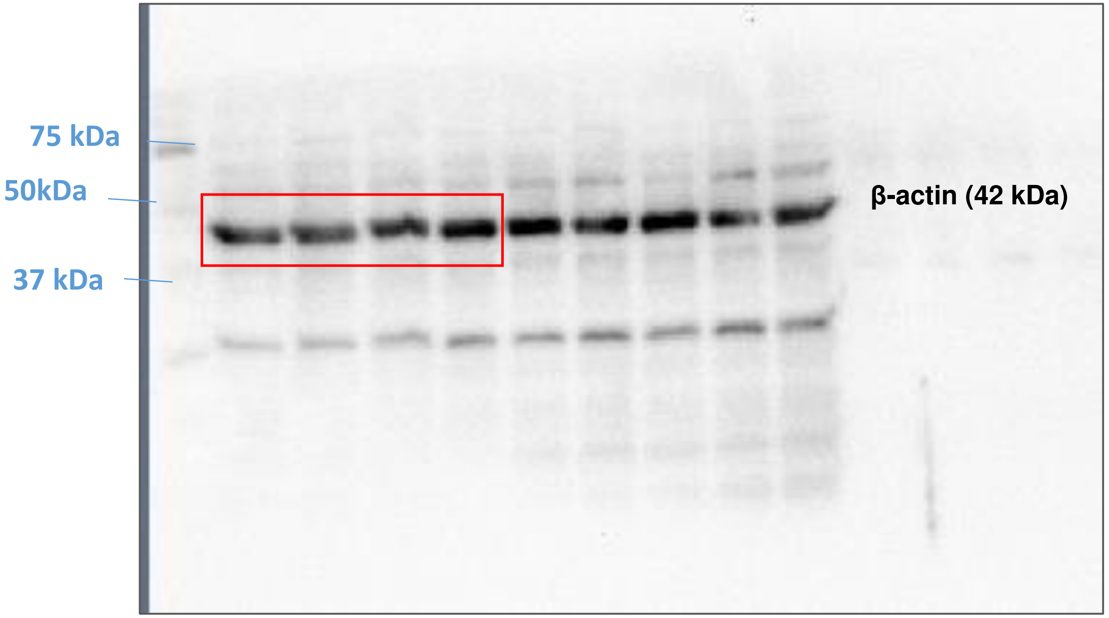
75 kDa
50kDa
37 kDa
β-actin (42 kDa)

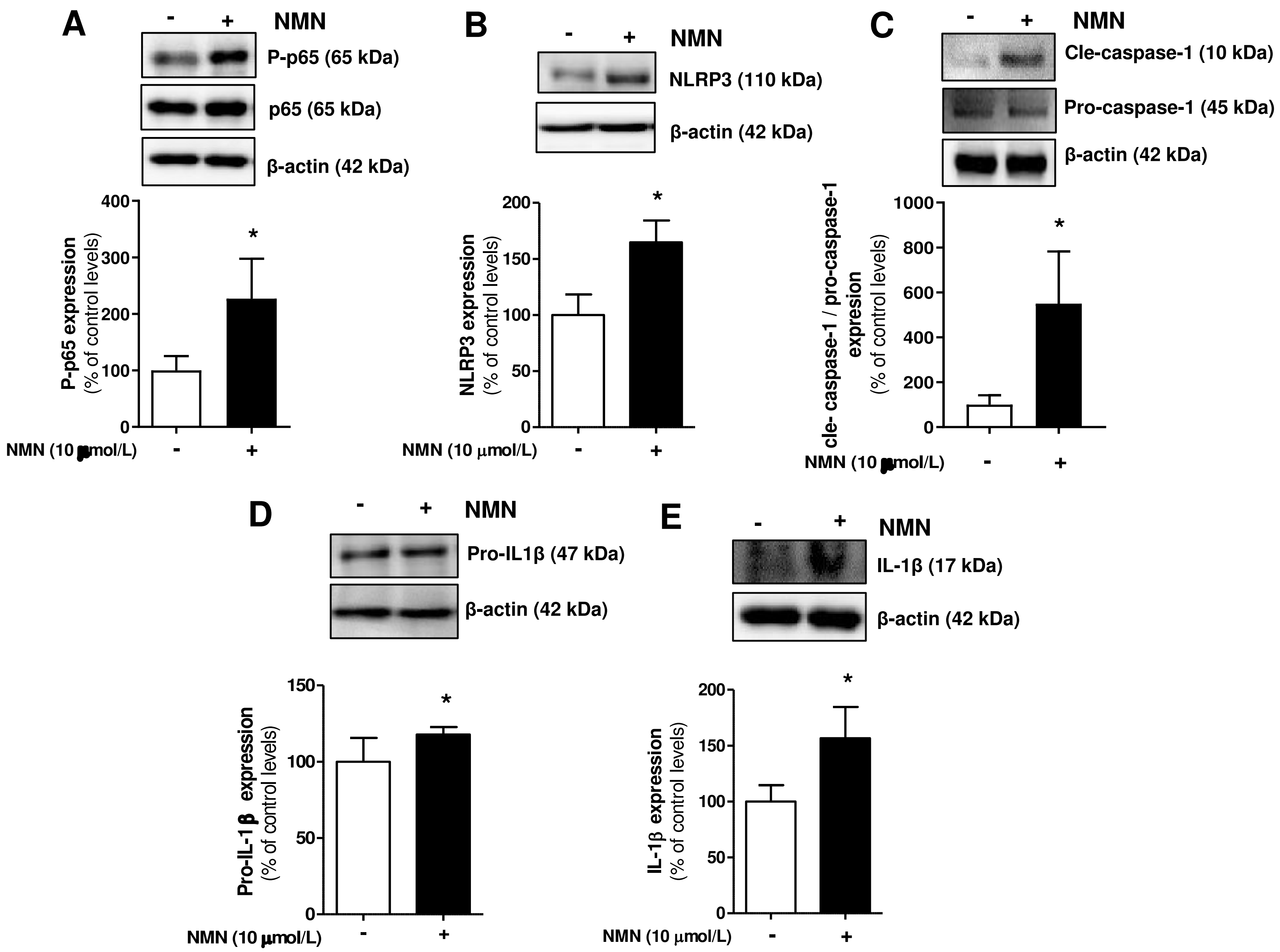
A
NMN
P-p65 (65 kDa)
p65 (65 kDa)
β-actin (42 kDa)
P-p65 expression (% of control levels)
NMN (10 μmol/L)
B
NLRP3 (110 kDa)
β-actin (42 kDa)
NLRP3 expression (% of control levels)
C
Cle-caspase-1 (10 kDa)
Pro-caspase-1 (45 kDa)
β-actin (42 kDa)
cle- caspase-1 / pro-caspase-1 expresion (% of control levels)
D
Pro-IL1β (47 kDa)
β-actin (42 kDa)
Pro-IL-1β expression (% of control levels)
E
IL-1β (17 kDa)
β-actin (42 kDa)
IL-1β expression (% of control levels)

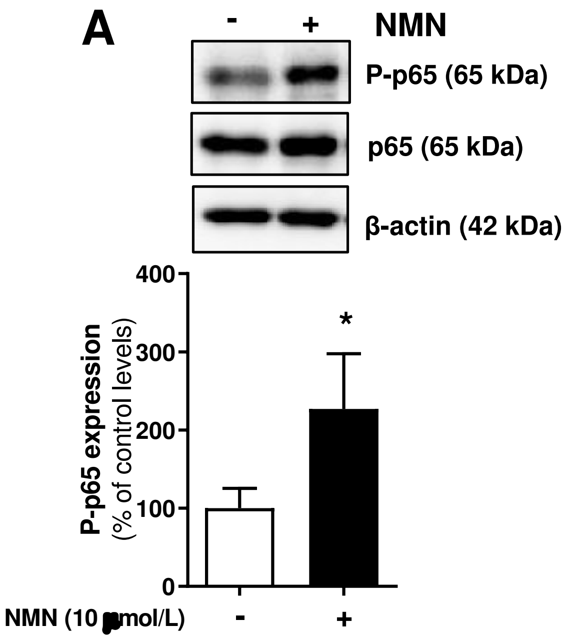
A
- + NMN
P-p65 (65 kDa)
p65 (65 kDa)
β-actin (42 kDa)
P-p65 expression
(% of control levels)
400
300
200
100
0
*
NMN (10 µmol/L) - +

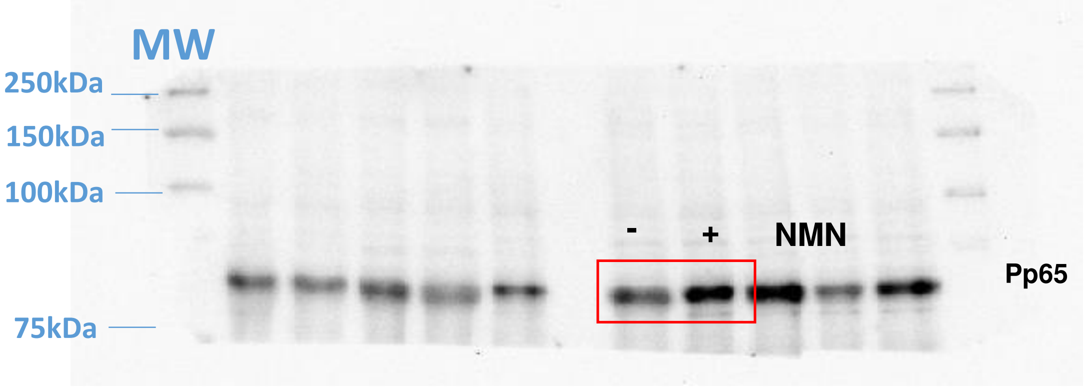
MW
250kDa
150kDa
100kDa
75kDa
- + NMN
Pp65

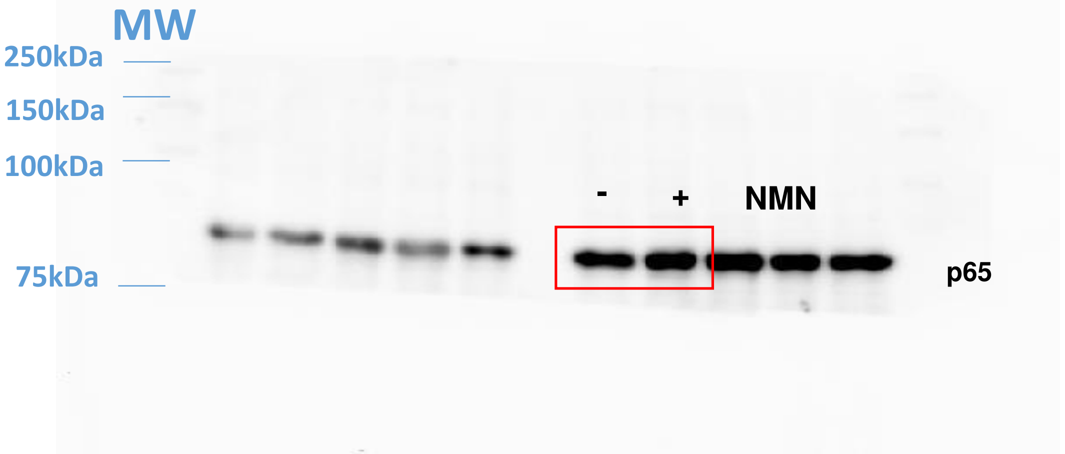
MW
250kDa
150kDa
100kDa
75kDa
- + NMN
p65

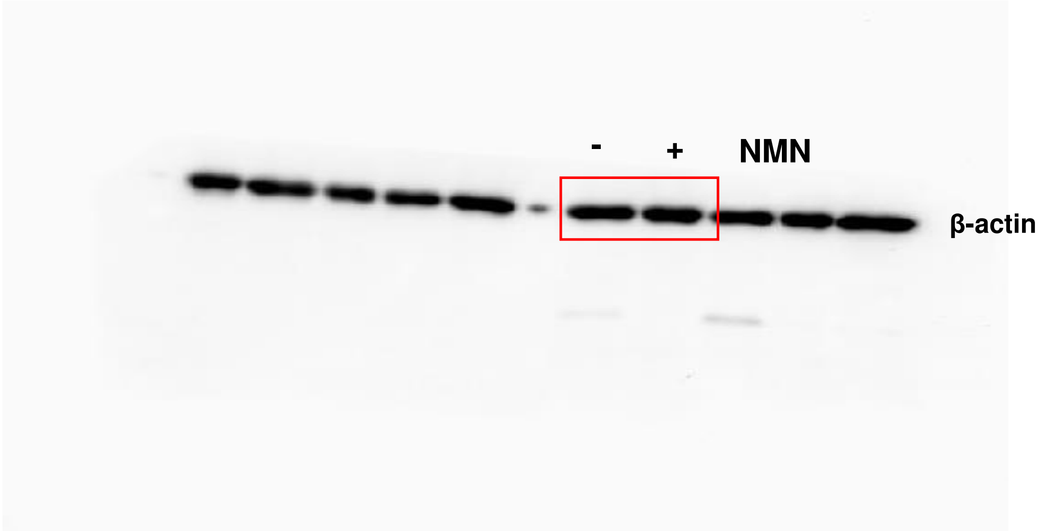
- + NMN
β-actin

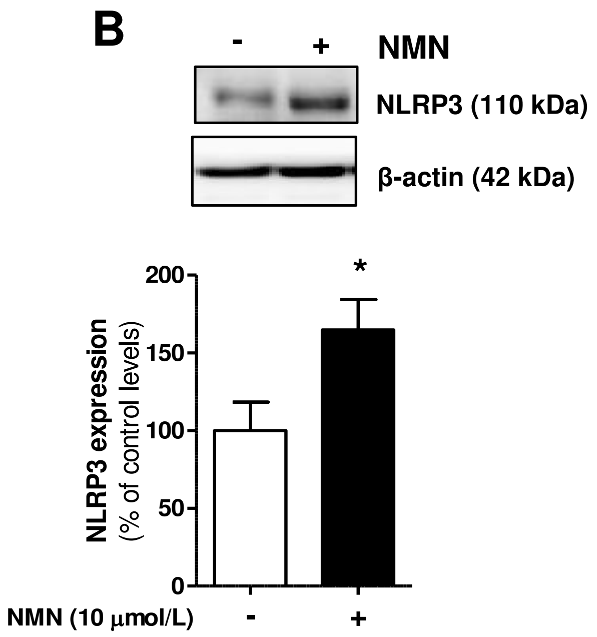
B
- + NMN
NLRP3 (110 kDa)
β-actin (42 kDa)
NLRP3 expression
(% of control levels)
200
150
100
50
0
*
NMN (10 µmol/L) - +

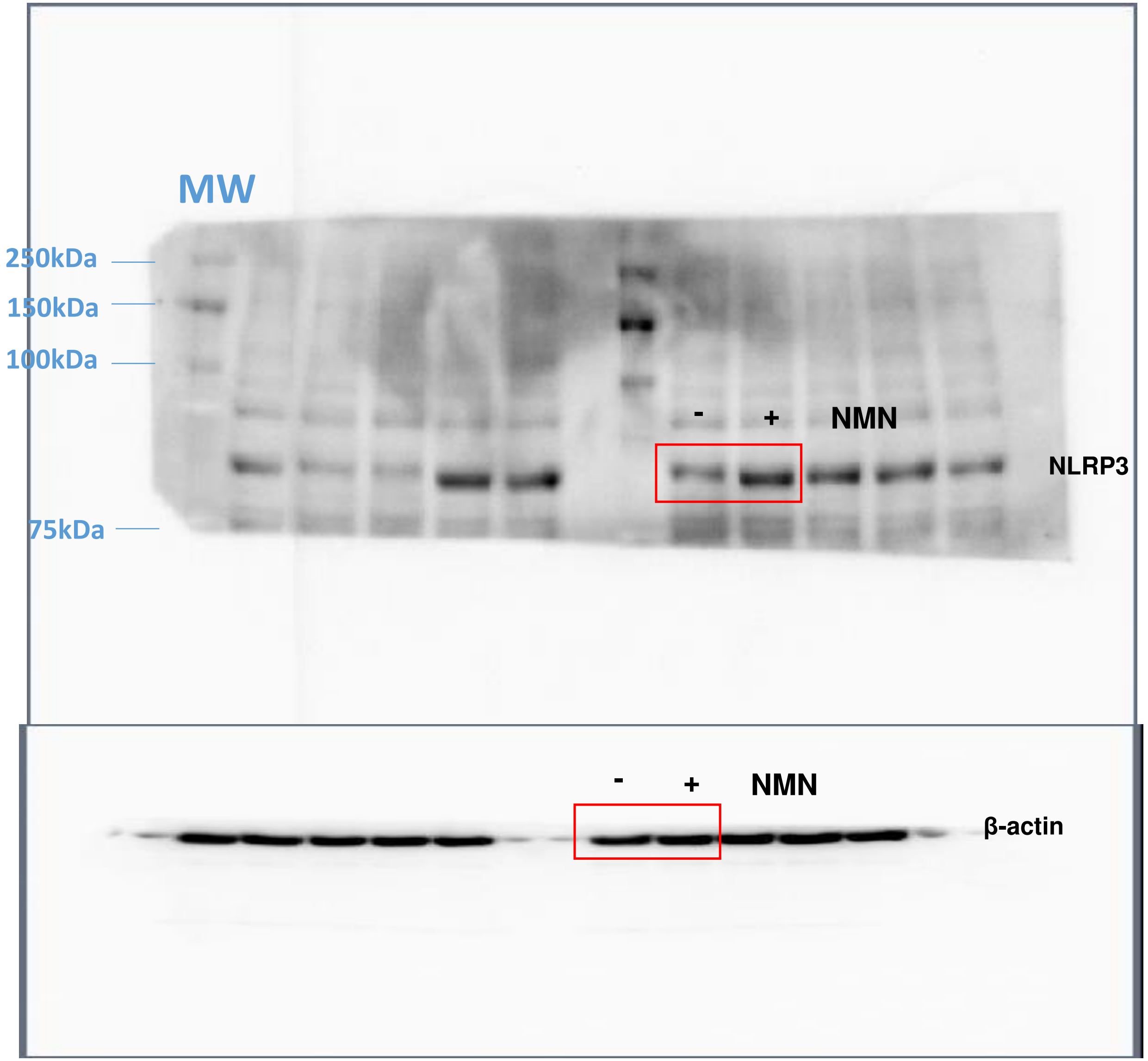
MW
250kDa
150kDa
100kDa
75kDa
- + NMN
NLRP3
- + NMN
β-actin

C

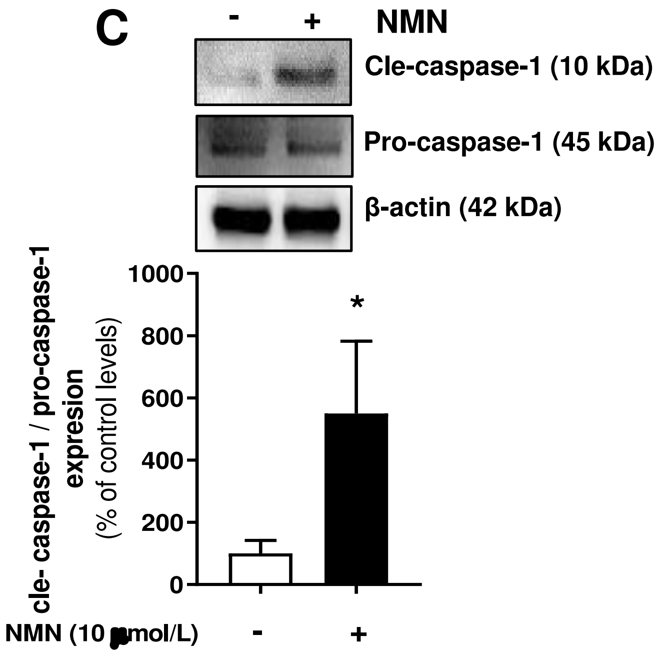
- + NMN
Cle-caspase-1 (10 kDa)
Pro-caspase-1 (45 kDa)
β-actin (42 kDa)
cle- caspase-1 / pro-caspase-1 expresion
(% of control levels)
1000
800
600
400
200
0
*
NMN (10 µmol/L) - +

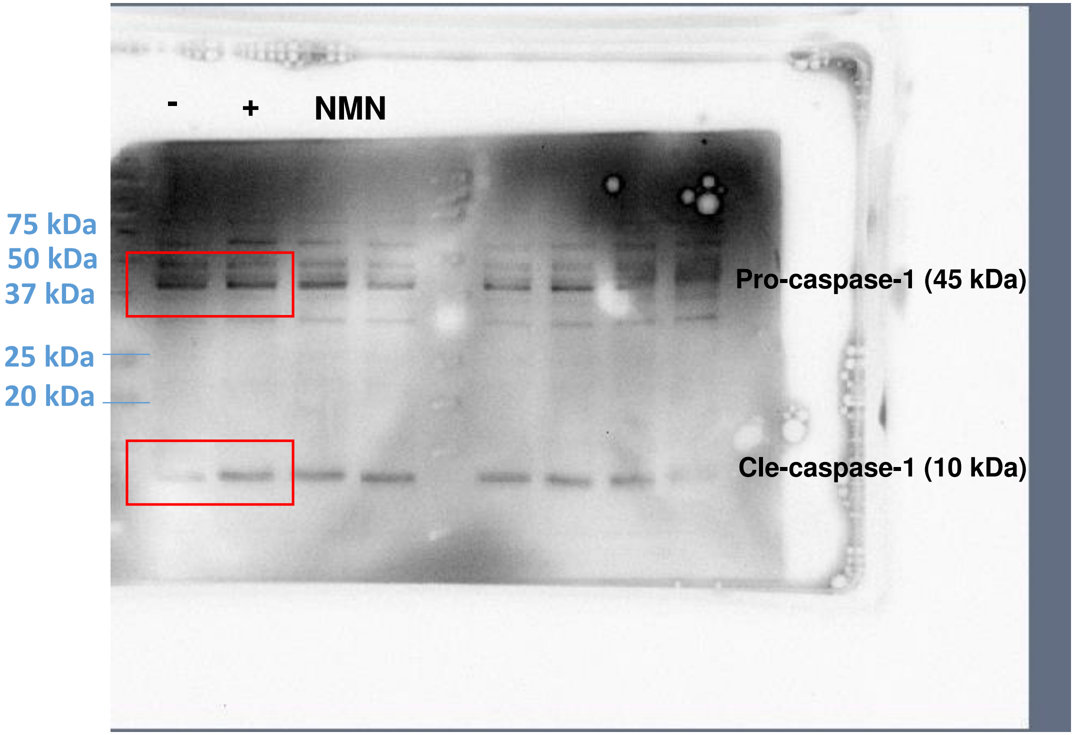
- + NMN
75 kDa
50 kDa
37 kDa
25 kDa
20 kDa
Pro-caspase-1 (45 kDa)
Cle-caspase-1 (10 kDa)

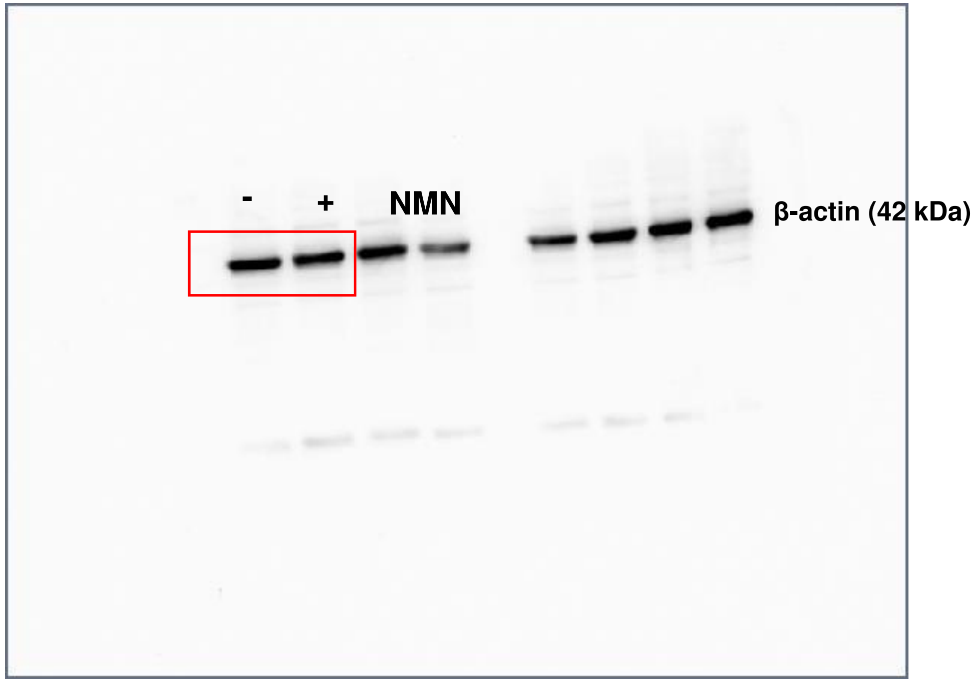
- + NMN
β-actin (42 kDa)

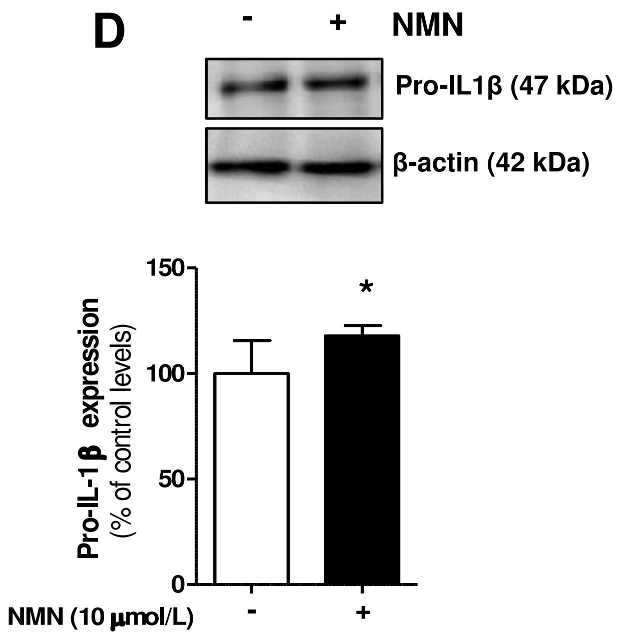
D
- + NMN
Pro-IL1β (47 kDa)
β-actin (42 kDa)
150
100
50
0
*
Pro-IL-1β expression
(% of control levels)
NMN (10 µmol/L) - +

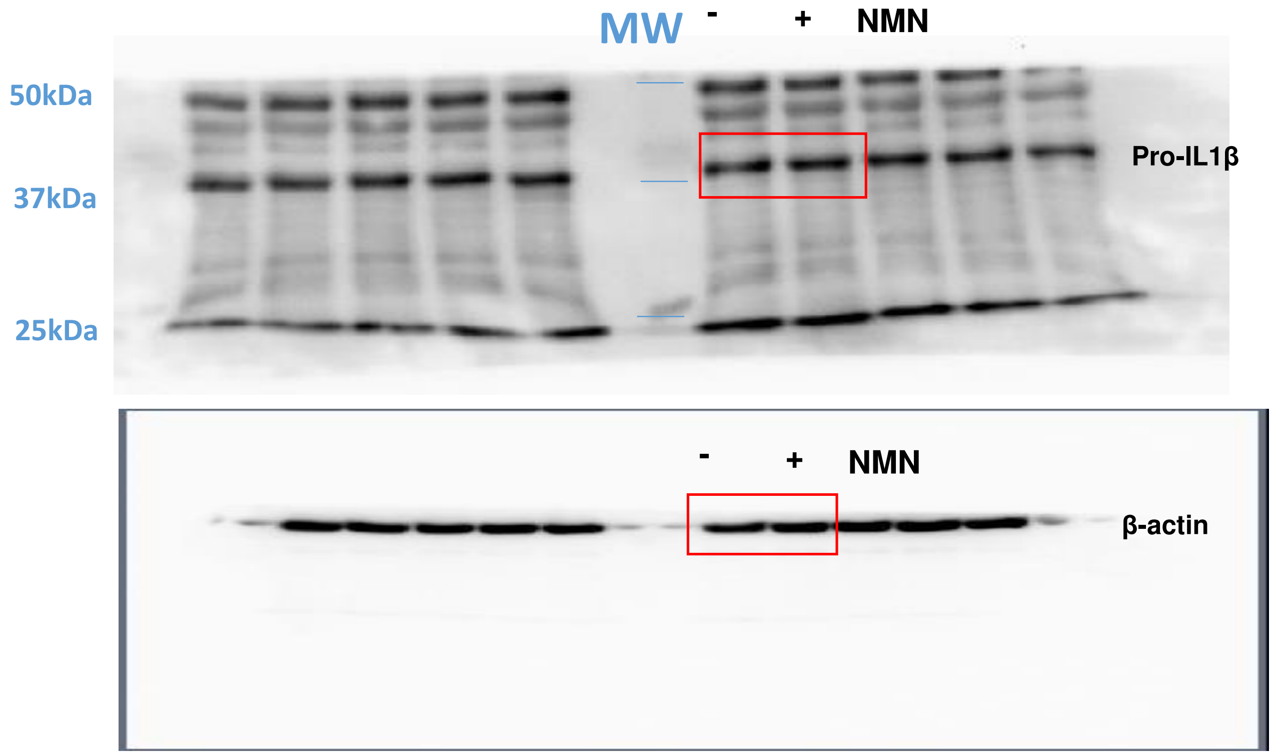
MW - + NMN
50kDa
37kDa
25kDa
Pro-IL1β
- + NMN
β-actin

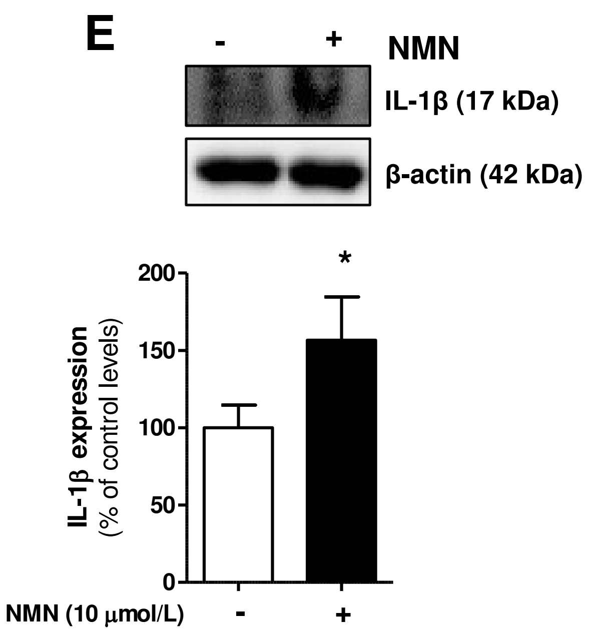
E
- + NMN
IL-1β (17 kDa)
β-actin (42 kDa)
*
200
150
100
50
0
IL-1β expression
(% of control levels)
NMN (10 μmol/L) - +

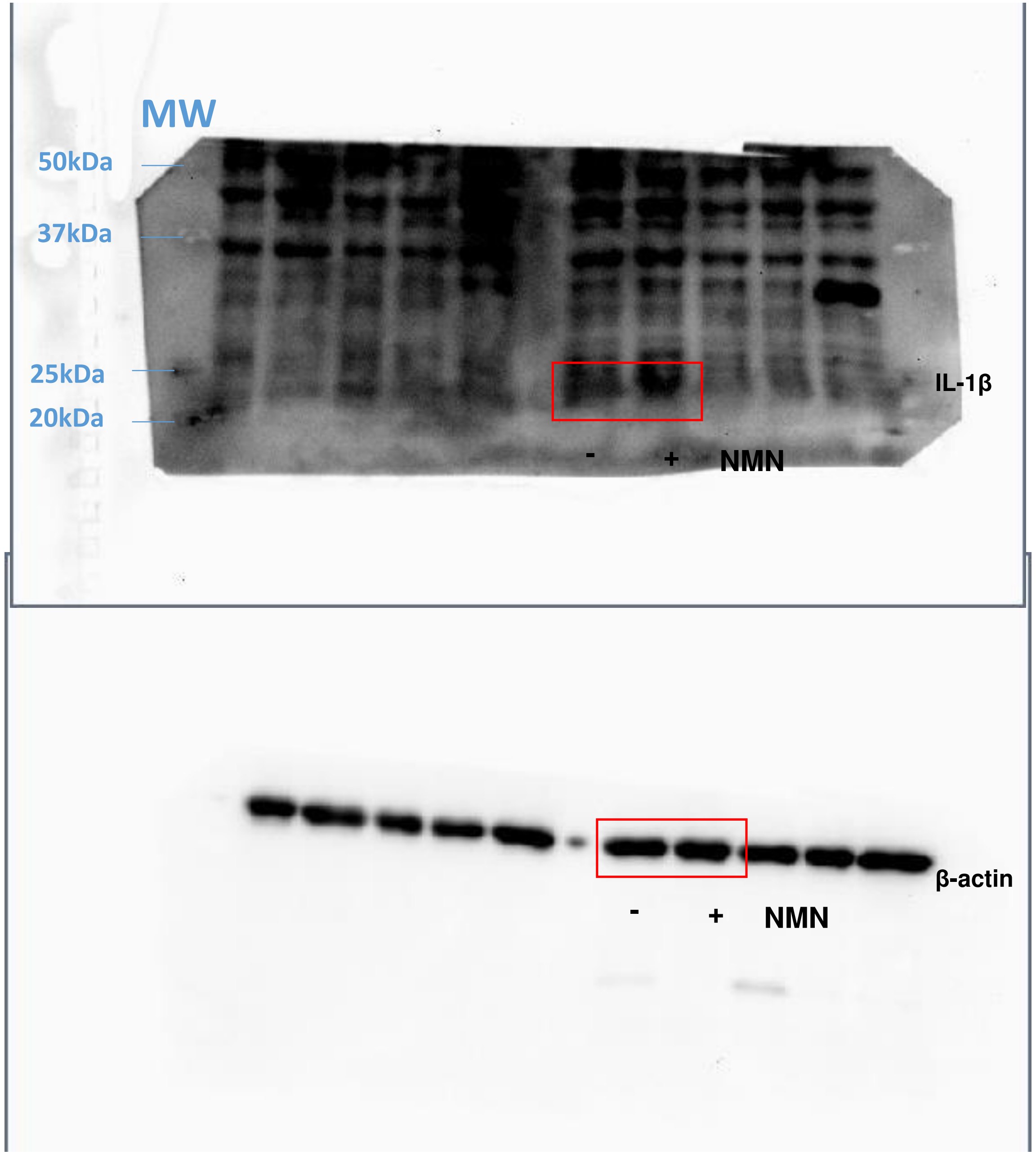
MW
50kDa
37kDa
25kDa
20kDa
IL-1β
- + NMN
β-actin
- + NMN

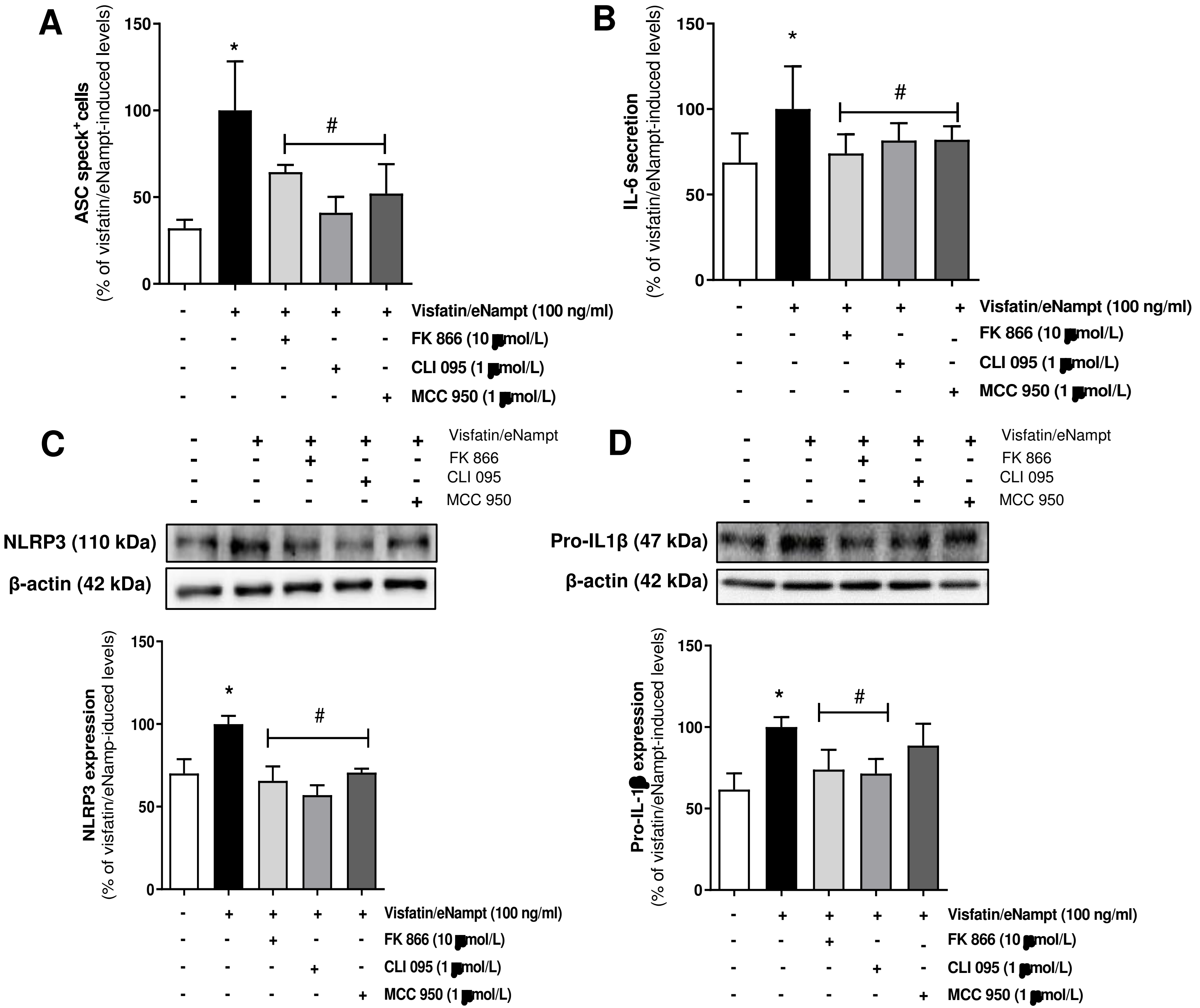
A
B
C
D
ASC speck+cells
(% of visfatin/eNampt-induced levels)
IL-6 secretion
(% of visfatin/eNampt-induced levels)
NLRP3 expression
(% of visfatin/eNamp-induced levels)
Pro-IL-1β expression
(% of visfatin/eNampt-induced levels)
0
50
100
150
*
#
Visfatin/eNampt (100 ng/ml)
FK 866 (10 µmol/L)
CLI 095 (1 µmol/L)
MCC 950 (1 µmol/L)
Visfatin/eNampt
FK 866
CLI 095
MCC 950
NLRP3 (110 kDa)
β-actin (42 kDa)
Pro-IL1β (47 kDa)

C

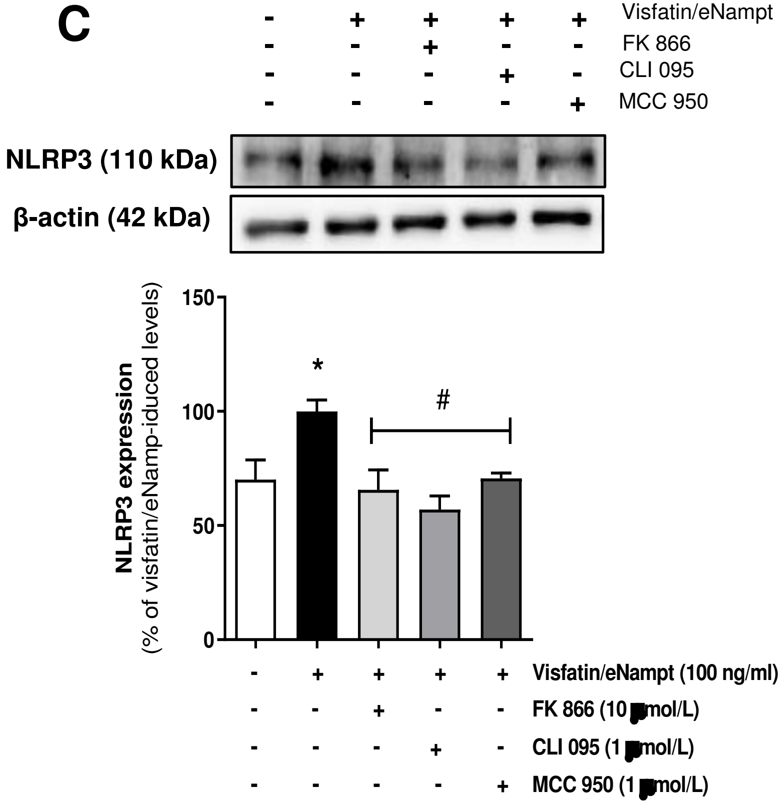

Visfatin/eNampt
FK 866
CLI 095
MCC 950
NLRP3 (110 kDa)
β-actin (42 kDa)
NLRP3 expression
(% of visfatin/eNamp-iduced levels)
150
100
50
0
*
#
Visfatin/eNampt (100 ng/ml)
FK 866 (10 µmol/L)
CLI 095 (1 µmol/L)
MCC 950 (1 µmol/L)

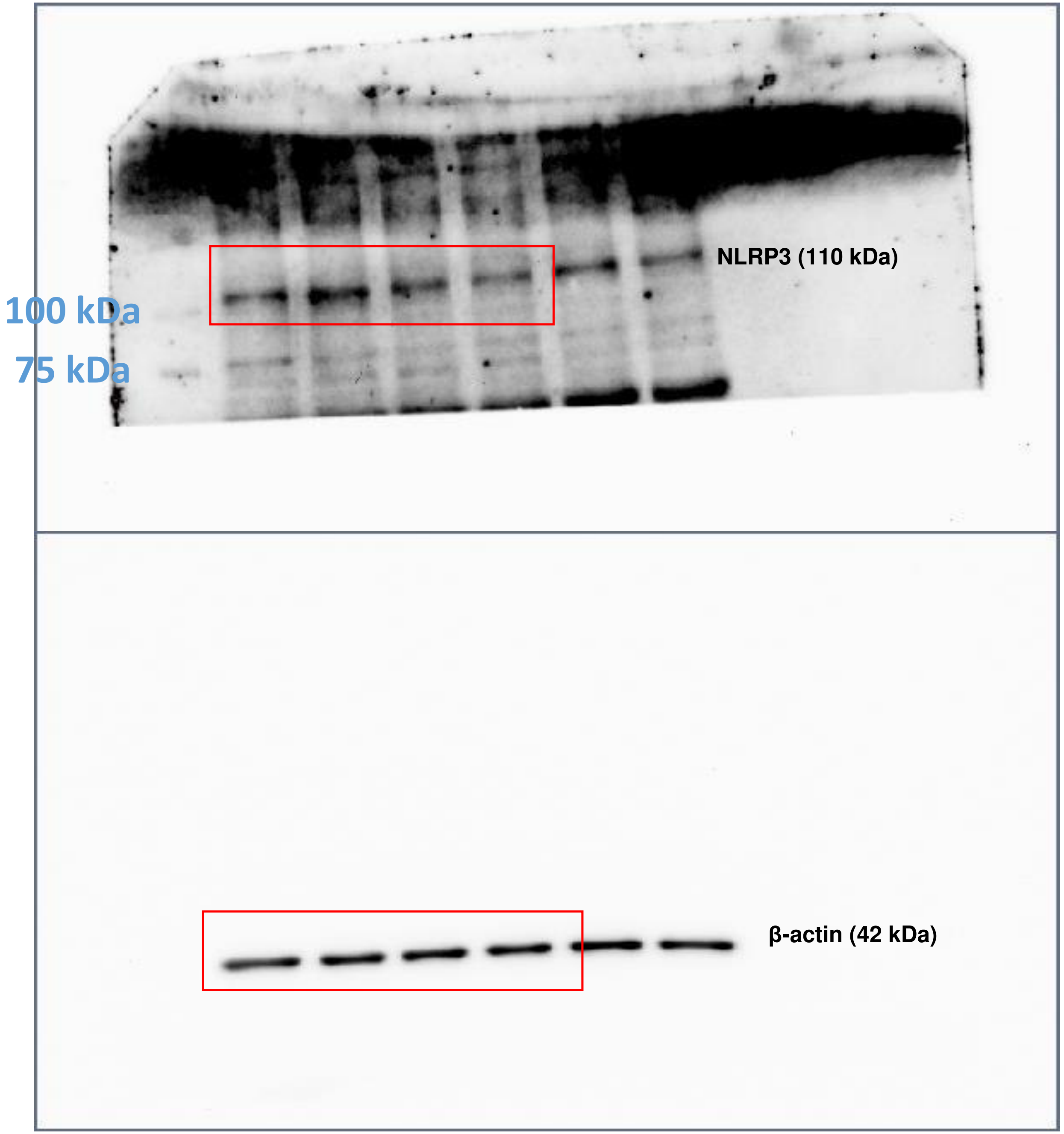

NLRP3 (110 kDa)
100 kDa
75 kDa
β-actin (42 kDa)

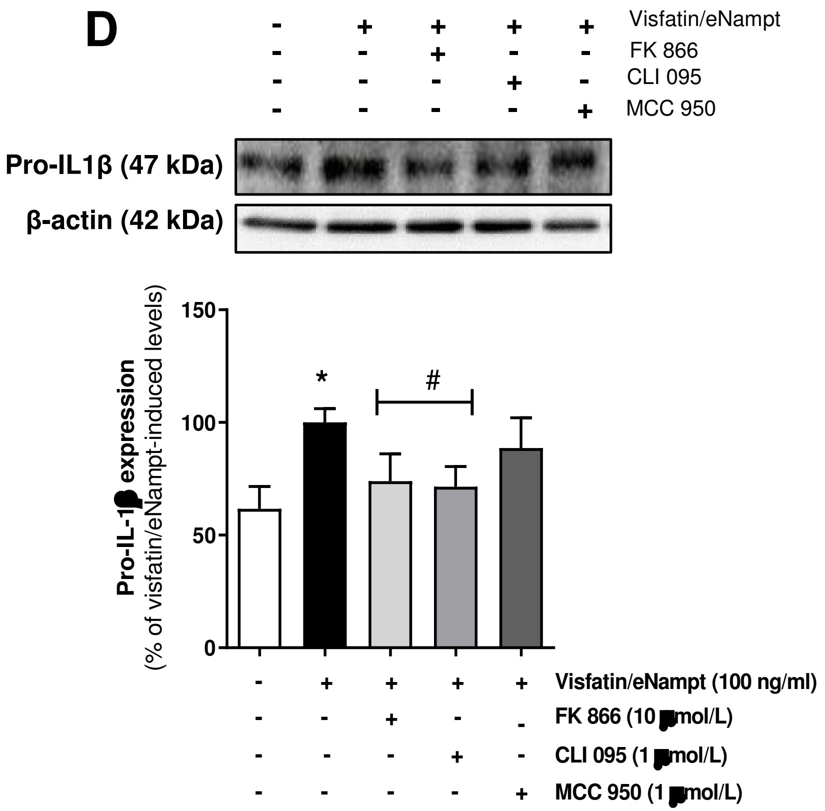
D
Visfatin/eNampt
FK 866
CLI 095
MCC 950
Pro-IL1β (47 kDa)
β-actin (42 kDa)
Pro-IL-1β expression
(% of visfatin/eNampt-induced levels)
150
100
50
0
*
#
Visfatin/eNampt (100 ng/ml)
FK 866 (10 μmol/L)
CLI 095 (1 μmol/L)
MCC 950 (1 μmol/L)

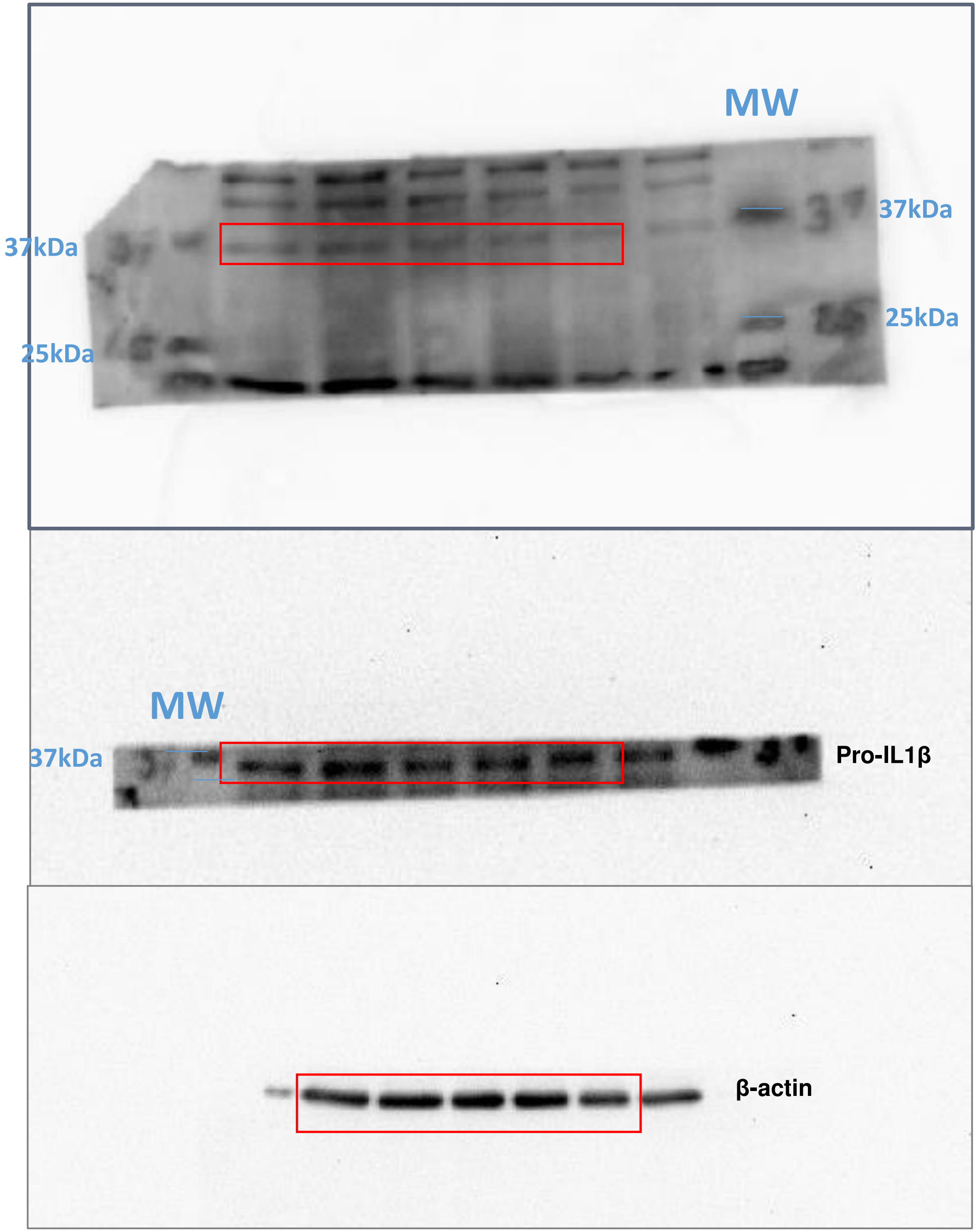
MW
37kDa
25kDa
37kDa
25kDa
MW
37kDa
Pro-IL1β
β-actin

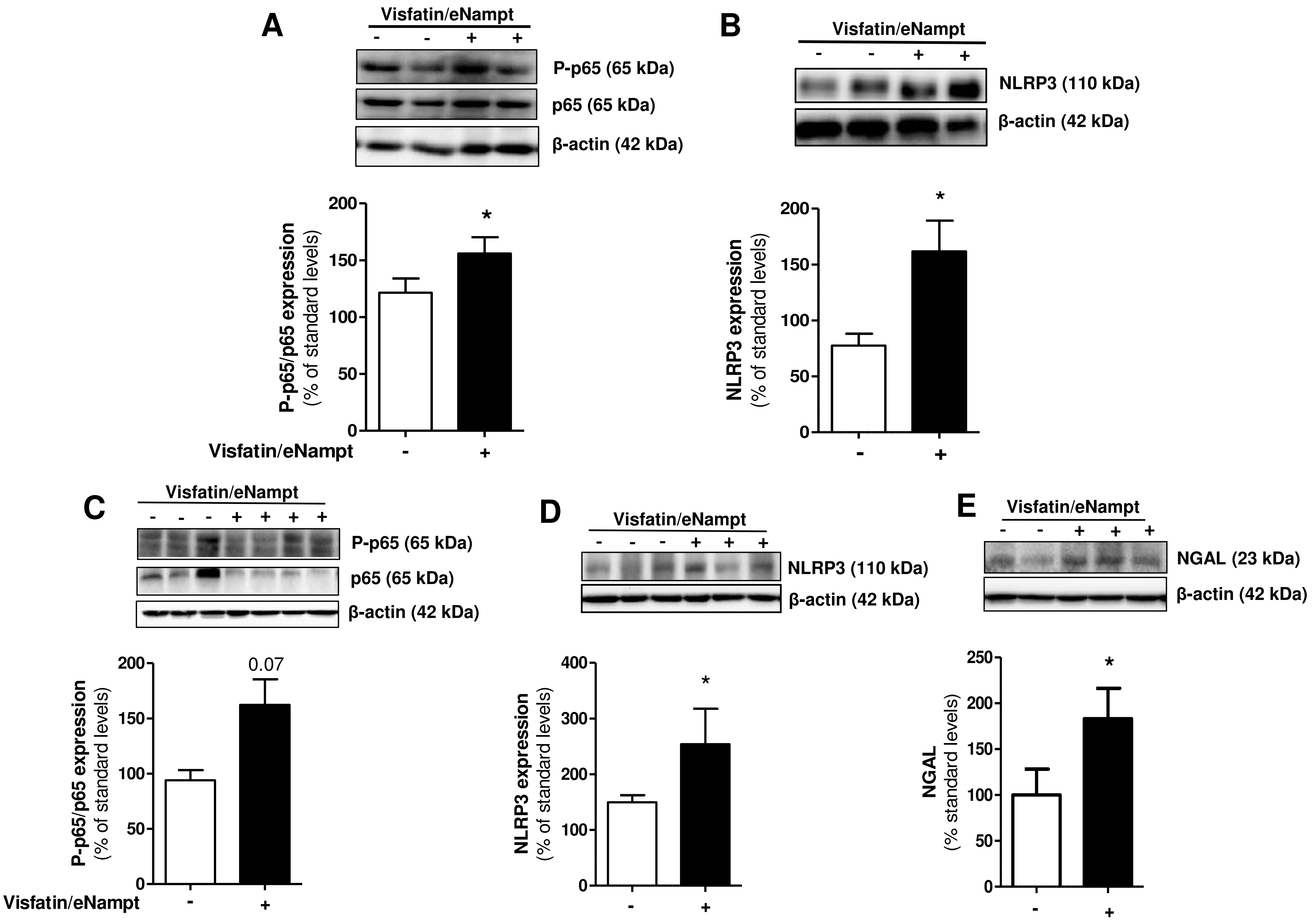
A
Visfatin/eNampt
- - + +
P-p65 (65 kDa)
p65 (65 kDa)
β-actin (42 kDa)
P-p65/p65 expression
(% of standard levels)
200
150
100
50
0
*
Visfatin/eNampt
-
+
B
Visfatin/eNampt
- - + +
NLRP3 (110 kDa)
β-actin (42 kDa)
NLRP3 expression
(% of standard levels)
200
150
100
50
0
*
-
+
C
Visfatin/eNampt
- - - + + + +
P-p65 (65 kDa)
p65 (65 kDa)
β-actin (42 kDa)
P-p65/p65 expression
(% of standard levels)
200
150
100
50
0
0.07
Visfatin/eNampt
-
+
D
Visfatin/eNampt
- - - + + +
NLRP3 (110 kDa)
β-actin (42 kDa)
NLRP3 expression
(% of standard levels)
400
300
200
100
0
*
-
+
E
Visfatin/eNampt
- - + + +
NGAL (23 kDa)
β-actin (42 kDa)
NGAL
(% standard levels)
250
200
150
100
50
0
*
-
+

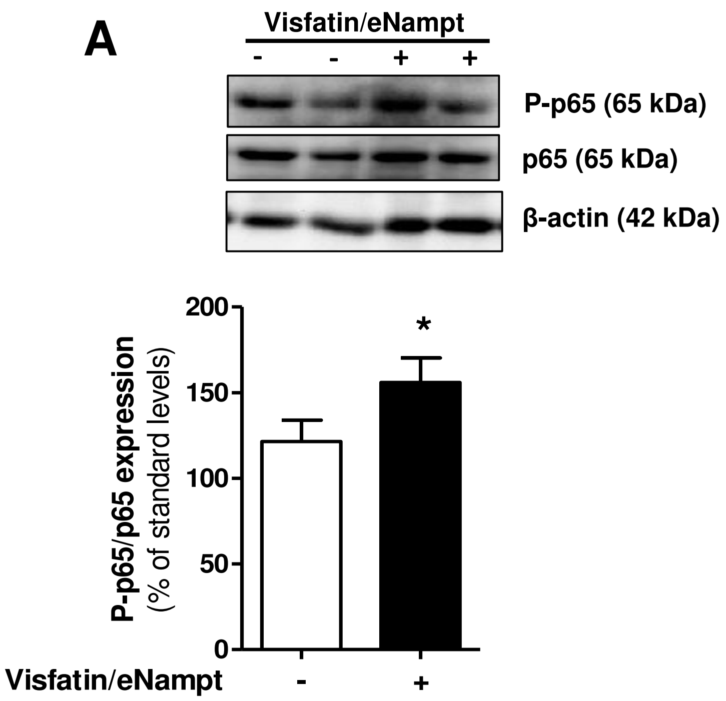
A
Visfatin/eNampt
- - + +
P-p65 (65 kDa)
p65 (65 kDa)
β-actin (42 kDa)
P-p65/p65 expression
(% of standard levels)
200
150
100
50
0
*
Visfatin/eNampt
-
+

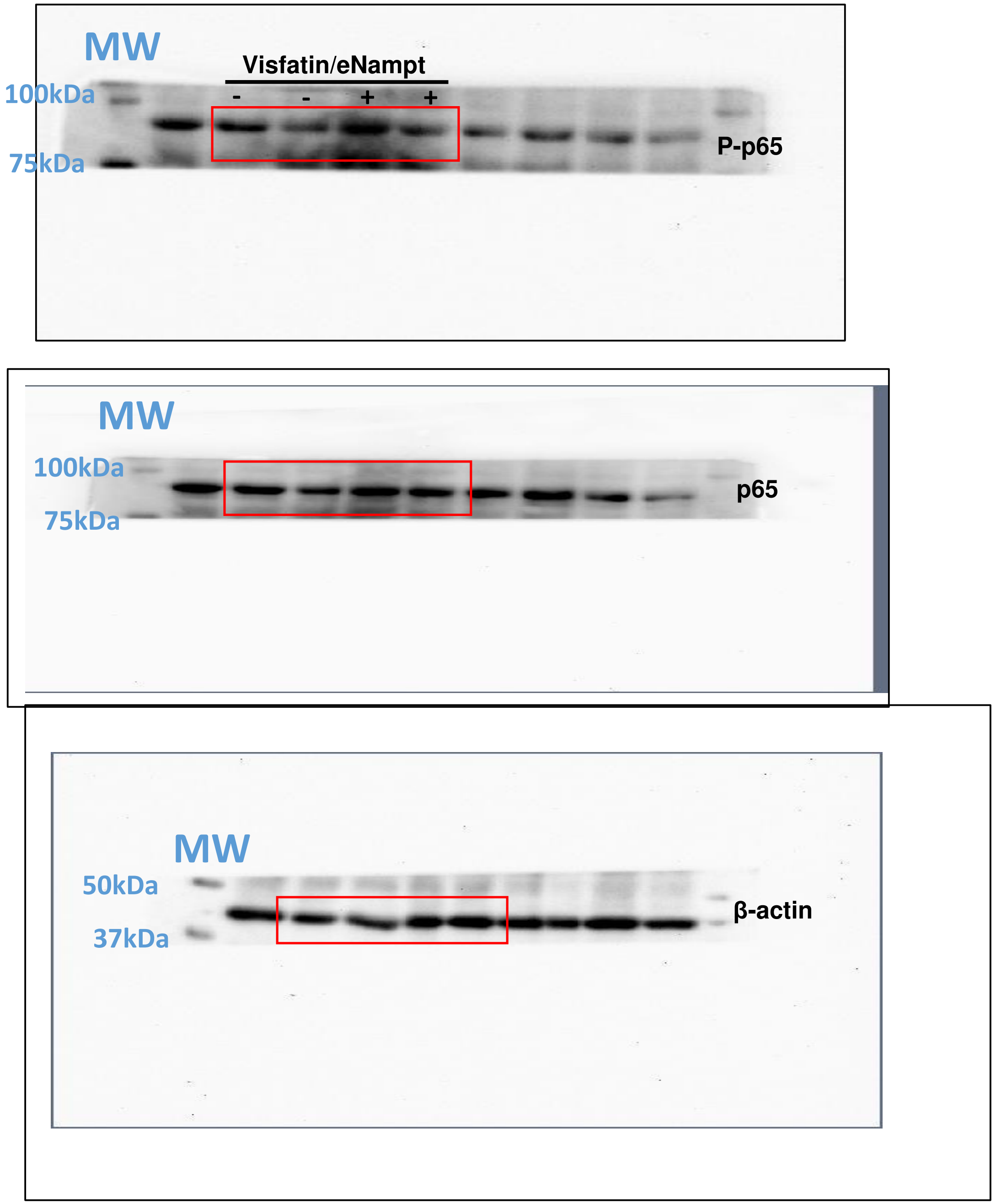
MW
Visfatin/eNampt
- - + +
100kDa
75kDa
P-p65
MW
100kDa
75kDa
p65
MW
50kDa
37kDa
β-actin

B

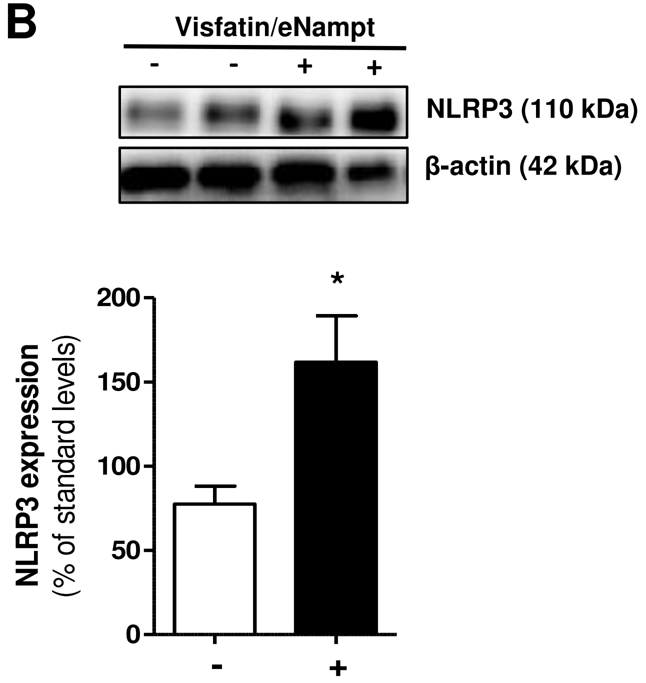

Visfatin/eNampt
- - + +
NLRP3 (110 kDa)
β-actin (42 kDa)
*
200
150
100
50
0
NLRP3 expression
(% of standard levels)
-
+

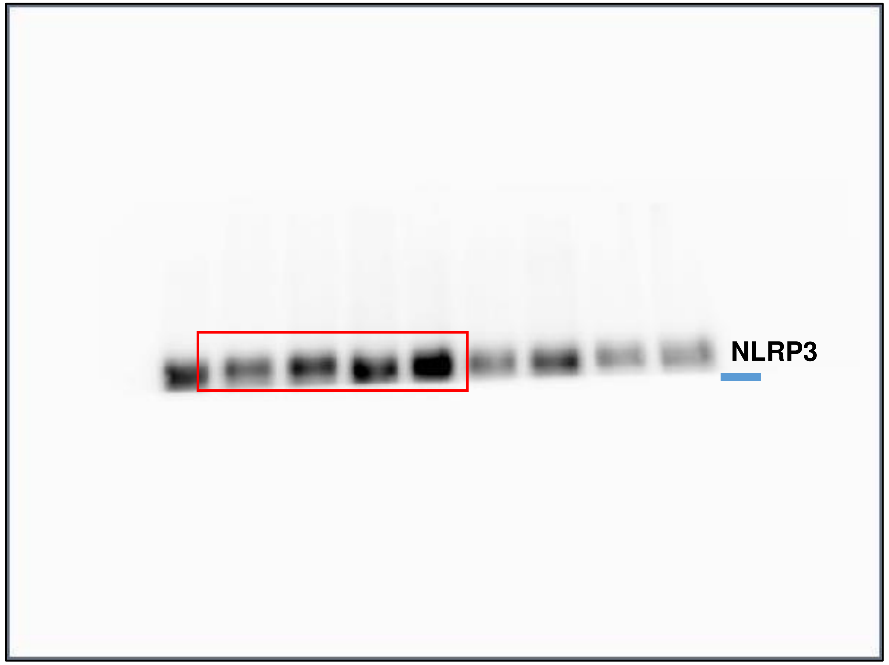

NLRP3

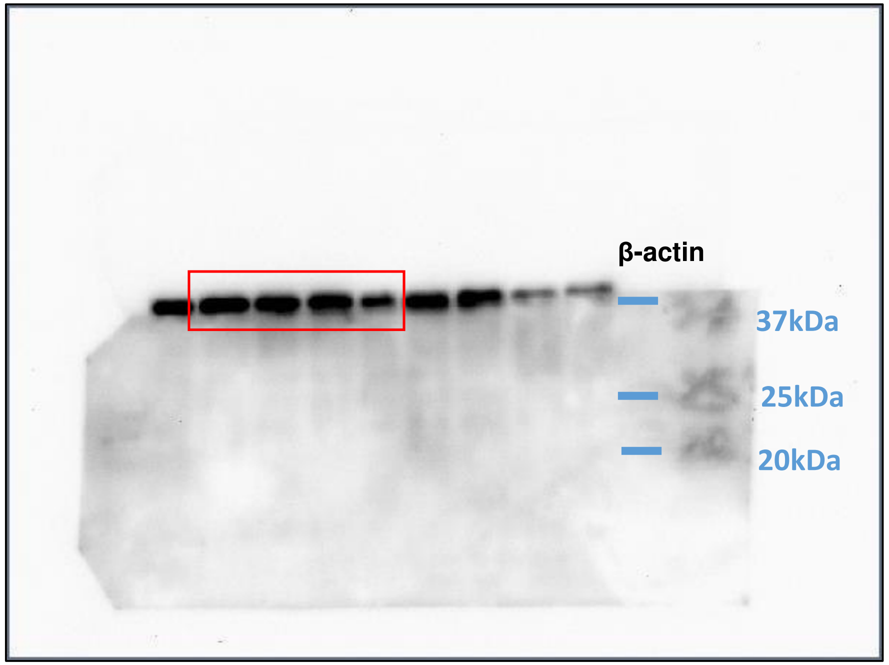

β-actin
37kDa
25kDa
20kDa

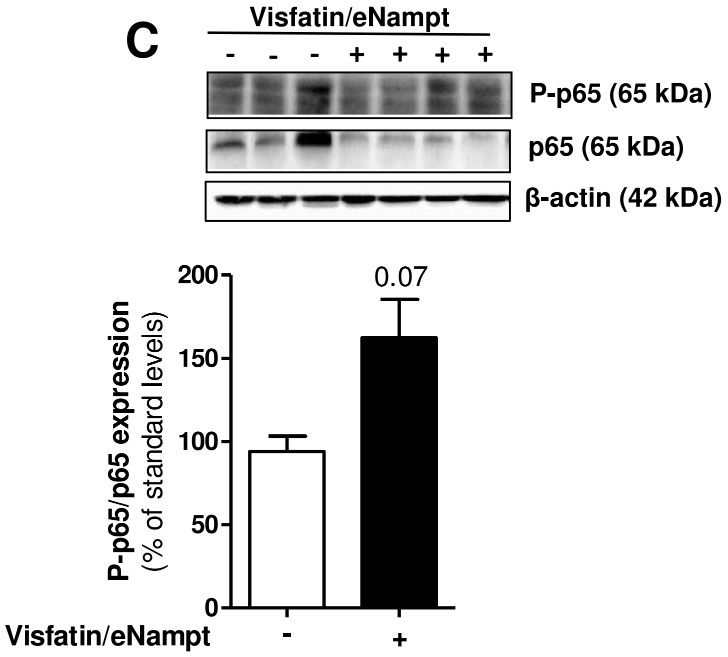
C
Visfatin/eNampt
- - - + + + +
P-p65 (65 kDa)
p65 (65 kDa)
β-actin (42 kDa)
200
150
100
50
0
0.07
P-p65/p65 expression
(% of standard levels)
Visfatin/eNampt
-
+

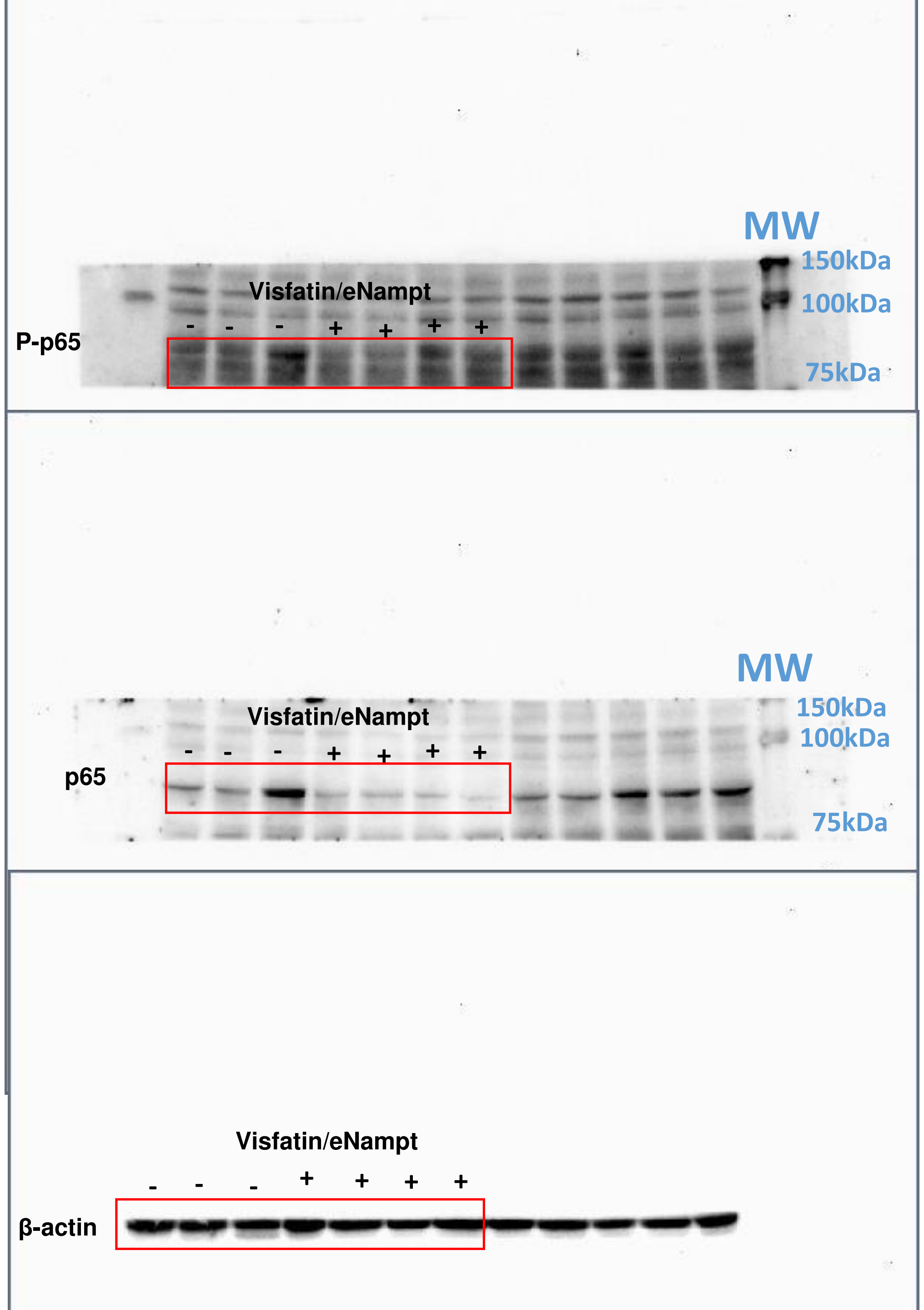
MW
150kDa
100kDa
75kDa
Visfatin/eNampt
- - - + + + +
P-p65
MW
150kDa
100kDa
75kDa
Visfatin/eNampt
- - - + + + +
p65
Visfatin/eNampt
- - - + + + +
β-actin

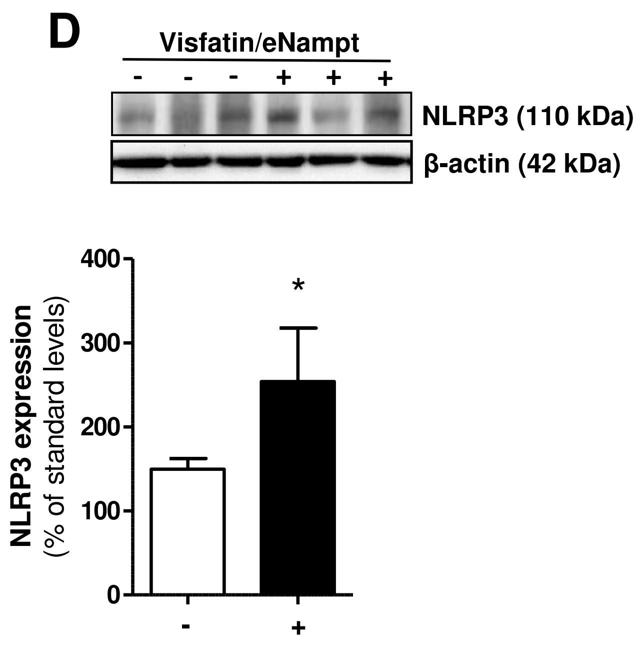
D
Visfatin/eNampt
- - - + + +
NLRP3 (110 kDa)
β-actin (42 kDa)
400
300
200
100
0
NLRP3 expression
(% of standard levels)
*
-
+

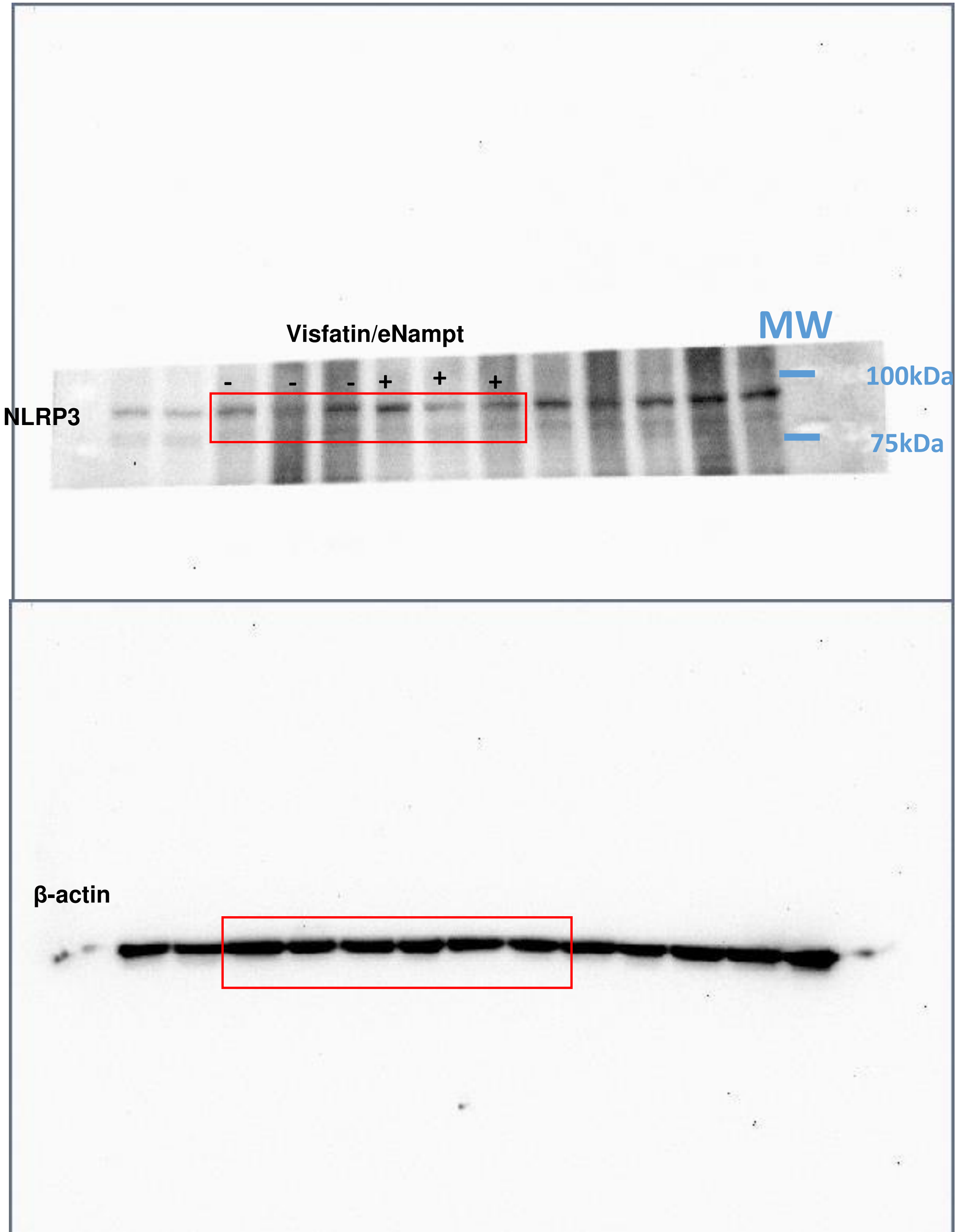
Visfatin/eNampt
MW
- - - + + +
NLRP3
100kDa
75kDa
β-actin

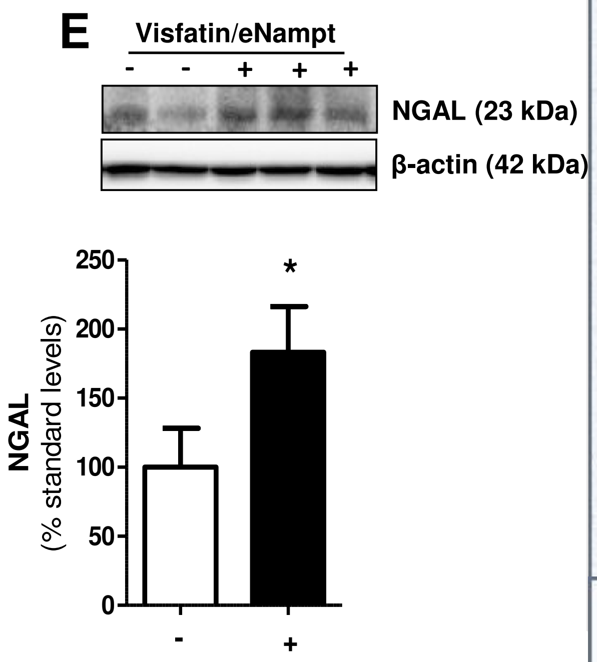
E
Visfatin/eNampt
- - + + +
NGAL (23 kDa)
β-actin (42 kDa)
250
200
150
100
50
0
NGAL
(% standard levels)
*
-
+

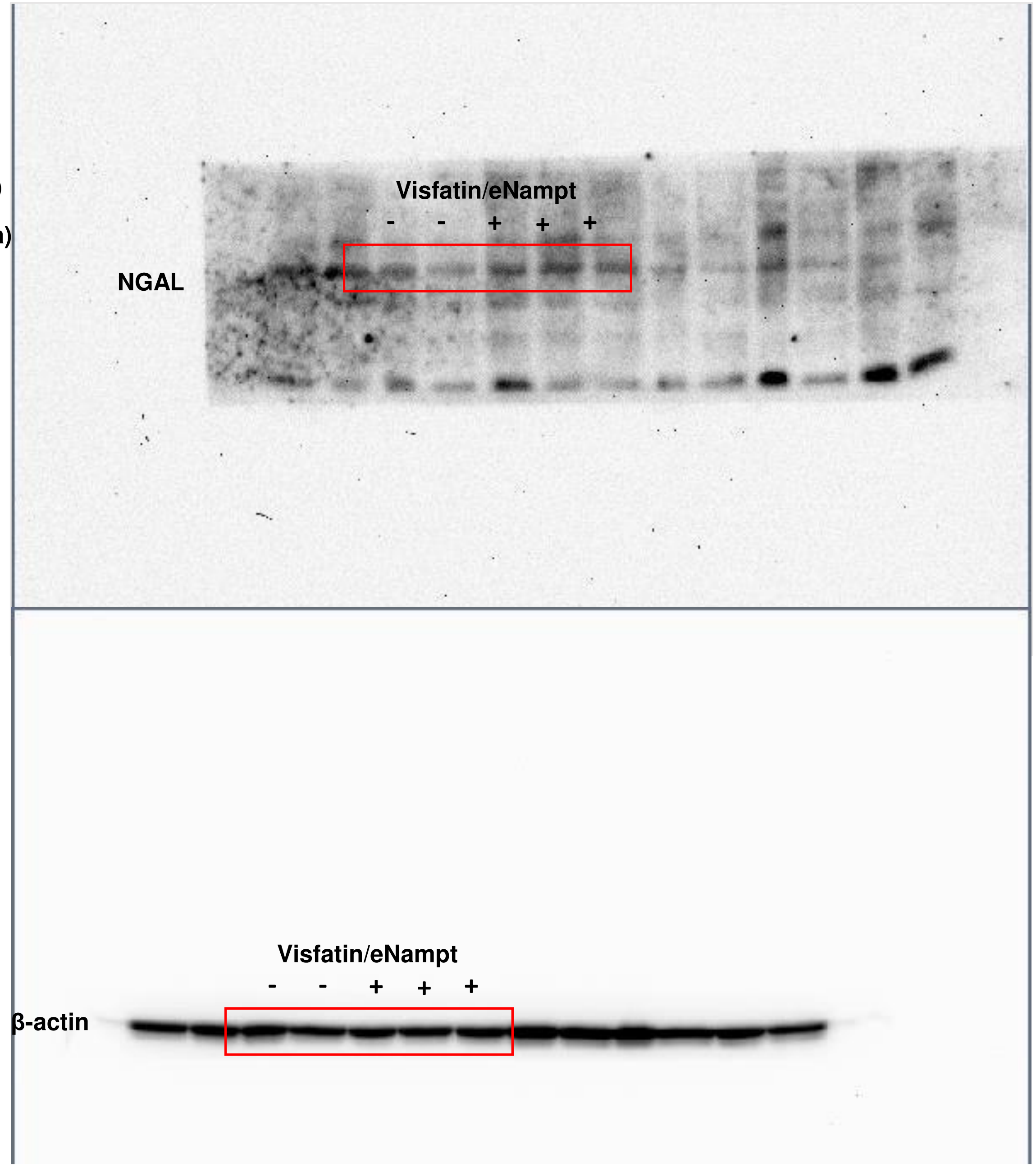
Visfatin/eNampt
- - + + +
NGAL
Visfatin/eNampt
- - + + +
β-actin

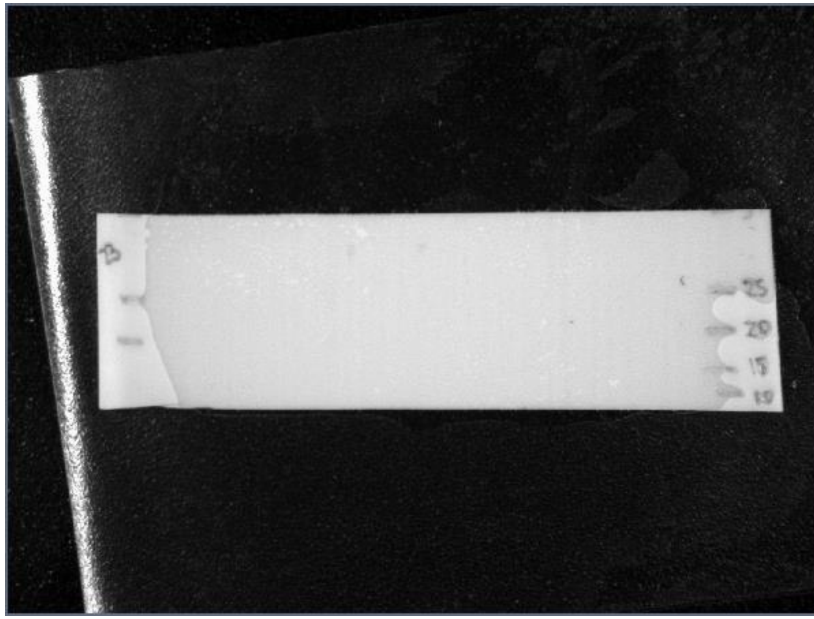

Figure S2

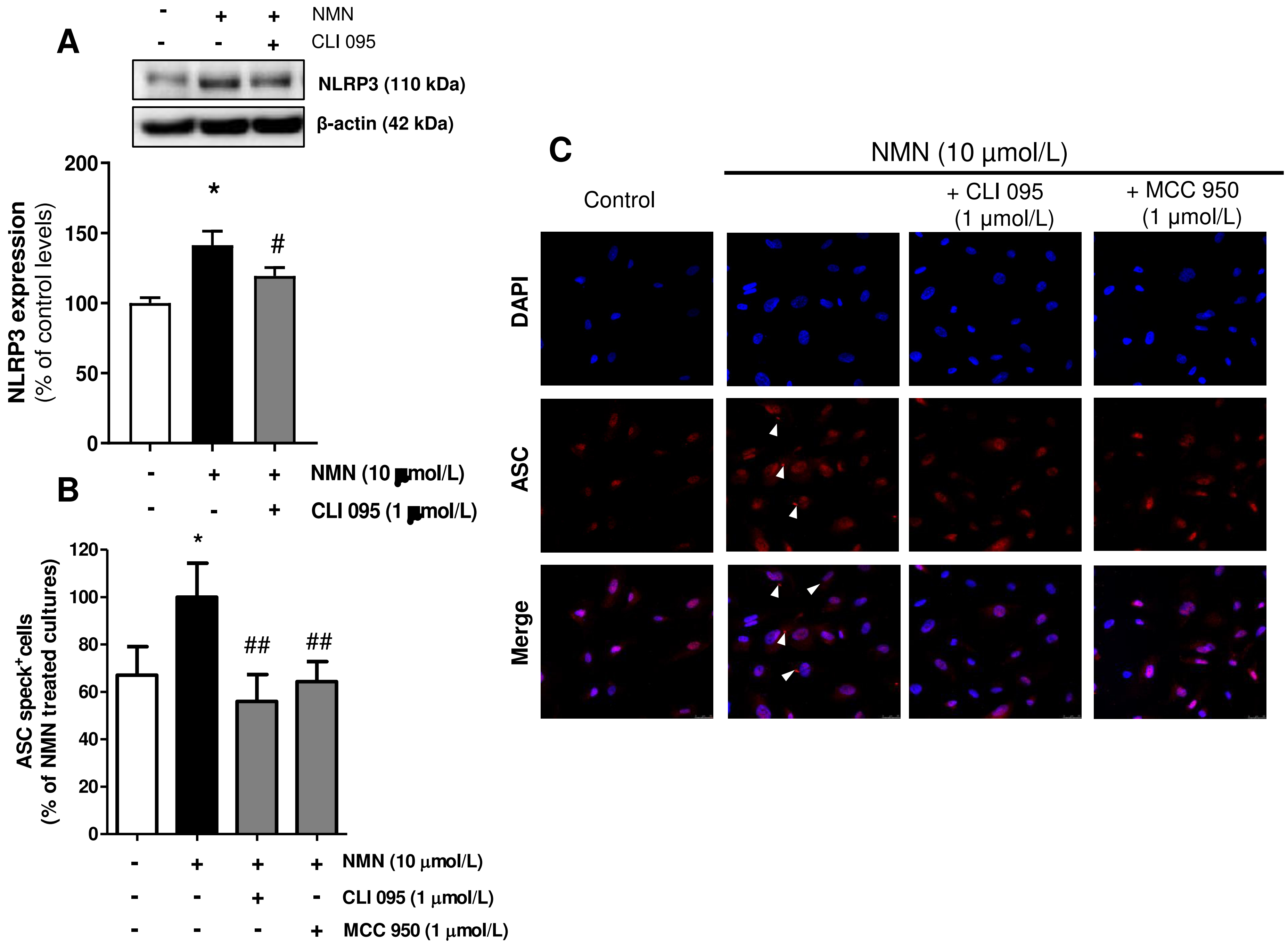
A
- + + NMN
- - + CLI 095
NLRP3 (110 kDa)
β-actin (42 kDa)
NLRP3 expression (% of control levels)
0
50
100
150
200
*
#
- + + NMN (10 μmol/L)
- - + CLI 095 (1 μmol/L)
B
ASC speck⁺cells (% of NMN treated cultures)
0
20
40
60
80
100
120
*
##
##
- + + + NMN (10 μmol/L)
- - + - CLI 095 (1 μmol/L)
- - - + MCC 950 (1 μmol/L)
C
NMN (10 μmol/L)
Control
+ CLI 095 (1 μmol/L)
+ MCC 950 (1 μmol/L)
DAPI
ASC
Merge

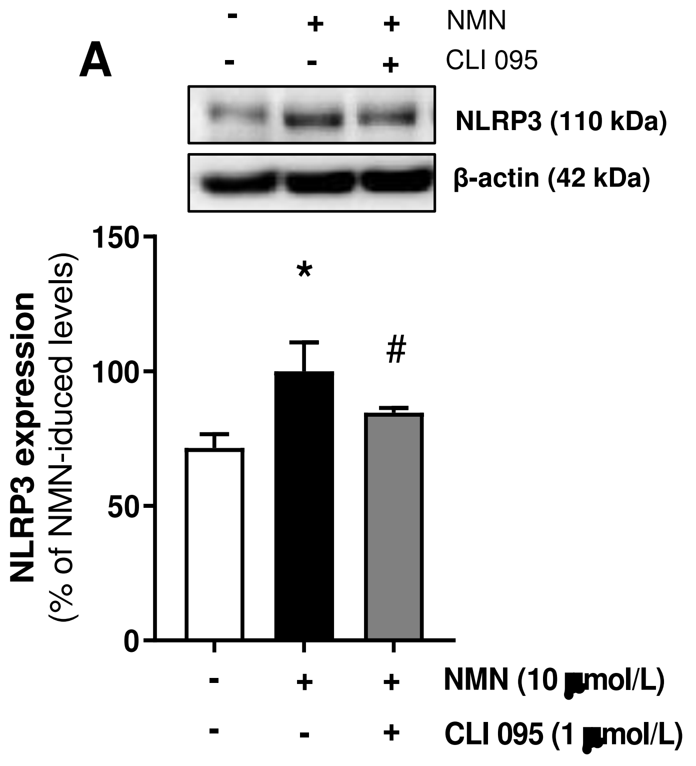
A
- + + NMN
- - + CLI 095
NLRP3 (110 kDa)
β-actin (42 kDa)
NLRP3 expression
(% of NMN-iduced levels)
150
100
50
0
*
#
- + + NMN (10 µmol/L)
- - + CLI 095 (1 µmol/L)

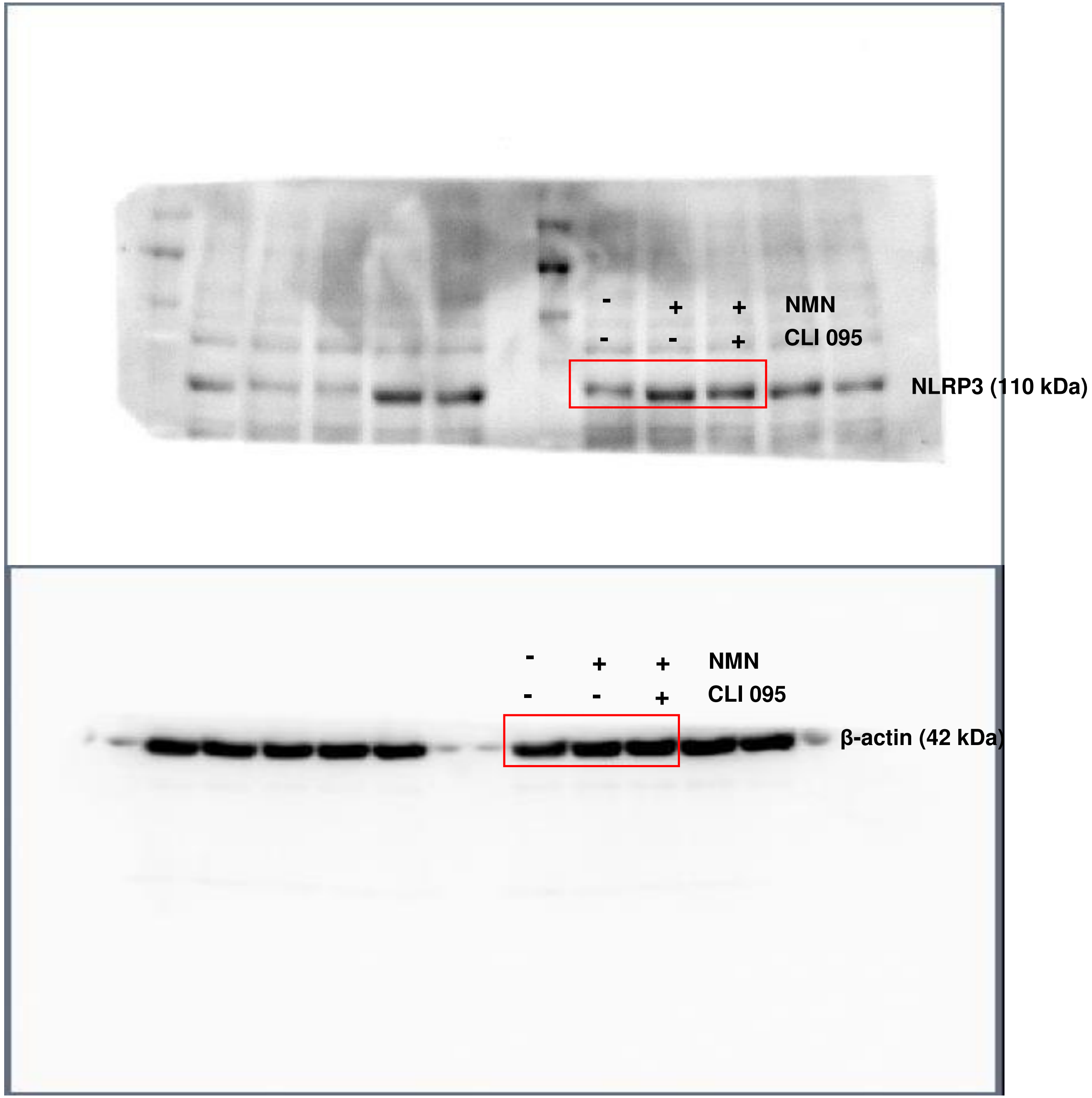
- + + NMN
- - + CLI 095
NLRP3 (110 kDa)
- + + NMN
- - + CLI 095
β-actin (42 kDa)
